# 5-Substituted Pyridine-2,4-dicarboxylate Derivatives Have Potential for Selective Inhibition of Human Jumonji-C Domain-Containing Protein 5

**DOI:** 10.1021/acs.jmedchem.3c01114

**Published:** 2023-08-01

**Authors:** Lennart Brewitz, Yu Nakashima, Sonia K. Piasecka, Eidarus Salah, Sally C. Fletcher, Anthony Tumber, Thomas P. Corner, Tristan J. Kennedy, Giorgia Fiorini, Armin Thalhammer, Kirsten E. Christensen, Mathew L. Coleman, Christopher J. Schofield

**Affiliations:** † Chemistry Research Laboratory, Department of Chemistry and the Ineos Oxford Institute for Antimicrobial Research, University of Oxford, 12 Mansfield Road, OX1 3TA Oxford, U.K.; ‡ Institute of Cancer and Genomic Sciences, University of Birmingham, Edgbaston, B15 2TT Birmingham, U.K.; § Chemical Crystallography, Chemistry Research Laboratory, Department of Chemistry, University of Oxford, 12 Mansfield Road, OX1 3TA Oxford, U.K.

## Abstract

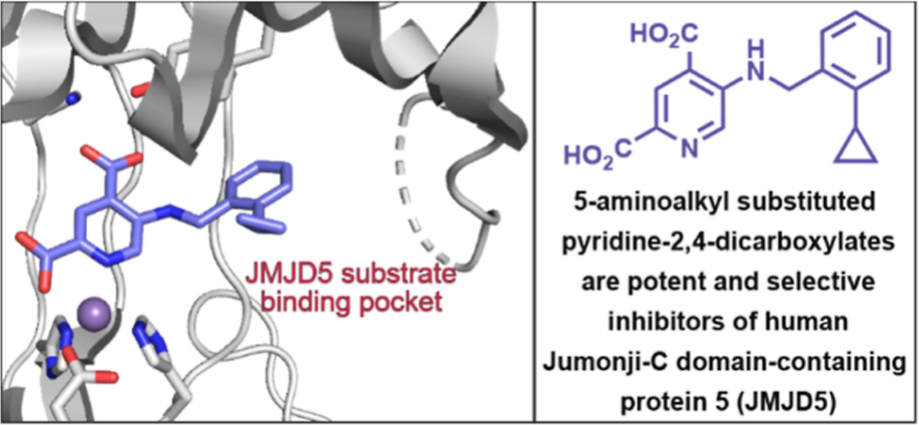

Jumonji-C domain-containing protein 5 (JMJD5) is a 2-oxoglutarate (2OG)-dependent oxygenase that plays important roles in development, circadian rhythm, and cancer through unclear mechanisms. JMJD5 has been reported to have activity as a histone protease, as an *N*^ε^-methyl lysine demethylase, and as an arginine residue hydroxylase. Small-molecule JMJD5-selective inhibitors will be useful for investigating its (patho)physiological roles. Following the observation that the broad-spectrum 2OG oxygenase inhibitor pyridine-2,4-dicarboxylic acid (2,4-PDCA) is a 2OG-competing JMJD5 inhibitor, we report that 5-aminoalkyl-substituted 2,4-PDCA derivatives are potent JMJD5 inhibitors manifesting selectivity for JMJD5 over other human 2OG oxygenases. Crystallographic analyses with five inhibitors imply induced fit binding and reveal that the 2,4-PDCA C5 substituent orients into the JMJD5 substrate-binding pocket. Cellular studies indicate that the lead compounds display similar phenotypes as reported for clinically observed JMJD5 variants, which have a reduced catalytic activity compared to wild-type JMJD5.

## Introduction

Jumonji-C (JmjC) domain-containing protein 5 (JMJD5) is a 2-oxoglutarate (2OG)- and Fe(II)-dependent oxygenase that is located in both the nucleus and cytoplasm of human cells.^1^ JMJD5 is a reported modulator of the cell cycle,^2^ of cellular metabolism,^3,4^ and of the mammalian^5,6^ (and plant)^5,7−9^ circadian rhythm; JMJD5 also regulates the replication cycle of hepatitis B virus in human cells.^10^ The importance of the biological functions of JMJD5 is highlighted by animal model studies, which have revealed severe embryonic growth retardation in homozygous JMJD5-deficient mice, ultimately resulting in embryonic lethality.^11−13^ Recently, biallelic JMJD5 variants have been identified in patients with developmental abnormalities, indicating that JMJD5 also plays an important role in human development.^14^ Animal model and cellular studies imply that JMJD5 enhances genome stability and acts as a tumor suppressor gene.^15,16^ In patients suffering from lung cancer^17^ or hepatocellular carcinoma,^18^ downregulation of JMJD5 correlates with reduced survival, suggesting a possible role of JMJD5 as a tumor suppressor.^19^ However, the roles of JMJD5 in human cancer progression are uncertain, because JMJD5 overexpression has also been correlated with poor patient prognosis, including in, e.g., colon cancer,^20^ breast cancer,^21^ non-small-cell lung cancer,^22^ and oral squamous cell carcinoma.^23^

Despite the important roles of JMJD5 in (human) biology, its exact molecular function has not yet been identified. Initially, JMJD5 was reported to catalyze the demethylation of histone 3 (H3) *N*^ε^-dimethylated lysine 36 (H3K36me2) similar to the JmjC lysine-specific *N*^ε^-demethylases 4A-C^24−27^ and was thus assigned as lysine-specific demethylase 8 (KDM8). JMJD5 has also been reported to possess protease activity and to cleave histone tails.^28−30^ Subsequently, however, the demethylase activity of JMJD5 in vitro and in cells has been questioned,^11,17^ including as a result of studies using purified isolated recombinant JMJD5 and peptide substrates.^31,32^ These observations are in accord with the reported lack of JMJD5 demethylase activity in mice^33^ and the structural similarity of JMJD5 with other human 2OG JmjC hydroxylases (which produce stable alcohol products), such as factor inhibiting hypoxia-inducible transcription factor (HIF-α) (FIH),^31,32^ rather than the JmjC KDMs, suggesting that JMJD5 may, in fact, be a protein hydroxylase giving a stable alcohol product.

**Figure 1:**
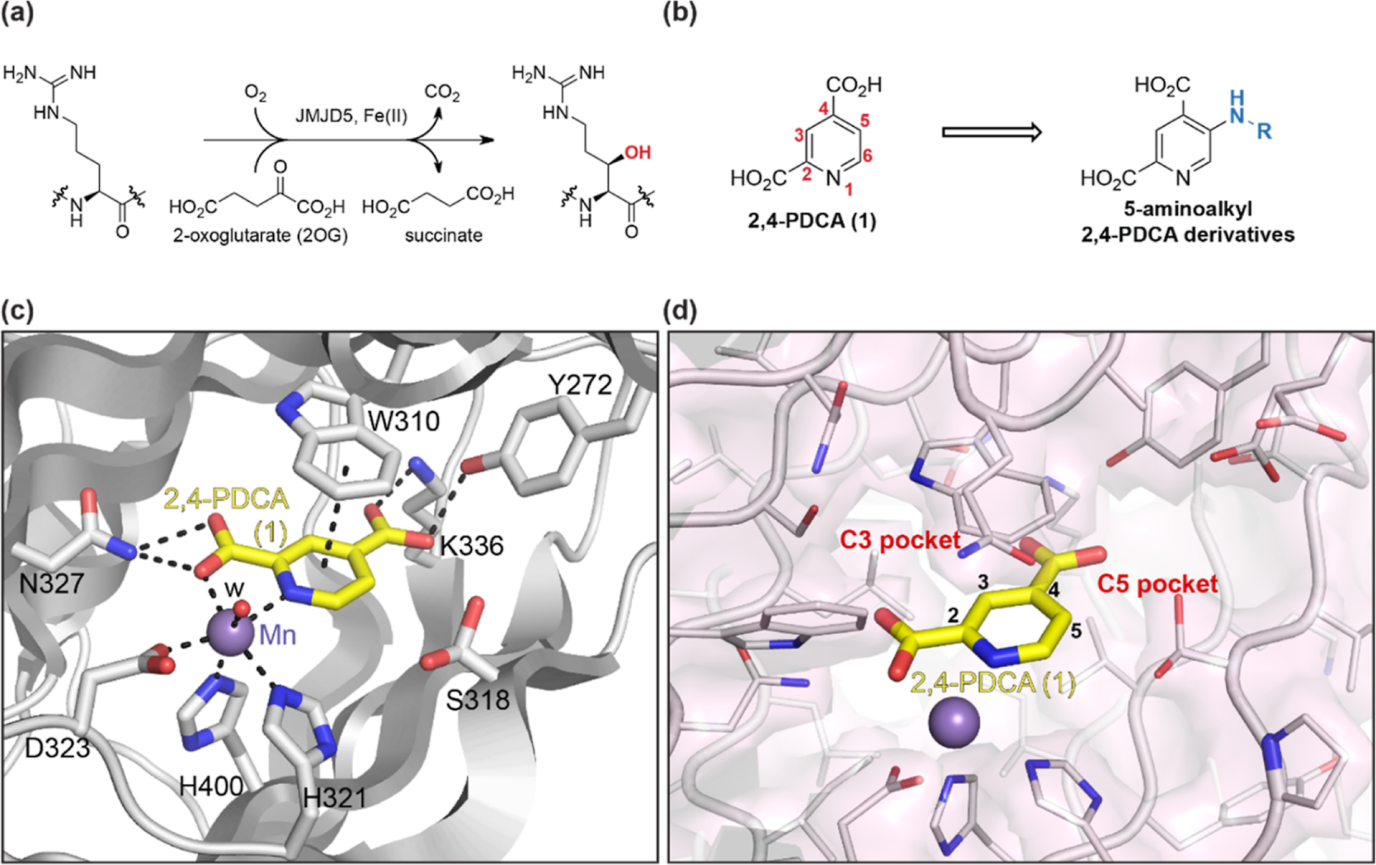
JMJD5 catalysis and inhibition. (a) Reaction scheme for JMJD5-catalyzed arginyl-residue C3 hydroxylation; (b) the broad-spectrum 2OG oxygenase inhibitor 2,4-PDCA (**1**) and the corresponding 5-aminoalkyl-substituted 2,4-PDCA derivatives; (c) active-site view of human JMJD5 complexed with 2,4-PDCA (**1**; PDB ID: 6I9L(36)), and (d) pockets adjacent to the C3 and C5 2,4-PDCA positions in the reported JMJD5:**1** complex structure (PDB ID: 6I9L(36)). Note that the hydroxymethylene group of S318 was refined in two orientations in this structure.^36^

We have shown that JMJD5 can catalyze the diastereospecific C3 hydroxylation of specific Arg-residues in synthetic peptides based on the sequences of human regulator of chromosome condensation domain-containing protein 1 (RCCD1; i.e., R141), with which it is reported to interact in cells,^25,34^ and the 40S ribosomal protein S6 (RPS6; i.e., R137) in vitro (Figure 1a).^35^ Mass spectrometry (MS)-based assays for JMJD5 were developed to investigate small molecules for JMJD5 inhibition that enabled the identification of the broad-spectrum 2OG oxygenase inhibitor pyridine-2,4-dicarboxylic acid (2,4-PDCA, 2,4-lutidinic acid; **1**; Figure 1b) as an efficient 2OG competing inhibitor, the binding mode of which was investigated by crystallography (Figure 1c).^36^ Note that the two carboxylates of **1** have a similar binding mode to those of 2OG.^36^

Selective small-molecule JMJD5 inhibitors have not yet been reported; their development will be useful for functional assignment studies aimed at deciphering the roles of JMJD5 in developmental biology, circadian rhythm, and cancer progression. 2OG oxygenases are validated drug targets, as shown by the development of HIF-α prolyl residue hydroxylase (PHD2) inhibitors and the entry of a broad-spectrum inhibitor of the KDM4 family of JmjC KDMs into clinical trials for the treatment of gastrointestinal cancers.^37−41^ Given its physiological importance, JMJD5 inhibitors are thus of considerable interest as potential therapeutics. Here, we report the synthesis of 5-aminoalkyl-substituted 2,4-PDCA derivatives, which are potent inhibitors of human JMJD5 and which display a superior selectivity profile compared to 2,4-PDCA (**1**) with respect to a functionally diverse set of human 2OG oxygenases. The mode of inhibition of the 2,4-PDCA derivatives was investigated using crystallography, the results of which show that the pyridine C5 substituent binds in the substrate-binding pocket. Consistent with potential target engagement in cells, we show the effects on cellular processes linked to JMJD5 function, including viability, cell-cycle progression, and replication fidelity.

## Results

### C3-Substituted 2,4-PDCA Derivatives Inhibit JMJD5

Human JMJD5 is inhibited by 2,4-PDCA (**1**), which is a 2OG mimic and which is a relatively broad-spectrum 2OG oxygenase inhibitor.^36,42,43^ However, the potency with which **1** inhibits 2OG oxygenases varies substantially.^44^ Hence, we envisaged that derivatives of **1** may be developed to obtain improved JMJD5-selective inhibitors. Analysis of the reported JMJD5:**1** complex structure (PDB ID: 6I9L(36)) indicated the presence of a pocket adjacent to the 2,4-PDCA C3 position, which may be sufficiently large to accommodate substituents (Figure 1d). Comparison of the JMJD5:**1** complex structure with those of other 2OG oxygenases suggested that binding to this pocket might be exploited to obtain selectivity over other human 2OG oxygenases.^45^ Therefore, a set of 22 reported C3-substituted 2,4-PDCA derivatives were investigated for inhibition of isolated recombinant JMJD5 using our recently reported solid-phase extraction (SPE) coupled to MS assay, which directly monitors the +16 Da mass shift associated with the JMJD5-catalyzed hydroxylation of an RPS6-derived peptide, i.e., RPS6_128–148_.^42^ The assay is suitable for the high-throughput screening of small molecules for JMJD5 inhibition, enabling the efficient determination of half-maximum inhibitory concentrations (IC_50_-values).^42^

We have reported that some C3-substituted 2,4-PDCA derivatives efficiently inhibit the 2OG oxygenase aspartate/asparagine-β-hydroxylase (AspH),^44,46^ which catalyzes the post-translational hydroxylation of specific aspartate and asparagine residues in epidermal growth factor-like domains (EGFDs),^47−49^ and/or the JmjC histone *N*^ε^-dimethyllysine-specific demethylase 4E (KDM4E).^44,50,51^ Both AspH and the KDM4 subfamily of JmjC KDMs are medicinal chemistry targets for the development of cancer therapeutics, and a KDM4 inhibitor is presently in clinical trials.^37,52−58^ Considering that 2,4-PDCA (**1**) inhibits isolated recombinant JMJD5 ∼15-fold less efficiently than AspH and with similar efficiency to KDM4E (Table 1, entry i), it was important to improve the selectivity of 2,4-PDCA for JMJD5 inhibition in order to identify molecules for use in biological and functional assignment studies.

**Table 1:**
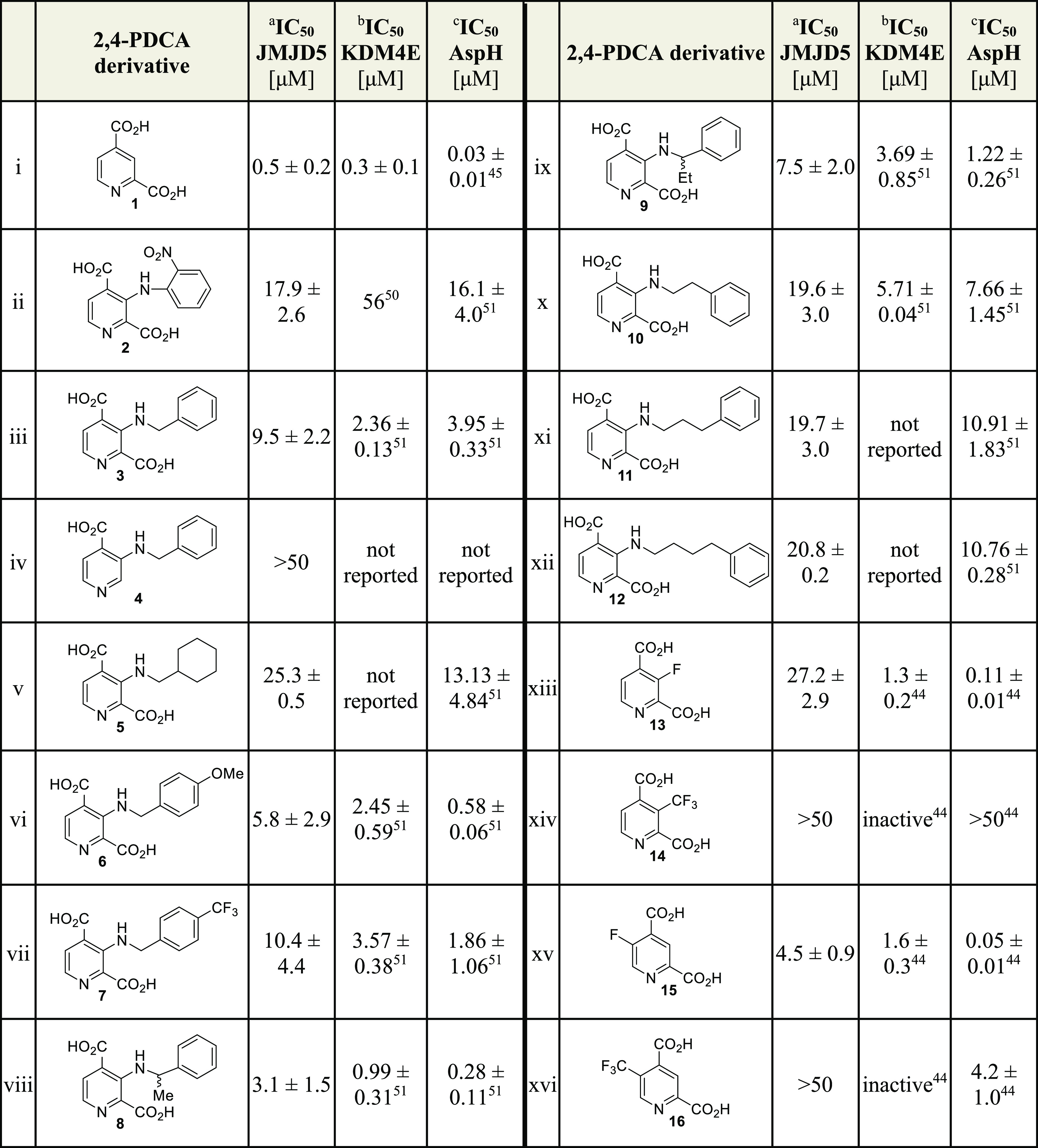
2,4-PDCA and Some C3-Substituted Derivatives Inhibit Human JMJD5 (the Complete Screening Results Are Shown in Supporting Information Table S1).

a Data are a mean of three independent runs (*n* = 3; mean ± standard deviation, SD); representative dose–response curves are shown in Supporting Information Figure S1. SPE-MS inhibition assays were performed as described, using JMJD5 (0.15 μM), 2OG (2.0 μM), Fe(II) (2.0 μM), LAA (100 μM), and RPS6_128–148_ (2.0 μM) in buffer (50 mM MOPS, pH 7.5, 20 °C).^42^ Z′ factors^59^ of all JMJD5 inhibition assays were >0.5, indicating excellent assay quality (Supporting Information Figure S2).

b Reported IC_50_ values were obtained using a formaldehyde dehydrogenase (FDH)-coupled, spectrophotometric turnover assay and SPE-MS inhibition assays for pyridine **2**(50) and pyridines **3**–**16**,^44,51^ respectively.

c Reported IC_50_ values were obtained using SPE-MS inhibition assays.^44,51^

d Chiral 2,4-PDCA derivatives were prepared as racemic mixtures.

The initial structure–activity relationship (SAR) studies revealed that the tested 3-aminoaryl-substituted 2,4-PDCA derivatives were, in general, inefficient inhibitors of isolated recombinant JMJD5 (i.e., IC_50_ > 20 μM, Supporting Information Table S1), with the exception of **2**, which inhibited with moderate potency (IC_50_ ∼ 17.9 μM; Table 1, entry ii). Importantly, **2** inhibited both JMJD5 and AspH with approximately similar potency, indicating that substituents on the 2,4-PDCA scaffold have the potential to alter the selectivity profile of 2,4-PDCA (**1**). Interestingly, at least some of the tested 3-aminoalkyl-substituted 2,4-PDCA derivatives inhibited JMJD5 more efficiently than all of the tested 3-aminoaryl substituted 2,4-PDCA derivatives (i.e., IC_50_ ≲ 10 μM), i.e., **3** and **6**–**9** (Table 1, entries iii and vi–ix). The results also revealed that the C2 carboxylate of 2,4-PDCA derivative **3** (and, by analogy, all other tested 2,4-PDCA derivatives) is important in JMJD5 inhibition because the related pyridine **4**, which lacks the C2 carboxylate, does not inhibit JMJD5 (Table 1, entry iv), in accord with the crystallographically observed role of the C2 carboxylate both in chelating the active site metal and in binding to N327 of JMJD5 (Figure 1).^36^

The results suggest that the phenyl group of the 3-aminobenzyl-substituted 2,4-PDCA derivative **3** (and, by analogy, derivatives **6**–**9**) is important for efficient JMJD5 inhibition; the 2,4-PDCA derivative **5**, in which the phenyl group is substituted for a cyclohexyl group, inhibits JMJD5 about twofold less efficiently than **3** (IC_50_ ∼ 25.3 μM; Table 1, entry v). The results show that the length of the alkylene unit connecting the phenyl group with the amine affects inhibition, i.e., 2,4-PDCA derivative **3**, in which a methylene unit connects the phenyl group with the amine, is approximately a twofold more potent JMJD5 inhibitor than those 2,4-PDCA derivatives bearing ethylene (**10**), propylene (**11**), or butylene (**12**) linkers (Table 1, entries x–xii).

Introducing electron-donating or -withdrawing groups to the phenyl ring of **3** at the position para to the methylene linker (**6**, **7**) did not have a substantial effect on JMJD5 inhibition (Table 1, entries vi and vii). By contrast, 2,4-PDCA derivative **8**, which bears a methyl substituent at the benzylic position α to the amine, was ∼3-fold more efficient in inhibiting JMJD5 than **3** but still ∼6-fold less efficient than 2,4-PDCA (IC_50_ ∼ 3.1 μM; Table 1, entry viii). Note, however, that **8** was used as a racemic mixture in the inhibition assays; thus, there is the possibility that one enantiomer of **8** inhibits JMJD5 more efficiently than the other. 2,4-PDCA derivative **9**, which bears an ethyl group that is sterically bulkier than a methyl group at the benzylic position α to the amine, showed reduced potency compared to **8** (IC_50_ ∼ 7.5 μM; Table 1, entry ix). This observation suggests that there is only a limited scope for substituents at the position α to the amine to develop more potent JMJD5 inhibitors.

Although the 3-aminoalkyl- and, to an apparently lesser extent, 3-aminoaryl-substituted 2,4-PDCA derivatives inhibit JMJD5 (Table 1), none of the tested C3-substituted 2,4-PDCA derivatives showed a selectivity profile that favored JMJD5 inhibition over that of AspH or KDM4E (Table 1). Hence, we investigated whether 2,4-PDCA derivatives bearing an electron-withdrawing fluoro or trifluoromethyl substituent instead of electron-donating 3-amino substituents at the 2,4-PDCA C3 or C5 position, i.e., **13**–**16**,^44^ are more selective in inhibiting JMJD5. Interestingly, the results reveal that 5-fluoro-2,4-PDCA (**15**) is approximately fivefold more efficient in inhibiting JMJD5 than the isomeric 3-fluoro-2,4-PDCA (**13**), but is approximately ninefold less efficient than 2,4-PDCA (**1**) (IC_50_ ∼ 4.5 μM; Table 1, entry xv). Also, note that **15** is reported to inhibit AspH with similar efficiency to **1**;^44^ thus, **15** is ∼90-fold more selective in inhibiting AspH than JMJD5 compared to **1**, which is only ∼15-fold more selective in inhibiting AspH than JMJD5. By contrast with the observed inhibition of JMJD5 by isomeric **13** and **15**, the corresponding C3 and C5 trifluoromethyl-substituted 2,4-PDCA derivatives **14** and **16** did not inhibit JMJD5 (IC_50_ > 50 μM; Table 1), implying that a fluorine substituent at the C3 or C5 position is preferred for JMJD5 inhibition over a sterically bulkier trifluoromethyl substituent. Remarkably, **16** has been reported to inhibit isolated recombinant AspH with moderate efficiency, whereas the C3 isomer **14** does not inhibit; both **14** and **16** have been reported not to inhibit KDM4E (Table 1).^44^

In addition to the 2,4-PDCA (**1**) C3 adjacent pocket, analysis of the JMJD5:**1** complex structure (PDB ID: 6I9L(36)) indicates that the JMJD5 active site may be sufficiently large to accommodate substituents at the C5 and/or C6 positions of **1**. The structure implies that only the side chain of S318 is located close to the 2,4-PDCA C5 position (Figure 1d). Although it was considered possible that a C5 substituent of **1** may interfere with the S318 side chain, the side chain of S318 was observed in two alternative conformations in the JMJD5:**1** complex structure,^36^ suggesting that the S318 hydroxymethyl group can occupy a conformation in which it faces away from a 2,4-PDCA C5 substituent. The observation that 5-fluoro-2,4-PDCA (**15**) inhibits isolated recombinant JMJD5 more efficiently than the isomeric 3-fluoro-2,4-PDCA (**13**), together with the analysis of the reported JMJD5:**1** complex structure, thus suggested to us that 5-aminoalkyl-substituted 2,4-PDCA derivatives have the potential to be more efficient in inhibiting human JMJD5 compared to the tested corresponding C3 isomers, thus potentially enabling an improved selectivity profile. Since 5-aminoalkyl-substituted 2,4-PDCA derivatives have not yet been described, we developed a synthetic route to access 5-aminoalkyl-substituted 2,4-PDCA derivatives.

### Synthesis of 5-Aminoalkyl-substituted 2,4-PDCA Derivatives

**Scheme 1:**
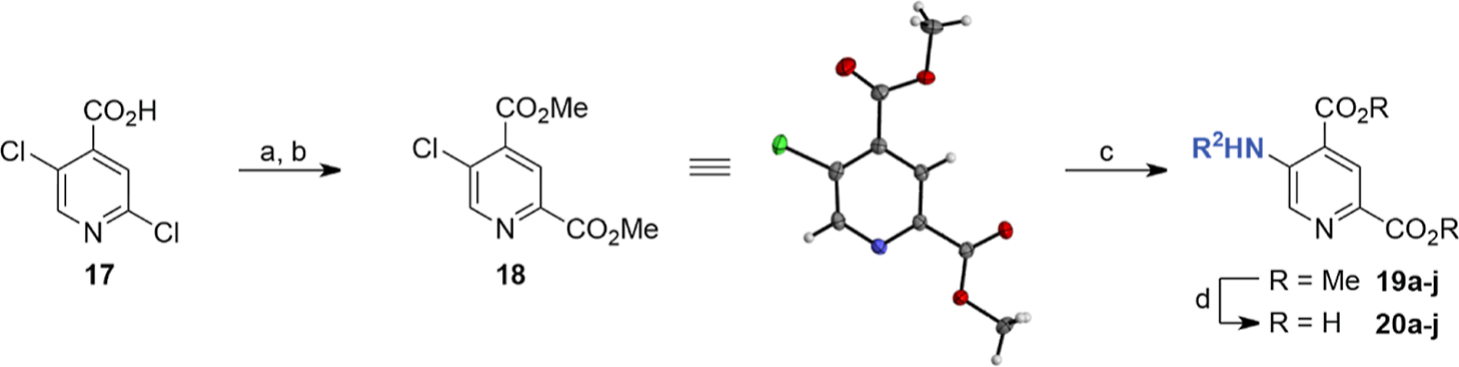
Synthesis of 5-Aminoalkyl-substituted 2,4-PDCA Derivatives Reagents and conditions: (a) SOCl_2_, MeOH, reflux, 95%; (b) CO (1.5 atm), Cl_2_Pd-*rac*-BINAP (1 mol %), Hünig’s base,^61^ MeOH, 100 °C (sand bath temperature, sealed flask), 81%; (c) amine (R^2^NH_2_; 3.0 equiv), Pd(OAc)_2_ (4 mol %), Josiphos SL-J009-1^62,63^ (5 mol %), Hünig’s base,^61^ 1,4-dioxane, 170 °C (sand bath temperature, sealed flask), 17–58%; and (d) LiOH, MeOH/H_2_O, 0 °C to rt, 47–93%. Crystal structure color code: white: hydrogens; gray: carbons; blue: nitrogen; red: oxygens; and green: chlorine.

The synthesis of 5-aminoalkyl-substituted 2,4-PDCA derivatives was achieved from commercially sourced 2,5-dichloroisonicotinic acid (**17**) (Scheme 1), in an analogous manner to our reported synthetic strategy for the synthesis of the corresponding isomeric 3-substituted 2,4-PDCA derivatives.^51^ Acid **17** was initially converted to its methyl ester, which was then subjected to a regioselective Pd-catalyzed carbonylation reaction^60^ to afford a mixture of dimethyl 5-chloropyridine-2,4-dicarboxylate (**18**) and trimethyl pyridine-2,4,5-tricarboxylate (Scheme 1). Following purification by column chromatography, **18** was obtained in a 77% yield over two steps as a single regioisomer; its structure was confirmed by single-crystal X-ray diffraction studies (Supporting Information Table S2).

The subsequent Buchwald–Hartwig amination^64,65^ of dimethyl 5-chloropyridine-2,4-dicarboxylate (**18**) with *N*-alkylamines required optimization, as the reported reaction conditions for the Buchwald–Hartwig amination of the isomeric dimethyl 3-chloropyridine-2,4-dicarboxylate, which employed a catalyst system composed of Pd(OAc)_2_ as metal source and Xantphos^66^ as ligand,^51^ did not afford the purified products in satisfactory yields. A ligand screen revealed that using Josiphos (SL-J009-1)^62,63^ as a palladium ligand improved the conversion to the target materials and reduced the formation of undesired byproducts; note that the commercially sourced enantiopure Josiphos SL-J009-1 ligand was used for this achiral transformation. A solvent screen revealed that the Buchwald–Hartwig amination was most efficient in ethereal solvents because of the limited solubility of **18** in other solvents including toluene, which was used for the Buchwald–Hartwig amination of the isomeric dimethyl 3-chloropyridine-2,4-dicarboxylate.^51^ Ester **18** was more soluble in 1,4-dioxane than in THF or DME, resulting in higher yields of the amination products. Finally, investigating the effect of the base on the conversion revealed that using Hünig’s base (*N*,*N*-diisopropylethylamine)^61^ rather than pyridine further improved the reaction yield. Crystallographic analysis of a Buchwald–Hartwig reaction product confirmed the anticipated regioselectivity of the amination reaction under the optimized reaction conditions (Supporting Information Figure S3 and Table S2).

The efficiency of the optimized Buchwald–Hartwig amination reaction was also found to depend on the reactivity of the amine employed. Productive amination was achieved using linear alkylamines, whereas the use of α-branched alkylamines resulted in poor yields, presumably due to steric hindrance. Anilines were not suitable nucleophiles for this Buchwald–Hartwig transformation. Thus, a set of 10 5-aminoalkyl-substituted 2,4-PDCA derivatives was synthesized for SAR studies aimed at identifying selective JMJD5 inhibitors.

**Figure 2:**
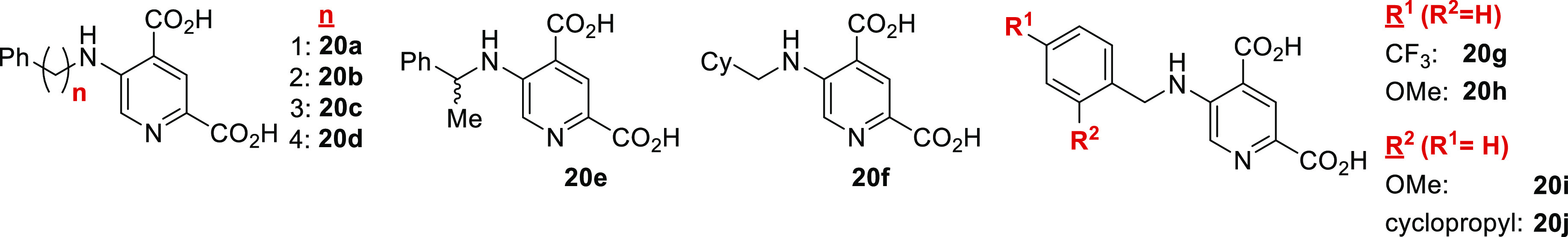
Structures of 5-aminoalkyl-substituted 2,4-PDCA derivatives **20a**–**j** (pyridine **20e** was prepared as a racemic mixture).

The Buchwald–Hartwig amination reaction diester products, i.e., dimethyl 5-aminoalkyl-substituted 2,4-PDCA derivatives **19a**–**j**, are potentially useful as prodrugs for cell-based experiments,^67,68^ while their corresponding dicarboxylic acids are required for in vitro biochemical and biophysical experiments. Note that dimethyl 2,4-PDCA does not inhibit isolated recombinant JMJD5 (Supporting Information Table S1), supporting the proposal that cellular esterases are required to convert the dimethyl prodrugs into their active diacid form. The dimethyl esters **19a**–**j** were subjected to lithium hydroxide-mediated saponification to afford the desired 5-aminoalkyl-substituted 2,4-PDCA derivatives **20a**–**j** in 47–93% yield (Figure 2); the excess base was removed by acidic ion exchange chromatography to yield the salt-free inhibitors suitable for JMJD5 inhibition and crystallization studies.

### 5-Aminoalkyl-Substituted 2,4-PDCA Derivatives Inhibit JMJD5

The ability of the synthetic 5-aminoalkyl-substituted 2,4-PDCA derivatives to inhibit isolated recombinant human JMJD5 was investigated using reported SPE-MS inhibition assays (Table 2).^42^ The results revealed that 9 of the 10 investigated 2,4-PDCA derivatives (i.e., **20a**–**d** and **20f**–**j**) inhibit JMJD5 with similar efficiency (IC_50_ ∼ 0.3 to 0.9 μM; Table 2) as 2,4-PDCA (**1**; IC_50_ ∼ 0.5 μM; Table 2, entry i). A notable exception is 2,4-PDCA derivative **20e**, which inhibits JMJD5 approximately 35-fold less efficiently than **1** (IC_50_ ∼ 17.5 μM; Table 2, entry iii). Thus, it appears that introducing an aminoalkyl substituent at the 2,4-PDCA C5 position does not substantially affect JMJD5 inhibition unless the steric bulk of the substituent in proximity of the 2,4-PDCA C5 position increases, in accord with the previous result that 5-trifluoromethyl-2,4-PDCA **16** does not inhibit JMJD5, while 5-fluoro-2,4-PDCA **15** inhibits (Table 1, entries xvi and xv, respectively).

**Table 2:**
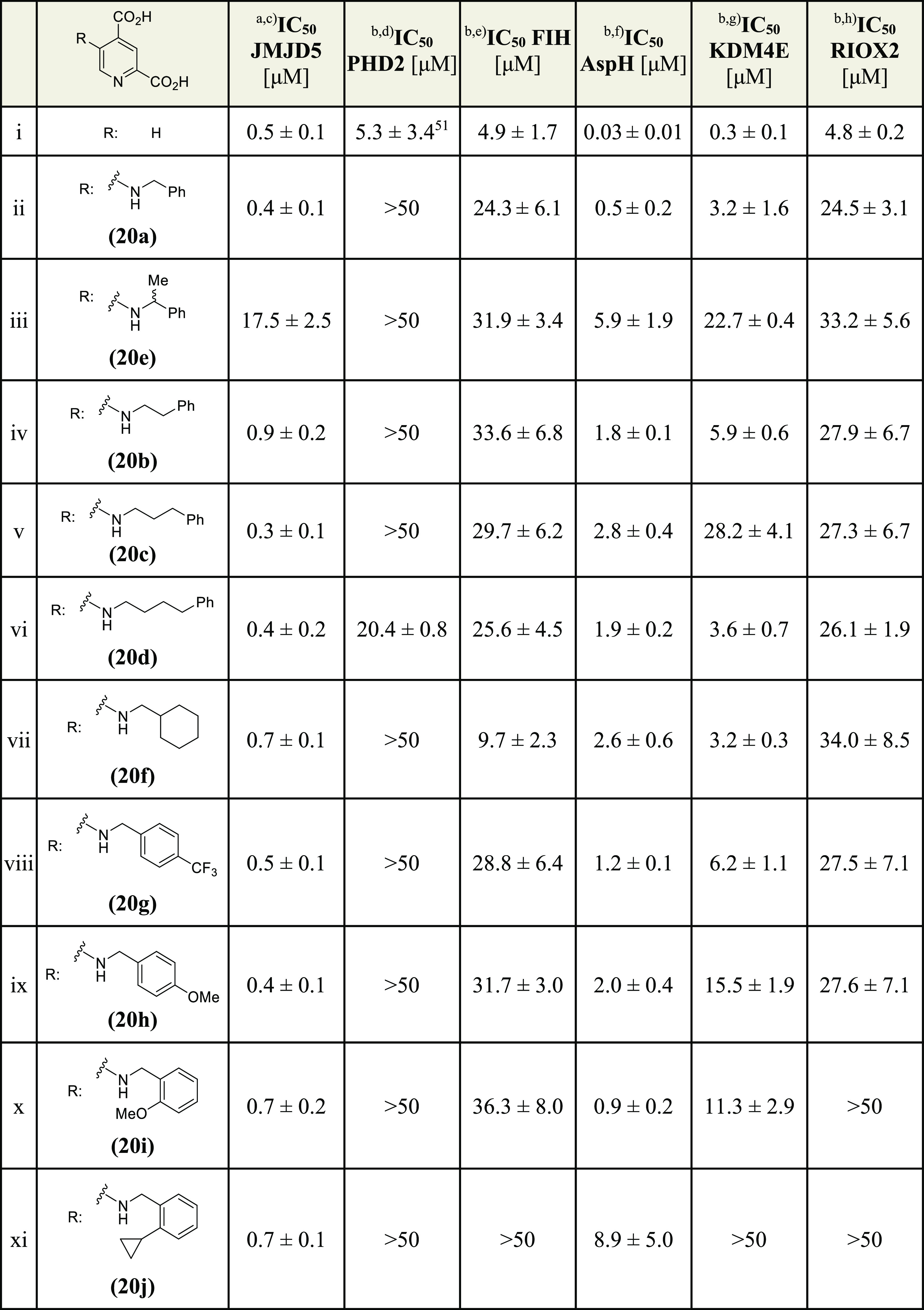
5-Aminoalkyl-Substituted 2,4-PDCA Derivatives Inhibit Human JMJD5 with Higher Selectivity Than 2,4-PDCA.

a Data are a mean of three independent runs (*n* = 3; mean ± SD), representative dose–response curves are shown in Figure 3.

b Data are a mean of two independent runs (*n* = 2; mean ± SD); SPE-MS inhibition assays were performed as described:

c JMJD5 (0.15 μM), 2OG (2.0 μM), Fe(II) (2.0 μM), LAA (100 μM), and RPS6_128–148_ (2.0 μM);^42^

d PHD2_181–426_ (0.15 μM) and 5.0 μM HIF-1α C-terminal oxygenase-dependent domain fragment (HIF-1α CODD, residues 558–574);^70^

e His_6_-FIH (0.15 μM) and 5.0 μM HIF-1α C-terminal transactivation domain fragment (HIF-1α CAD, residues 788–822);^70^

f His_6_-AspH_315–758_ (0.05 μM) and 1.0 μM of a thioether-linked cyclic peptide based on human coagulation factor X (hFX, residues 101–119; hFX-CP_101–119_);^45,46^

g KDM4E (0.15 μM) and 10.0 μM of a variant of a histone 3 fragment (H3_1–15_K9me3, residues 1–15);^72,73^

h His_6_-RIOX2_26–465_ (0.15 μM) and 5.0 μM of the RPL27A_31–49_ peptide.^44,71^

The inhibition results clearly indicate the potential utility of the C5 substitution of the 2,4-PDCA scaffold, considering the substantial loss of inhibition potency associated with the regioisomeric 2,4-PDCA derivatives, which bear identical substituents at the C3 position (Table 1). Analysis of the JMJD5 inhibition curves informed on the mechanism of JMJD5 inhibition in vitro, as their Hill slopes^69^ are near the theoretical value of −1, in accord with a 2OG-competitive binding mode (Figure 3).

**Figure 3:**
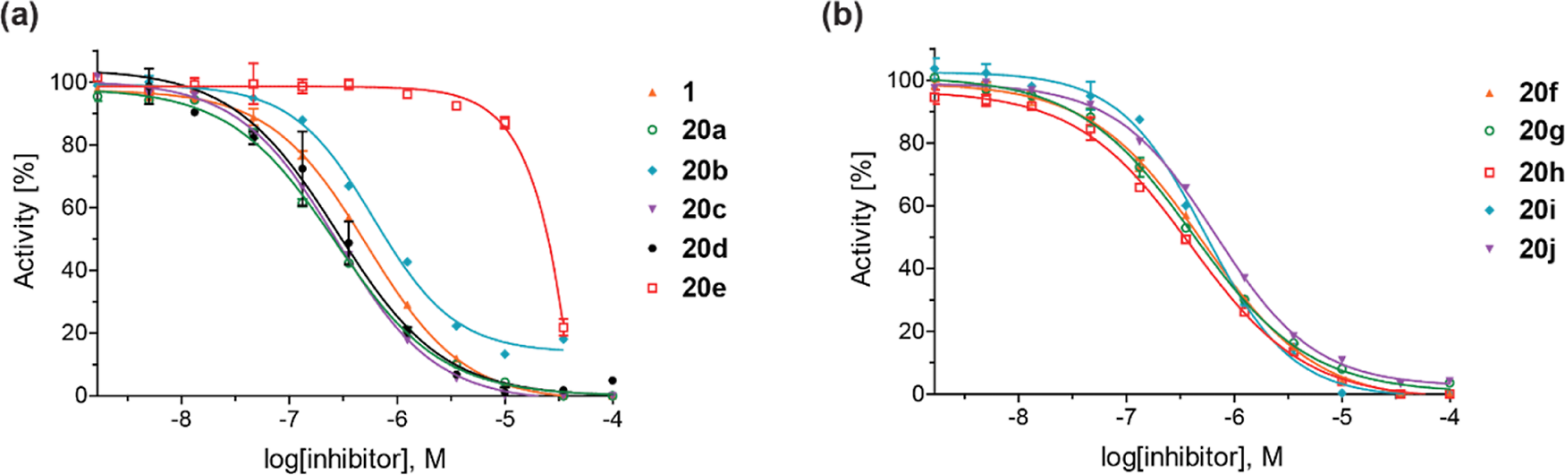
Representative dose–response curves of 5-aminoalkyl-substituted 2,4-PDCA derivatives used to determine IC_50_ values for JMJD5 inhibition. (a) **1** (orange triangles), **20a** (green circles), **20b** (cyan diamonds), **20c** (violet inverse triangles), **20d** (black circles), and **20e** (red boxes) and (**b**) **20f** (orange triangles), **20g** (green circles), **20h** (red boxes), **20i** (cyan diamonds), and **20j** (violet inverse triangles). Three dose–response curves, each composed of technical duplicates, were independently determined using reported SPE-MS JMJD5 inhibition assays.^42^

### 5-Aminoalkyl-substituted 2,4-PDCA Derivatives Show a Substantially Improved Selectivity Profile for JMJD5 Inhibition

The selectivity of the 5-aminoalkyl-substituted 2,4-PDCA derivatives for inhibiting JMJD5 was investigated using a representative set of isolated recombinant human 2OG oxygenases comprising different subclasses for which SPE-MS inhibition assays have been reported to ensure comparability of the results (Table 2). We investigated the inhibition of 2OG-dependent hydroxylases present in the cytosol, nucleus, and endoplasmic reticulum, i.e., HIF-α (PHD2), which is a validated drug target,^38−41^ FIH,^70^ ribosomal oxygenase 2 (RIOX2, Mina 53),^71^ AspH,^45,46^ and KDM4E (Supporting Information Figure S4).^72,73^

Initially, IC_50_-values for 2,4-PDCA (**1**) were determined as a benchmark for PHD2, FIH, RIOX2 (Mina 53), AspH, and KDM4E, in all cases using SPE-MS assays (Table 2, entry i). 2,4-PDCA (**1**) was most efficient in inhibiting AspH, which likely reflects the lower 2OG concentration employed in the AspH SPE-MS inhibition assays compared to the other 2OG oxygenase assays;^45^**1** inhibited AspH ∼10-fold more efficiently than JMJD5 and KDM4E and ∼100-fold more efficiently than PHD2, FIH, and RIOX2 (IC_50_ ∼ 0.03 μM; Table 2, entry i).

In general, an aminoalkyl substituent at the 2,4-PDCA C5 position reduced the potency of 2OG oxygenase inhibition, with the notable exception of JMJD5 which was, at least predominantly, inhibited with similar potency by the C5 substituted derivatives as by **1**. For example, 2,4-PDCA derivative **20c** inhibited JMJD5 with equal potency as **1** but inhibited AspH ∼100-fold less efficiently, resulting in a reversal of inhibition selectivity (Table 2, entry v); **20c** thus inhibits JMJD5 ∼10-fold more efficiently than AspH. Note, however, that the AspH and JMJD5 SPE-MS assays employ different enzyme, 2OG, and substrate concentrations, which complicates direct comparison of the inhibition results. Similarly, **20c** inhibits KDM4E ∼100-fold less efficiently than **1** and thus inhibits JMJD5 ∼100-fold more selectively than KDM4E. The selectivity of **20c** for JMJD5 versus PHD2, FIH, and RIOX2 inhibition increases by a factor of ∼6 to 10 with respect to **1**, being ∼100-fold (Table 2, entry v).

Interestingly, incrementally increasing the length of the alkyl chain that links the 5-amino group and the phenyl substituent from methylene (**20a**) via ethylene (**20b**) and propylene (**20c**) to butylene (**20d**) impacts on the selectivity observed for JMJD5 inhibition over AspH and KDM4E inhibition, which appeared to peak for the propylene derivative **20c** (Table 2, entry v). 5-Aminobenzyl-substituted 2,4-PDCA derivative **20a** was used to investigate the effect of substituents on the phenyl ring on the selectivity of JMJD5 inhibition, in particular with respect to AspH inhibition. Introducing an electron-withdrawing CF_3_ substituent para to the methylene linker of **20a** did not improve selectivity for JMJD5 versus AspH inhibition (Table 2, entry viii). Similarly, an electron-donating OMe substituent para or ortho to the methylene linker of **20a** did not improve the selectivity of JMJD5 versus AspH inhibition (Table 2, entries ix and x). Crystallographic analysis of the JMJD5:**20i** complex (see below) suggested that sterically more bulky substituents may be tolerated at the position ortho to the methylene linker. Hence, **20j**, which bears a cyclopropyl substituent ortho to the methylene linker, was synthesized; **20j** inhibits JMJD5 with similar potency as **1** (IC_50_ ∼ 0.7 μM; Table 2, entry xi). Remarkably, the selectivity of **20j** for JMJD5 inhibition was >70-fold for all the 2OG oxygenases investigated, except for AspH where the selectivity was >12-fold. Potentially, sterically bulkier substituents than cyclopropyl at the position ortho to the methylene linker of **20a** may result in a further increase of the selectivity for JMJD5 versus AspH inhibition.

### Crystallographic Analyses of JMJD5 Complexed with C5-Substituted 2,4-PDCA Derivatives

Crystallization studies were initiated to investigate the binding mode of the 5-aminoalkyl-substituted 2,4-PDCA derivatives with JMJD5 and thus to help enable SAR studies directed at further improving inhibitor selectivity and potency. JMJD5 crystal structures in complex with the C5-substituted 2,4-PDCA derivatives **20a**, **20d**, **20h**, **20i**, and **20j** were obtained by soaking the 2,4-PDCA derivatives into apo-crystals of N-terminally truncated JMJD5^35,36^ (JMJD5_183–416_; *P*2_1_2_1_2_1_ space group) in the presence of Mn(II), which replaces the natural cofactor Fe(II). The structures were solved by molecular replacement using a reported structure of JMJD5 in complex with the 2OG mimetic *N*-oxalylglycine (NOG) and the RPS6-derived peptide RPS6_129–144_ (JMJD5:NOG:RPS6_129–144_; PDB ID: 6F4P(35)) as a search model (1.6–2.4 Å resolution, Supporting Information Table S3). The JMJD5:**20a**, **20d**, **20h**, **20i**, and **20j** complex structures manifest similar overall JMJD5 folds, as observed in the reported JMJD5:2,4-PDCA (**1**) (PDB ID: 6I9L(36)) and JMJD5:2OG (PDB ID: 6F4N(35)) complex structures (Supporting Information Figures S5–S14).

**Figure 4:**
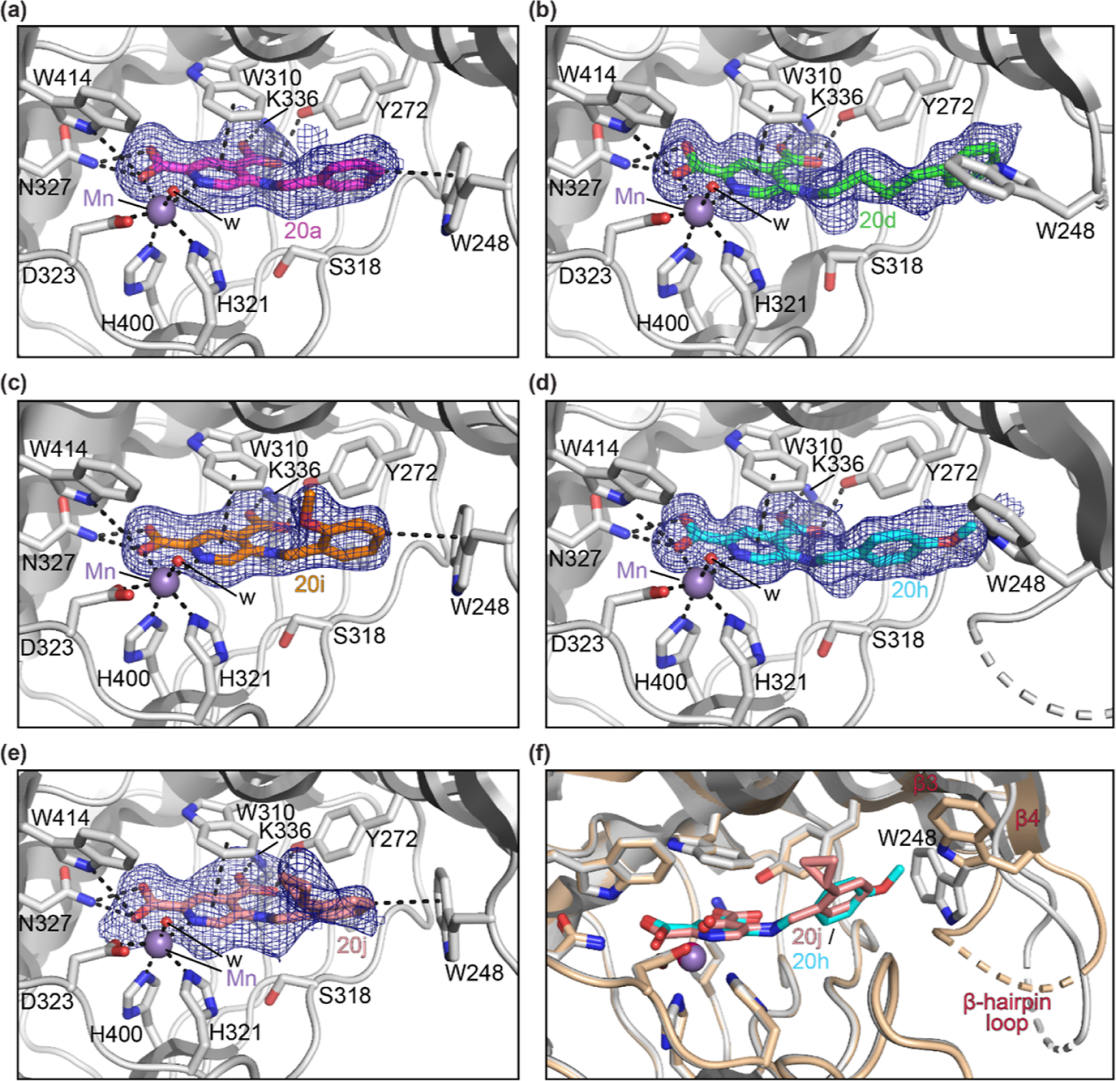
5-Aminoalkyl-substituted 2,4-PDCA derivatives bind to the JMJD5 active site. Color code: gray: JMJD5; lavender blue: Mn; red: oxygen; and blue: nitrogen. w: water. Representative OMIT electron density map (*mF*_o_ – *DF*_c_) contoured to 2.5σ around (a) **20a** (purple: carbon backbone of **20a**) in the JMJD5:**20a** complex (PDB ID: 7DYV), (b) **20d** (green: carbon backbone of **20d**) in the JMJD5:**20d** complex (PDB ID: 7DYU), (c) **20i** (orange: carbon backbone of **20i**) in the JMJD5:**20i** complex (PDB ID: 7DYW), (d) **20h** (contour level: 3.0σ; cyan: carbon backbone of **20h**) in the JMJD5: **20h** complex (PDB ID: 7DYX), and (e) **20j** (salmon: carbon backbone of **20j**) in the JMJD5:**20j** complex (PDB ID: 7DYT). (f) Superimposition of active site views of the JMJD5:**20h** (ochre: JMJD5; PDB ID: 7DYX) and JMJD5:**20j** (gray: JMJD5; PDB ID: 7DYT) complex structures highlights the conformational flexibility of the W248-bearing loop (G240 to W248), while other JMJD5 regions have an overall similar fold (Cα RMSD 0.149 Å; Supporting Information Figure S15). The conformation of the W248 side chain reflects its ability to engage in a σ–π interaction with the para C–H of the **20j** phenyl group and the W248 indole group.

Electron density corresponding to **20a**, **20d**, **20h**, **20i**, or **20j** was clearly observed at the JMJD5 active site in the respective JMJD5:2,4-PDCA derivative complex structures (Figure 4a–e). In all of the five structures, the core 2,4-PDCA scaffold of **20a**, **20d**, **20h**, **20i**, or **20j** binds to the JMJD5 active site in an analogous manner to 2,4-PDCA (**1**) itself, i.e., the heteroaromatic pyridine unit is positioned to interact with the indole side chain of W310 via π-stacking, the C4 carboxylate of the 2,4-PDCA cores is positioned to interact with the side chain amino group of K366 and the side chain hydroxyl group of Y272, and the C2 carboxylate is positioned to interact with the side chain amide group of N327 and the indole NH group of W414. The C2 carboxylate is positioned to chelate the active site metal, together with the pyridine nitrogen atom (Supporting Information Figures S5–S9). Note that in all five structures, the side chain of S318, which is adjacent to the 2,4-PDCA C5 position, was observed in a single conformation in which it is oriented away from the C5 substituents of **20a**, **20d**, **20h**, **20i**, and **20j** (Figure 4a–e). This observation contrasts with the reported JMJD5:**1** (PDB ID: 6I9L(36)) complex structure (Figure 1d), where the side chain of S318 was observed in two conformations. The mobility of S318 and other active site-adjacent elements likely reflects roles in induced fit during JMJD5 substrate binding (Figure 4f), as observed for other 2OG oxygenases, both with substrates and, at least, some inhibitors.^74,75^

The JMJD5 structures in complex with the C5-substituted 2,4-PDCA derivatives reveal that these bind to the 2OG binding pocket and that their C2 and C4 carboxylate groups interact with JMJD5 in a similar manner as observed for the C1 and C5 carboxylate groups of 2OG in a reported JMJD5:2OG complex structure (PDB ID: 6F4N;^35^Supporting Information Figures S10–S14), in accord with similar interactions observed for the C2 and C4 carboxylate groups of **1** in the reported JMJD5:**1** complex structure.^36^ Thus, the 5-aminoalkyl-substituted 2,4-PDCA derivatives inhibit JMJD5, in part, via competing with 2OG for binding.

The phenyl group of the 5-aminobenzyl substituted 2,4-PDCA derivatives **20a**, **20i**, and **20j** adopts a conformation in complex with JMJD5 in which its para C–H is positioned to interact with the indole group of W248 via a σ–π interaction (Figure 4). By contrast, the side chain of W248 occupies a different conformation in the JMJD5:**20d** and **20h** complex structures, i.e., it faces away from the phenyl group of the C5 substituent of **20d**/**20h** (Figure 4f). This is likely because σ–π interactions similar to those observed in the JMJD5:**20a**, **20i**, and **20j** complex structures are unfeasible, either because the phenyl group para C–H is substituted for a para C–OMe (as in **20h**) that cannot engage in σ–π interactions, or because the distance/orientation between the phenyl group of the ligand’s C5 substituent and the indole group of W248 does not favor productive σ–π interactions (as in **20d**). The latter scenario potentially reflects the presence of a butylene unit connecting the phenyl group with the C5 amino group in **20d** compared to the shorter methylene units in **20a**, **20i**, and **20j**. The observation that except for **20e**, all of the tested 5-aminoalkyl-substituted 2,4-PDCA derivatives inhibit isolated recombinant JMJD5 with similar efficiency (Table 2) indicates that the σ–π interaction of the ligand’s phenyl group with the indole group of W248 is not essential for potent inhibition. The results also highlight the conformational flexibility of the W248-bearing loop (G240 to W248), which is part of the β-hairpin linking β3 and β4 (T234 to T254); electron density was not observed for some of the residues in this loop, suggesting that it can occupy multiple conformations, in accord with its proposed role in modulating substrate binding.^35^

**Figure 5:**
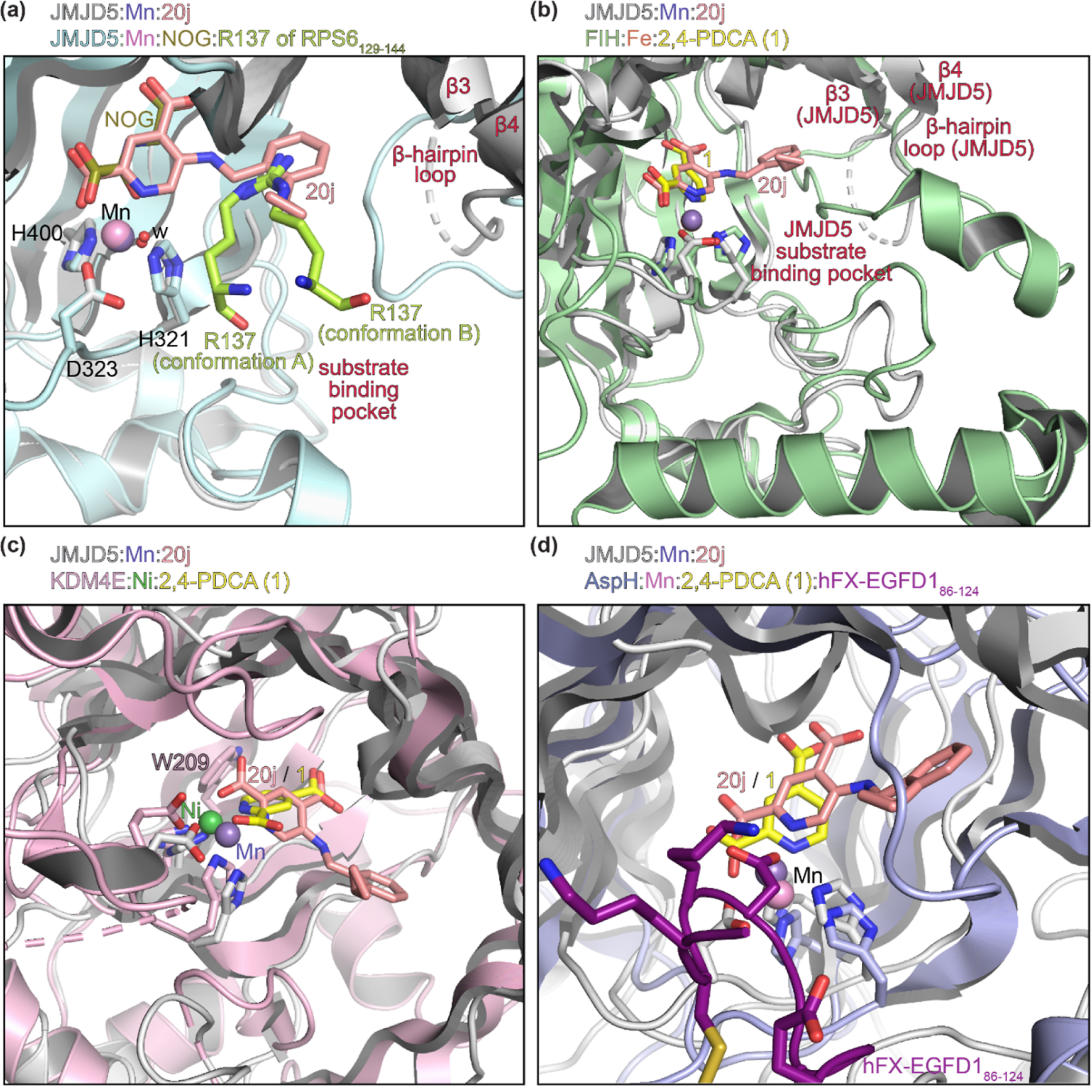
C5 substituent of 2,4-PDCA derivatives projects into the substrate-binding pocket of JMJD5 but not into the substrate-binding pockets of FIH, KDM4E, and AspH. Superimposition of the JMJD5:**20j** complex structure (PDB ID: 7DYX) with the reported (a) JMJD5:NOG:RPS6_129–144_ (pale blue: JMJD5; pink: Mn; green: carbon backbone of R137 of RPS6_129–144_; PDB ID: 6F4P(35)), (b) FIH:**1** (pale green: FIH; brown: Fe; PDB ID: 2W0X(76)), (c) KDM4E:**1** (pale pink: KDM4E; green: Ni; PDB ID: 2W2I(77)), and (d) AspH:**1**:hFX-EGFD_86–124_ (pale lavender: AspH; pink: Mn; violet: carbon backbone of hFX-EGFD_86–124_; PDB ID: 5JTC(45)) complex structures. Color code: gray: JMJD5; lavender blue: Mn; salmon: carbon backbone of 5-((2-cyclopropylbenzyl)amino)pyridine-2,4-dicarboxylic acid (**20j**); yellow: carbon backbone of 2,4-PDCA (**1**); red: oxygen; blue: nitrogen. w: water.

As anticipated based on the analysis of the reported JMJD5:**1** complex structure (PDB ID: 6I9L),^36^ the 2,4-PDCA C5 position is adjacent to the relatively wide substrate-binding pocket (Figure 4), which is spacious enough to accommodate sterically bulky substituents as present in 2,4-PDCA derivatives **20a**, **20d**, **20h**, **20i**, and **20j**. Superimposition of the JMJD5:**20j** complex structure with that of the reported JMJD5:NOG:RPS6_129–144_ complex structure (PDB ID: 6F4P(35)) reveals an overall similar fold of JMJD5 in the inhibitor and substrate-bound structures (Cα RMSD 0.263 Å; Supporting Information Figure S16), with the exception of the W248-bearing loop (G240 to W248) (Figure 5a).

Superimposition of the JMJD5:**20j** and the reported JMJD5:NOG:RPS6_129–144_ (PDB ID: 6F4P(35)) complex structures reveals that the phenyl ring of **20j** binds in the same position as the guanidyl group of R137 in the JMJD5:NOG:RPS6_129–144_ complex structure (Figure 5a). Note that JMJD5 catalyzes the C3 hydroxylation of R137 of RPS6_129–144_ and that R137 was observed in both apparently productive (A, Figure 5a) and non-productive (B, Figure 5a) conformations in a JMJD5:NOG:RPS6_129–144_ complex structure.^35^ The superimposition also indicates that the cyclopropyl group of **20j** directly extends further into the substrate-binding site, suggesting that substituting the cyclopropyl group of **20j** for sterically bulkier groups may enhance inhibitor selectivity and/or potency (and may be useful to modulate the physicochemical properties of **20j**) (Figure 5a). Note that the superimposition does not inform on the reason(s) why the potency of 2,4-PDCA derivative **20e**, which bears a methyl substituent at the benzylic position α to the amine, is substantially lower than the potencies of the other tested C5-substituted 2,4-PDCA derivatives (Table 2).

To inform on reasons for the observed selectivity of **20j** to inhibit JMJD5 over other tested human 2OG oxygenases (Table 2), the JMJD5:**20j** complex structure was superimposed with those of the reported FIH:**1** (PDB ID: 2W0X(76)), KDM4E:**1** (PDB ID: 2W2I(77)), and AspH:**1**:hFX-EGFD_86–124_ (PDB ID: 5JTC(45)) complex structures (Figure 5). The comparisons imply that only the substrate-binding pocket of JMJD5 provides sufficient space adjacent to the 2,4-PDCA C5 position to accommodate relatively bulky substituents (Figure 5).

Despite the overall similar folds of JMJD5 and FIH and the identical binding orientations of **20j** and **1** in the JMJD5:**20j** and the reported FIH:**1** (PDB ID: 2W0X(76)) complex structures (Cα RMSD 1.252 Å; Supporting Information Figure S17), the shape and accessibility of the JMJD5 and FIH substrate-binding pockets differ substantially. The W248-bearing loop near the substrate-binding pocket of JMJD5, which is part of the β-hairpin motif involving β3 and β4 (T234 to T254), comprises 9 residues (i.e., G240 to W248). By contrast, the corresponding loop in FIH comprises 25 residues (i.e., A95 to N119) and extends toward the two C-terminal α-helices, likely resulting in a relatively smaller substrate-binding pocket in this region (Figure 5b); indeed, comparison of the reported JMJD5:NOG:RPS6_129–144_ (PDB ID: 6F4P(35)) complex structure with a reported FIH structure in complex with a HIF-1α-derived substrate peptide reveals that the conformation of the HIF-1α substrate in complex with FIH differs from that of the RPS6 substrate in complex with JMJD5 (Supporting Information Figure S17).

The C5 substituent of the 2,4-PDCA derivatives is close to the β3-β4 linking loop in the FIH structure; thus, it is likely that increasing the steric bulk of the C5 substituent will reduce inhibition potency, in accord with the observation that **20j**, which has a sterically bulky *ortho*-cyclopropyl phenyl substituent, is less potent in inhibiting FIH than the other tested C5-substituted 2,4-PDCA derivatives (Table 2). Note that W310, the indole group of which is positioned in the JMJD5 active site to interact with the pyridine of the 2,4-PDCA derivatives via π-stacking, is substituted for a leucine residue in FIH (i.e., L188). The lack of the stabilizing π-stacking interaction in FIH may, at least in part, explain the observed overall reduction in the potency of **1** and its derivatives in inhibiting FIH compared to JMJD5 (Table 2).

Superimposition of the JMJD5:**20j** and the reported KDM4E:**1** (PDB ID: 2W2I(77)) complex structures indicates a different binding mode of 2,4-PDCA (**1**) (Cα RMSD 1.104 Å; Supporting Information Figure S18), i.e., the 2,4-PDCA C5 substituent projects outside of the JMJD5 β-barrel into the substrate-binding pocket, whereas it projects inside the KDM4E β-barrel and thus away from the assigned substrate-binding pocket in the KDM4E complex structure (Figure 5c). This observation suggests that increasing the steric bulk of the 2,4-PDCA C5 substituent will likely result in reduced inhibition potency due to unfavorable interactions with side chains of KDM4E residues that project inside the β-barrel in proximity of the 2,4-PDCA derivatives, e.g., W209 (Figure 5c), as indeed observed (Table 2).

Superimposition of the JMJD5:**20j** and the reported AspH:**1**:hFX-EGFD_86–124_ (hFX-EGFD_86–124_^48,80^ is based on the EGFD of the AspH substrate human factor X (hFX);^78,79^ PDB ID: 5JTC(45)) complex structures reveals that the 2,4-PDCA C5 substituent projects away from the AspH substrate-binding pocket and projects toward β-strands, which form the AspH β-barrel (Figure 5d and Supporting Information Figure S18). Thus, consistent with the inhibition results (Table 2), the structural analyses suggest that 5-aminoalkyl-substituted 2,4-PDCA derivatives with sterically bulky C5 substituents will not efficiently bind to AspH due to steric interactions with the side chains of residues that project inside the β-barrel in proximity of the 2,4-PDCA C5 position. Co-crystallization of 5-aminoalkyl-substituted 2,4-PDCA derivatives with AspH has not afforded single crystals with ligand bound, potentially due to relatively weak binding.

### Cell-Based Studies

The dimethyl ester prodrug form of 2,4-PDCA has been extensively used as a broad-spectrum inhibitor to investigate the functions of 2OG oxygenases in cell-based studies.^67,68^ Because the diester prodrugs are likely to more efficiently enter cells where they are transformed into the active inhibitor by esterases, we employed the dimethyl esters of the most potent and selective JMJD5 inhibitors **20i** and **20j** (Table 2), i.e., **19i** and **19j**, to investigate their effects on cells.

**Figure 6:**
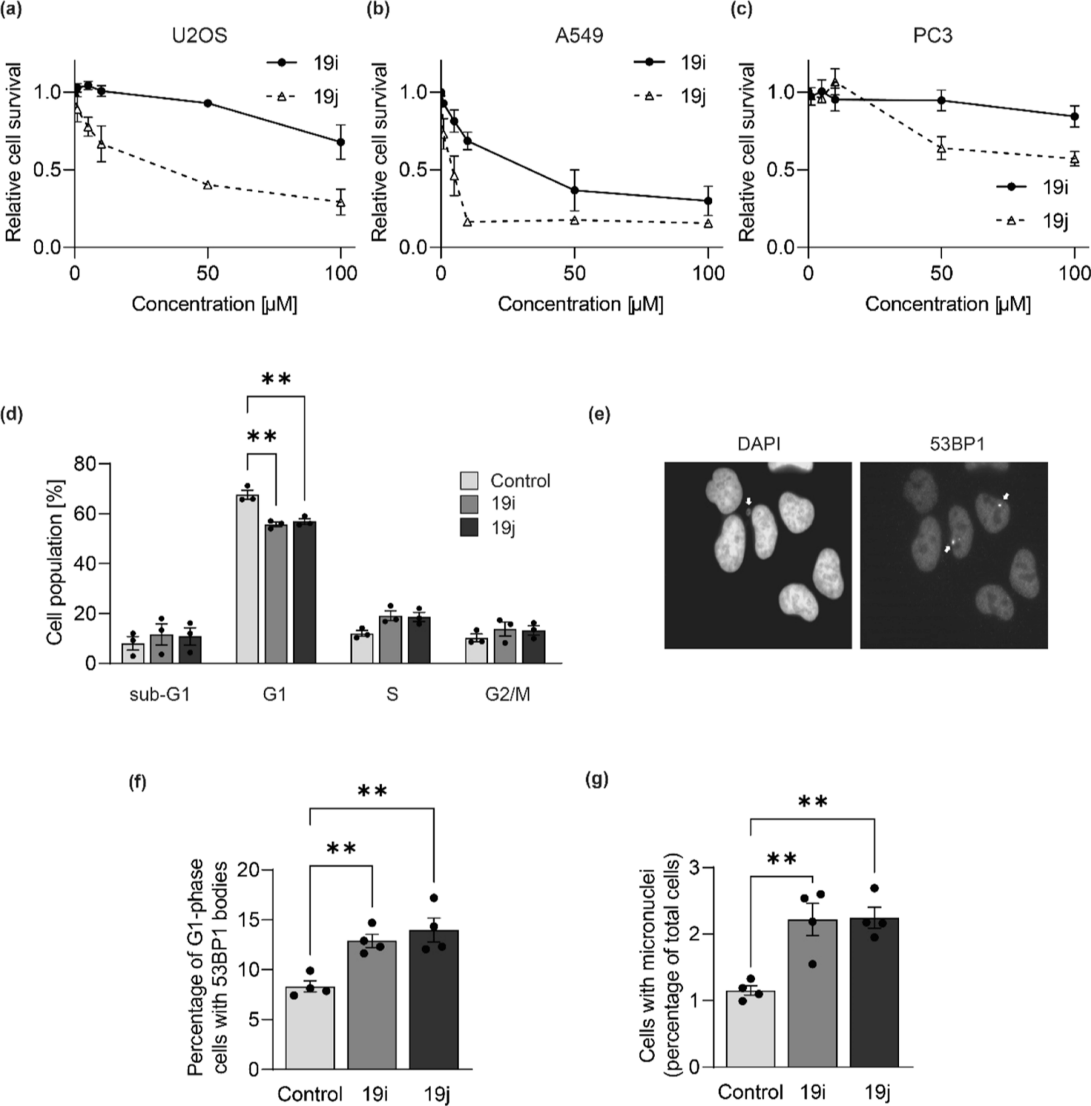
5-Aminoalkyl-substituted 2,4-PDCA diester derivatives **19i** and **19j** cause reduced viability, cell cycle disturbances, and replication stress in tumor cells. (a) U2OS bone osteosarcoma, (b) A549 lung adenocarcinoma, and (c) PC3 prostate adenocarcinoma cells were treated with the indicated doses of **19i** and **19j** (1.0, 5.0, 10, 50, and 100 μM) for 72 h and then tested for viability using the 3-(4,5-dimethylthiazol-2-yl)-5-(3-carboxymethoxyphenyl)-2-(4-sulfophenyl)-2*H*-tetrazolium (MTS) assay. A549 cells were treated with **19i** or **19j** (1.0 μM) for 24 h, then stained with (d) propidium iodide and analyzed by FACS or (e) DAPI staining for nuclei (left) and anti-53BP1 (for 53BP1 bodies, right). Cells were counted for 53BP1 bodies (f) and micronuclei (g). Data represent mean ± SEM from three (a–d) or four (f–g) independent replicates. For 53BP1 bodies, a minimum of 300 cells were counted per sample. For micronuclei, a minimum of 500 cells were counted per sample. Statistical analyses used one-way ANOVA with Tukey’s post hoc test [with *p*-value ≤ 0.01 (**)].

A biochemical readout of JMJD5 arginine residue hydroxylase activity in cells is not available; we thus focused our studies on biological readouts previously linked to JMJD5 function in order to investigate cellular responses resulting from JMJD5 inhibitor treatment. JMJD5 loss of function by RNA interference is reported to affect tumor cell proliferation and viability;^16,18,20,21^ we therefore studied the effects of **19i** and **19j** on the growth of three different tumor cell lines. Whereas **19i** did not significantly affect U2OS bone osteosarcoma or PC3 prostate adenocarcinoma cells, it substantially reduced the growth of A549 lung adenocarcinoma cells, with a half-maximum cytotoxicity concentration (CC_50_ value) of 10.7 μM (Figure 6a–c). Consistent with the in vitro data, **19j** was more potent than **19i**, with CC_50_ values of 10.0 and 2.1 μM in U2OS and A549 cells, respectively, which was further confirmed using an orthogonal approach (Supporting Information Figure S19).

Because JMJD5 RNA interference has been associated with disturbances in the cell cycle,^1,2,81,82^ we measured phase distribution by flow cytometry in A549 cells (Figure 6d). Treatment with **19i** or **19j** for 24 h caused a reduction in G1-phase cells, with a corresponding increase in S- and G2/M-phase cells. Sub-G1-phase cell numbers were also increased (Figure 6d), an observation indicative of cell death. To explore the potential causes of the reduced viability and cell cycle alterations, we studied JMJD5-related biological processes. We have recently reported that JMJD5 loss of function induces replication stress,^14^ which is defined as a perturbation that slows or stalls DNA replication.^83^ Unresolved replication stress can be measured by an increase in G1-phase 53BP1 “bodies” and micronuclei (Figure 6e), which are extranuclear DNA bodies that result from DNA cleavage and genome instability following replication stress. Consistent with a role for JMJD5 in replication stress, treating A549 cells with **19i** or **19j** (1.0 μM) for 24 h significantly induced both micronuclei (Figure 6f) and 53BP1 bodies (Figure 6g), indicating reduced replication fidelity and increased genome instability. Although further work is required to define the role of JMJD5 in their manifestation, these observations are consistent with inhibition of JMJD5 by **19i** and **19j** in cells.

## Discussion and Conclusions

Both from physiological and disease perspectives, JMJD5 is an interesting member of the 60–70 assigned human 2OG oxygenases. Genetic studies show it has important roles, including in development, genome stability, and circadian rhythm;^2,5,6,11−14^ JMJD5 is also linked to cancer, though in a context-dependent manner.^17−23^ Reports on reactions catalyzed by JMJD5 are also, at least in some aspects, contradictory—JMJD5 is reported to have histone protease activity,^28−30^ KDM activity,^24−27^ and, as we have shown, at least in isolated form, arginine hydroxylase activity.^35,36,42^ To investigate the biological roles of JMJD5 and evaluate its potential as a cancer target, small-molecule inhibitors are of potential value, as shown by work on other 2OG oxygenases, including the HIF-α prolyl residue hydroxylases.^38−41^ However, only a few studies on small-molecule JMJD5 inhibitors are reported,^36,42^ likely, at least in part, due to a lack of robust assays to monitor JMJD5 catalysis in vitro, which has only recently been addressed by the development of a SPE-MS-based inhibition assay.^42^

We applied a structure-guided approach with the aim of developing potent and selective JMJD5 inhibitors following analysis of a crystal structure of the relatively broad-spectrum 2OG oxygenase inhibitor 2,4-PDCA (**1**) in complex with JMJD5.^36^ Initially, we identified C3 substitution of **1** as a possible means to obtain potent and selective JMJD5 inhibitors. Although some of the initially tested reported C3-substituted 2,4-PDCA derivatives^44,51^ showed an improved selectivity profile for JMJD5 inhibition over inhibition of other human 2OG oxygenases, in general, they lacked sufficient potency (Table 1).

The SAR results of the 2,4-PDCA derivatives coupled with the structural analysis of the JMJD5:**1** complex structure suggested that 2,4-PDCA derivatives with C5 substituents may inhibit JMJD5. Although the available JMJD5 crystal structures implied that movement of the S318 side chain would be required in order to accommodate the C5 substituents of 2,4-PDCA derivatives, such induced fit has precedent in studies on the binding of inhibitors to other 2OG oxygenases.^74,75^ Pleasingly, by contrast with the results from the 2,4-PDCA C3 derivatives, the corresponding isomeric C5 aminoalkyl-substituted 2,4-PDCA derivatives showed both a substantially improved potency and selectivity (in many cases > 100-fold) for JMJD5 inhibition, in particular inhibitors **20c** and **20j** (Table 2). The gain in selectivity for JMJD5 inhibition versus AspH and KDM4E inhibition associated with the introduction of aminoalkyl-substituents at the 2,4-PDCA C5 position is remarkable, considering the potency with which **1** inhibits both KDM4E and AspH, that **1** the latter of which it was also shown to bind tightly.^80^

Crystallographic studies with 2,4-PDCA derivative **20j** indicate that the JMJD5 substrate-binding pocket has sufficient space to accommodate a relatively sterically bulky cyclopropyl substituent at the phenyl ortho position of the C5 substituent. By contrast, the reported KDM4E:**1**^50^ and AspH:**1**^51^ complex structures imply a lack of sufficiently spacious pockets in proximity of the potential 2,4-PDCA C5 position (Figure 5). These observations suggest that increasing the steric bulk at the phenyl ortho position will likely result in further improvement of the inhibition selectivity for JMJD5 via additional interference with substrate binding. The crystallographic analyses also imply that there is potential scope for substituents at the 2,4-PDCA C6 position to enable selective inhibition. Note, however, that targeting other pockets in proximity to the JMJD5 active site, e.g., the binding site of a methylated arginine,^29^ may be a complementary method to develop potent and selective JMJD5 inhibitors.

The SAR studies highlight how appropriate substituents can drastically alter the inhibition efficiency and selectivity of relatively broad-spectrum 2OG oxygenase inhibitors such as 2,4-PDCA (**1**). Structural modification of broad-spectrum 2OG oxygenase inhibitors has previously enabled the identification of small-molecule inhibitors with improved selectivity profiles, e.g., the substitution of the glycine unit of the broad-spectrum 2OG oxygenase inhibitor *N*-oxalylglycine (NOG) for a d-phenylalanine unit afforded an apparently selective FIH inhibitor, i.e., *N*-oxalyl-d-phenylalanine (NOFD).^84^ The *N*-oxalyl amino acid derivatives require application as prodrugs in order to manifest activity in cells. This is likely also the case for our JMJD5 inhibitors, though studies with other 2OG oxygenases have shown it should be possible to replace at least one of the two carboxylates and maintain potent inhibitory activity,^85,86^ which is the subject of ongoing work.

Considering that pyridine carboxylates and related compounds are being actively pursued for the development of clinically useful 2OG oxygenase inhibitors,^37,85,86^ there is likely scope for further optimization of the C5 aminoalkyl-substituted 2,4-PDCA derivatives **20c** and **20j** as JMJD5 inhibitors for use in cellular functional assignment and animal studies. Nonetheless, cell-based studies with prodrug derivatives of potent JMJD5 inhibitors (i.e., **19i** and **19j**) manifest similar cellular phenotypes as those observed in genetic studies, including the induction of replication stress (Figure 6).^14^ Ongoing biological work is focusing on developing optimized JMJD5 inhibitors and on using **19i** and **19j** to investigate connections between JMJD5 catalysis and its cellular and physiological roles.

## Experimental Section

The syntheses and characterization of the C5-substituted JMJD5 inhibitors used in this work are described in the associated Supporting Information. All compounds are ≥95% pure, as determined by NMR and HPLC analyses unless stated otherwise; NMR spectra and HPLC traces are shown for all lead compounds in the associated Supporting Information.

### Production and Purification of Human Recombinant 2OG Oxygenases

Recombinant human N-terminally His_6_-thioredoxin-tagged JMJD5,^42^ PHD2_181–426_,^70^ N-terminally His_6_-tagged FIH,^70^ N-terminally His_6_-tagged KDM4E,^72,73^ N-terminally His_6_-tagged RIOX2_26–465_,^87,88^ and N-terminally His_6_-tagged AspH_315–758_^48,80^ were prepared according to established procedures in *Escherichia coli* cells. The enzymes were >95% pure as determined by SDS-PAGE and MS analyses and had the anticipated masses; fresh aliquots were used for all inhibition studies. For crystallizations, N-terminally truncated JMJD5_183–416_ was prepared as reported.^35,36^

### Substrates for SPE-MS Inhibition Assays

Synthetic peptides with sequences based on reported human 2OG oxygenase substrates were used in SPE-MS inhibition assays: RPS6, residues 128–148, for JMJD5;^42^ HIF-α CODD, residues 558–574, for PHD2;^70^ HIF-α CAD, residues 788–822, for FIH;^70^ H3_1-15_K9me3, histone 3 (H3), residues 1–15 with K9 of H3-bearing three methyl groups at the *N*^ε^-position, K4 of H3 substituted by an alanine residue, and K14 of H3 by an isoleucine residue,^73^ for KDM4E; and RPL27A, residues 31–49, for RIOX2 (Mina53).^44^ All peptides were prepared as C-terminal amides by GL Biochem (Shanghai) Ltd. A synthetic thioether-linked cyclic peptide, hFX-CP_101–119_,^48^ which was designed based on the reported cellular AspH substrate human coagulation factor X (hFX, residues 101–119; C101 was substituted for a d-alanine residue and C112 for a serine residue),^79^ was used as AspH substrate; it was prepared with a C-terminal amide according to literature protocols.^80^

### SPE-MS Inhibition Assays

Cosubstrate/cofactor stock solutions [l-ascorbic acid/LAA: 50 mM in MQ-grade water; 2-oxoglutarate/2OG: 10 mM in MQ-grade water; ammonium iron(II) sulfate hexahydrate/FAS/(NH_4_)_2_Fe(SO_4_)_2_·6H_2_O: 400 mM in 20 mM HCl diluted to 1 mM in MQ-grade water] were freshly prepared from commercial solids (Sigma-Aldrich) on the day the assays were performed. The JMJD5,^42^ PHD2,^70^ FIH,^70^ KDM4E,^72,73^ RIOX2,^44^ and AspH^45^ SPE-MS inhibition assays were performed as described in the cited literature, using isolated recombinant human enzymes (His_6_-thioredoxin-JMJD5,^42^ His_6_-PHD2_181–426_,^70^ His_6_-FIH,^70^ His_6_-KDM4E,^72,73^ His_6_-RIOX2_26–465_,^87,88^ and His_6_-AspH_315–758_^48,80^) and synthetic peptide substrates (RPS6_128–148_ for JMJD5;^42^ HIF-α CODD_558–574_ for PHD2;^70^ HIF-α CAD_788–822_ for FIH;^70^ H3_1–15_K9me3 for KDM4E;^73^ RPL27A_31–49_ for RIOX2;^44,71^ and hFX-CP_101–119_ for AspH;^45,48^ see above). For JMJD5, PHD2, FIH, RIOX2, and AspH, substrate hydroxylation was monitored (+16 Da mass shift), while for the KDM4E substrate, demethylation was monitored (−14 Da mass shift) by SPE-MS.

### Crystallography

N-terminally truncated His_6_-tagged JMJD5_183–416_ (25 mg/mL) in buffer (50 mM HEPES, pH 7.5, 200 mM NaCl, and 5%_v/v_ glycerol) containing MnCl_2_ (1 mM) was mixed with a precipitant solution (100 mM HEPES buffer, pH 7.5, 200 mM magnesium chloride hexahydrate, and 25%_w/v_ PEG 3350) to give ratios of 2:1, 1:1, or 1:2 sample/precipitant. Crystallizations were set up in 96-well, 3-subwell, low-profile intelliplates (Art Robbins Instruments) using a Phoenix RE liquid dispensing robot (Art Robbins Instruments). Crystals were grown using the sitting drop vapor diffusion method at 20 °C, then soaked with inhibitor solution [1 μL of the precipitant solution was soaked with inhibitor (50 mM in DMSO) to give a final concentration of 5 mM inhibitor]. The soaked crystals were incubated for 6 h at 20 °C, then cryo-protected in the reservoir solution supplemented with 20%_v/v_ glycerol, and flash cooled using liquid N_2_. Diffraction data sets were collected at 100 K on beamlines I03 and I24 at the Diamond Light Source (DLS) and on beamlines ID30A-1 at the European Synchrotron Radiation Facility (ESRF). Data were indexed, integrated, and scaled using the XDS^89^ and autoPROC/STARANISO^90,91^ strategy of the beamline auto-processing pipeline (Supporting Information Table S1).

Initial phasing was performed by molecular replacement with the AutoMR (PHASER^92^) subroutine in PHENIX^93^ using the JMJD5:NOG:RPS6_129-144_ structure (PDB ID: 6F4P(35)) as a search model. The structural models were improved by iterative cycles of manual rebuilding in COOT^94^ and crystallographic refinement in phenix.refine^95^ (Supporting Information Table S1).

Crystallographic data for N-terminally His_6_-tagged JMJD5_183–416_ complexed to Mn, and the 5-aminoalkyl-substituted 2,4-PDCA derivatives [5-(benzylamino)pyridine-2,4-dicarboxylic acid, **20a**; 5-((4-phenylbutyl)amino)pyridine-2,4-dicarboxylic acid, **20d**; 5-((4-methoxybenzyl)amino)pyridine-2,4-dicarboxylic acid, **20h**; 5-((2-methoxybenzyl)amino)pyridine-2,4-dicarboxylic acid, **20i**; and 5-((2-cyclopropylbenzyl)amino)pyridine-2,4-dicarboxylic acid, **20j**] are deposited in the protein data bank with PDB accession codes: 7DYV (JMJD5:**20a**), 7DYU (JMJD5:**20d**), 7DYT (JMJD5:**20h**), 7DYW (JMJD5:**20i**), and 7DYX (JMJD5:**20j**). PyMOL^96^ was used for the generation of graphical representations; polder omit maps were calculated using Polder Maps^97^ in PHENIX.^93^

### Cell Culture

A549 and U2OS cells were cultured in Dulbecco’s modified Eagle’s medium with 1%_v/v_ penicillin/streptomycin (P/S) and 10%_v/v_ fetal bovine serum (FBS) and maintained at 37 °C in a humidified 5% CO_2_ incubator. PC3 cells were cultured in RPMI 1640 medium supplemented with 10%_v/v_ FBS, 2 mM l-glutamine, and 1%_v/v_ P/S.

### Cell Proliferation Assays

Cell proliferation/viability was measured using MTS (CellTiter 96 Aqueous One 667 Solution Cell Proliferation Assay, Promega) and CyQUANT (Thermo Fisher) assays. CellTiter 96 Aqueous MTS reagent (Promega) and phenazine methosulfate (Sigma-Aldrich) were dissolved in phosphate-buffered saline (PBS) according to the manufacturer’s protocol. The MTS working reagent was made according to the manufacturer’s protocol and added to each well containing the cells. Plates were incubated for 1 h at 37 °C under 5% CO_2_, protected from light, and then the absorbance was measured at 490 nm using an EnSpire Multimode plate reader (PerkinElmer). For the cyQUANT assay, the reagents were prepared according to the manufacturer’s protocol and added to the cells. The cells were incubated for 1 h at 37 °C under 5% CO_2_, protected from light; fluorescence was then measured with excitation at ∼485 nm and emission detection at ∼530 nm using an EnSpire Multimode plate reader.

### Immunofluorescence Staining

Cells on coverslips were pre-extracted on ice for 5 min using a buffer containing 20 mM NaCl, 3 mM MgCl_2_, 300 mM sucrose, 10 mM PIPES, and 0.5%_v/v_ Triton X-100 and fixed using 4%_v/v_ paraformaldehyde (PFA) in PBS for 15 min at ambient temperature. Cells were then permeabilized in 0.1%_v/v_ Triton X-100 in PBS at ambient temperature for 10 min and blocked in 1%_w/v_ bovine serum albumin (BSA) in PBS for 1 h at ambient temperature. Coverslips were incubated with anti-53BP1 antibody (Bio-Techne, NB100-904V) diluted in 1%_w/v_ BSA in PBS at ambient temperature for 1 h in the dark. Coverslips were then washed three times with cold 1%_w/v_ BSA in PBS following incubation for 1 h with secondary antibodies (anti-mouse 555 nm A31570 or anti-rabbit 488 nm A11070, Thermo Fisher). The coverslips were washed three times with cold PBS and incubated for 10 min with 4′,6-diamidino-2-phenylindole (DAPI) (Invitrogen) to stain for nuclei. Prolong Gold Antifade Reagent (Cell Signaling Technology) was used to mount the coverslips onto microscope slides. Images were taken using 40× objective on a Leica DM6000 fluorescent microscope and processed with ImageJ.

### Cell-Cycle Analysis

Cell-cycle analyses were performed using A549 cells fixed in ice-cold 70%_v/v_ ethanol. Cells were incubated with 100 μg/mL RNase A (Thermo Fisher Scientific), stained with 20 μg/mL propidium iodide, and then analyzed using Beckman Coulter CytoFLEX S. Representative FACS profiles were generated using Beckman CytExpert software.

## Supporting Information Available

Mass spectrometry inhibition assays; crystallographic analysis; small-molecule synthesis; general synthetic procedures; experimental procedures; and NMR and HPLC analysis (PDF)
Molecular formula strings (CSV)

## Abbreviations

AspH: aspartate/asparagine-β-hydroxylase
FIH: factor inhibiting HIF-α
HIF-α: hypoxia-inducible transcription factor-α
JMJD5: Jumonji-C domain-containing protein 5
KDM4E: JmjC histone *N*^ε^-dimethyllysine-specific demethylase 4E
2OG: 2-oxoglutarate
PHD2: HIF-α prolyl residue hydroxylase 2
2,4-PDCA: pyridine-2,4-dicarboxylic acid
RIOX2: ribosomal oxygenase 2
RPS6: 40S ribosomal protein S6
