# Supplementary material for: 5-Substituted Pyridine-2,4-dicarboxylate Derivatives Have Potential for Selective Inhibition of Human Jumonji-C Domain-Containing Protein 5
Source: J Med Chem. 2023 Aug 1;66(15):10849–65. doi: 10.1021/acs.jmedchem.3c01114 (PMC10424186; doi:10.1021/acs.jmedchem.3c01114)
Supplement: Supplementary file 1 — jm3c01114_si_001.pdf [file jm3c01114_si_001.pdf]

Supporting Information

5-Substituted pyridine-2,4-dicarboxylate derivatives have potential for selective inhibition of human Jumonji-C domain-containing protein 5

Lennart Brewitz[1,*], Yu Nakashima[1,2], Sonia K. Piasecka[3], Eidarus Salah[1], Sally C. Fletcher[3], Anthony Tumber[1], Thomas P. Corner[1], Tristan J. Kennedy[3], Giorgia Fiorini[1], Armin Thalhammer[1], Kirsten E. Christensen[4], Mathew L. Coleman[3,*], and Christopher J. Schofield[1,*]

[1]*Chemistry Research Laboratory, Department of Chemistry and the Ineos Oxford Institute for Antimicrobial Research, University of Oxford, 12 Mansfield Road, OX1 3TA, Oxford, United Kingdom.*

[2]*Present address: Institute of Natural Medicine, University of Toyama, 2630-Sugitani, 930-0194, Toyama, Japan.*

[3]*Institute of Cancer and Genomic Sciences, University of Birmingham, Edgbaston, B15 2TT, Birmingham, United Kingdom.*

[4]*Chemical Crystallography, Chemistry Research Laboratory, Department of Chemistry, University of Oxford, 12 Mansfield Road, OX1 3TA, Oxford, United Kingdom.*

*E-mail: lennart.brewitz@chem.ox.ac.uk, m.coleman@bham.ac.uk, and christopher.schofield@chem.ox.ac.uk

Table of contents

1. Supporting figures S2-S26
2. Supporting tables S27-S30
3. General information S31
4. General synthetic procedures S32
5. Synthetic procedures and analytical data S33-S40
6. References S41-S42
7. ^{1}H and ^{13}C NMR spectra of C5 substituted 2,4-PDCA derivatives prepared for this study S43-S52
8. HPLC traces of C5 substituted 2,4-PDCA derivatives prepared for this study S53-S56

1. Supporting figures

Supporting Figure S1. Representative dose-response curves used to determine IC_{50} values for the inhibition of JMJD5 by 2,4-PDCA derivatives bearing 3-aminoalkyl, 3-aminoaryl-, fluoro or trifluoromethyl substituents. SPE-MS inhibition assays were performed as described using isolated recombinant JMJD5 (0.15 μM), 2OG (2.0 μM), Fe(II) (2.0 μM), LAA (100 μM), and $RPS6_{128-148}$ (2.0 μM) in buffer (50 mM MOPS, pH 7.5, 20 °C).[1] Dose-response curves are a mean of two technical duplicates (n = 2; mean ± standard deviation, SD). The mean of three independent triplicates each composed of technical duplicates was used to determine IC_{50} values (Supporting Table S1).

(**a**) **1**: orange triangles, **21**: green circles, **22**: red boxes, **23**: cyan diamonds, **24**: ochre inverse triangles, **2**: black circles, and **25**: violet diamond; (**b**) **26**: orange triangles, **27**: green circles, **28**: red boxes, **29**: cyan diamonds, **3**: ochre inverse triangles, **4**: black circles, and **5**: violet diamond; (**c**) **6**: orange triangles, **7**: green circles, **8**: red boxes, **30**: cyan diamonds, **9**: ochre inverse triangles, **10**: black circles, and **31**: violet diamond; (**d**) **32**: orange triangles, **11**: green circles, **12**: red boxes, **13**: cyan diamonds, **14**: ochre inverse triangles, **15**: black circles, and **16**: violet diamond.

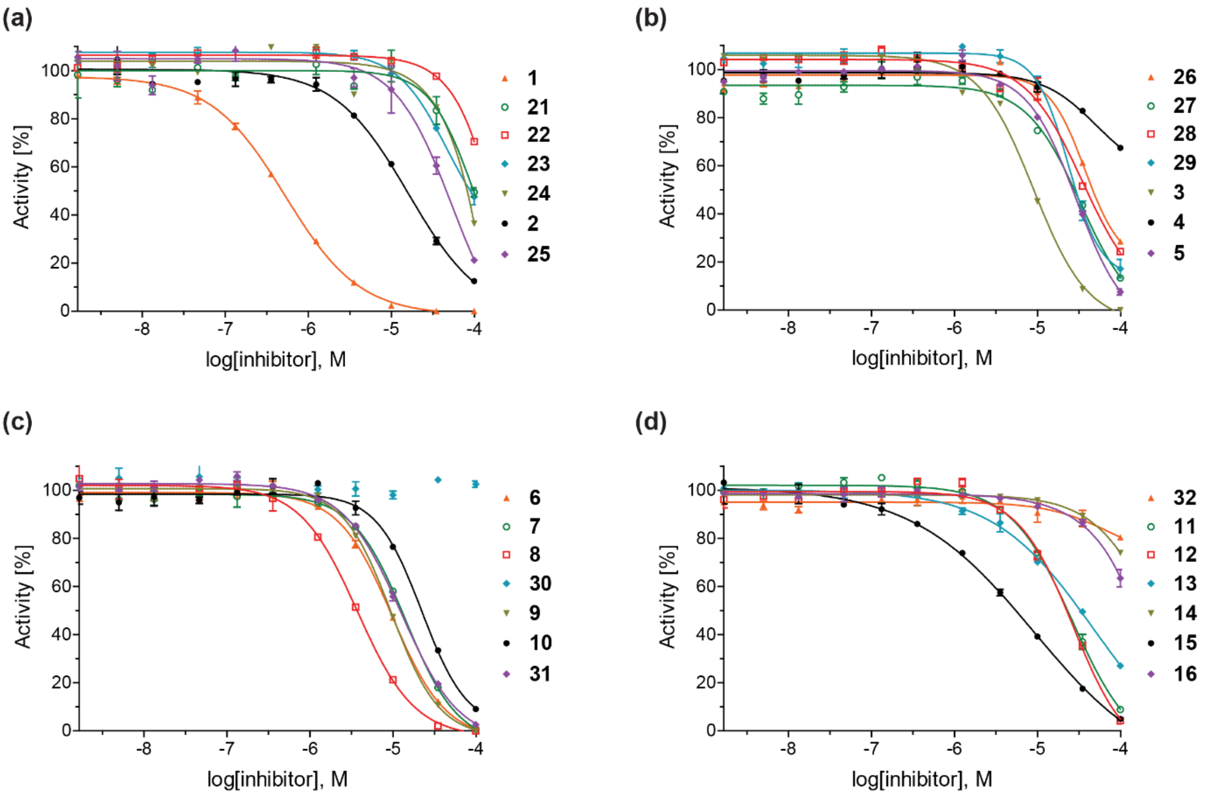

Supporting Figure S2. Robustness of the JMJD5 SPE-MS inhibition assays. (**a**) Z'-factors[2] and (**b**) signal-to-noise ratios (S/N) for the JMJD5 inhibition assay plates analysed to determine IC_{50}-values. The Z'-factors >0.5 (grey line) indicate a stable and robust assay of high quality.[2] Z'-factors and S/N ratios were determined according to the literature using Microsoft Excel.[2]

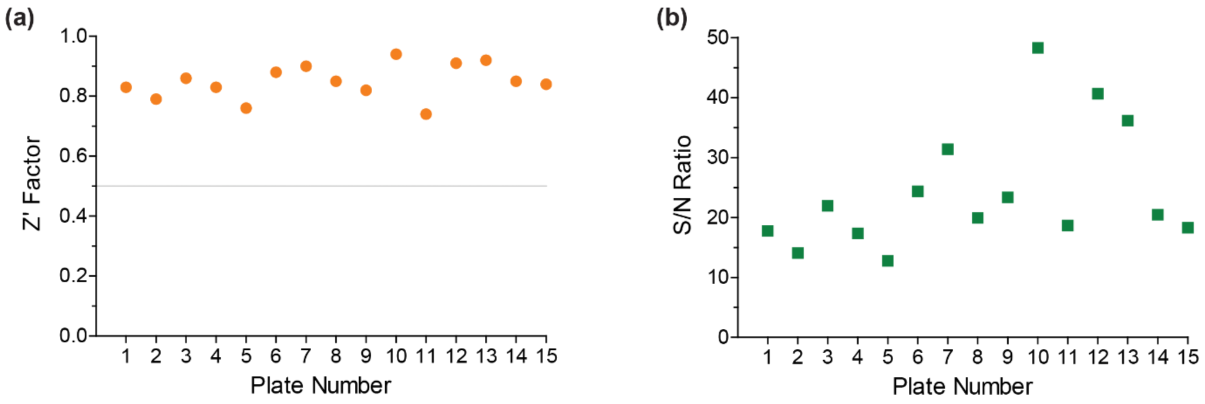

Supporting Figure S3. Crystallographic analysis confirms the structural assignment of the Buchwald-Hartwig reaction product dimethyl 5-aminobenzyl-2,4-PDCA. Diffraction analysis of a single crystal of dimethyl 5-aminobenzyl-2,4-PDCA (**19a**) confirms its structure as assigned by ^{1}H and ^{13}C NMR, IR, and MS analysis. Colour code: white: hydrogens; grey: carbons; blue: nitrogens; red: oxygens. Selected crystallographic data are shown in Supporting Table S2**.**

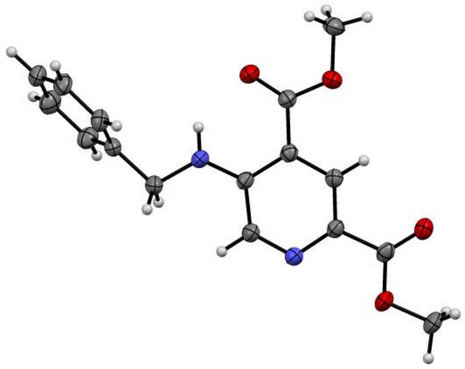

Supporting Figure S4. Reaction schemes of the human 2OG oxygenases used in this work for selectivity studies. (**a**) Prolyl hydroxylase domain-containing protein 2 (PHD2) catalyses the stereoselective C4 hydroxylation of Pro564 of the hypoxia inducible transcription factor-1α (HIF-1α);[3-4] (**b**) factor inhibiting hypoxia inducible transcription factor-1α (FIH) catalyses the stereoselective C3 hydroxylation of Asn/Asp/His/Ser/Leu-residues,[5] such as, for example, the hydroxylation of Asn803 of HIF-1α;[6] (**c**) aspartate/asparagine-β-hydroxylase (AspH) catalyses the stereoselective C3 hydroxylation of Asp/Asn-residues which are part of specific disulphide isomers of epidermal growth factor-like domains (EGFDs);[7-9] (**d**) JmjC lysine-specific N^{ε}-demethylase 4E (KDM4E, JMJD2E) catalyses the N^{ε}-demethylation of histone 3 (H3) N^{ε}-di- and trimethylated Lys9 (H3K9me3/me2) via methyl-group oxidation with the formation of formaldehyde as a coproduct;[10] (**e**) ribosomal oxygenase 2 (RIOX2; MYC-induced nuclear antigen 53 or MINA53) catalyses the stereoselective C3 hydroxylation of His39 of the 60S ribosomal protein L27a (RPL27A).[11]

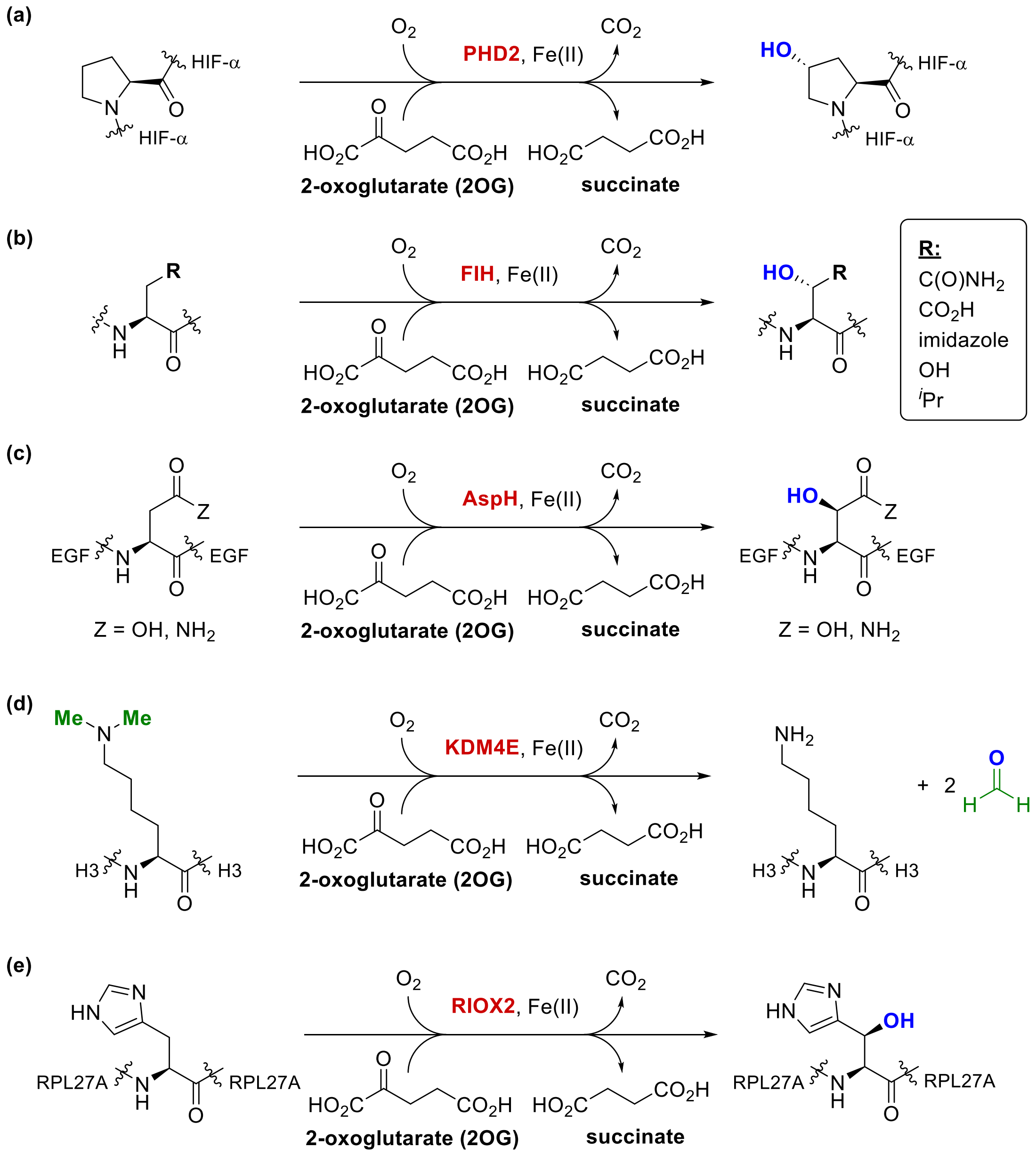

Supporting Figure S5. Views from a crystal structure of JMJD5 complexed with 5-aminobenzylpyridine-2,4-dicarboxylic acid (20a) and Mn (JMJD5:20a; PDB ID: 7DYV). Colour code: grey: JMJD5; purple: carbon-backbone of 5-aminobenzylpyridine-2,4-dicarboxylic acid (**20a**); lavender blue: Mn; red: oxygen; blue: nitrogen. w: water.

(**a**) Overview of the JMJD5:**20a** complex structure (1.92 Å resolution); (**b**) active site view of the JMJD5:**20a** complex structure reveals that the pyridine heterocycle of **20a** is positioned to interact with the indole side chain of W310 via π-stacking (distance from the centers of the pyridine and W310 phenyl rings: 3.5 Å), that the C4 carboxylate of the 2,4-PDCA core is positioned to interact with the side chain amino group of K336 (2.5 Å) and the side chain hydroxyl group of Y272 (2.8 Å), and that the C2 carboxylate is positioned to interact with the side chain amide group of N327 (2.8 and 3.0 Å) and the indole NH group of W414 (3.4 Å). In addition, the C2 carboxylate is positioned to chelate the Mn ion (2.2 Å) together with the pyridine N atom (2.4 Å). The Mn ion is also complexed by the side chain carboxylate of D323 (2.2 Å) and the side chain imidazole *N*τ atoms of H321 (2.4 Å) and H400 (2.4 Å); a water molecule ligates the Mn ion (2.0 Å). The phenyl group of **20a** occupies a conformation in which its *para* C–H is positioned to interact with the indole side chain of W248 via a σ-π interaction (distance C_{para} to the center of the W248 phenyl ring: 3.5 Å).

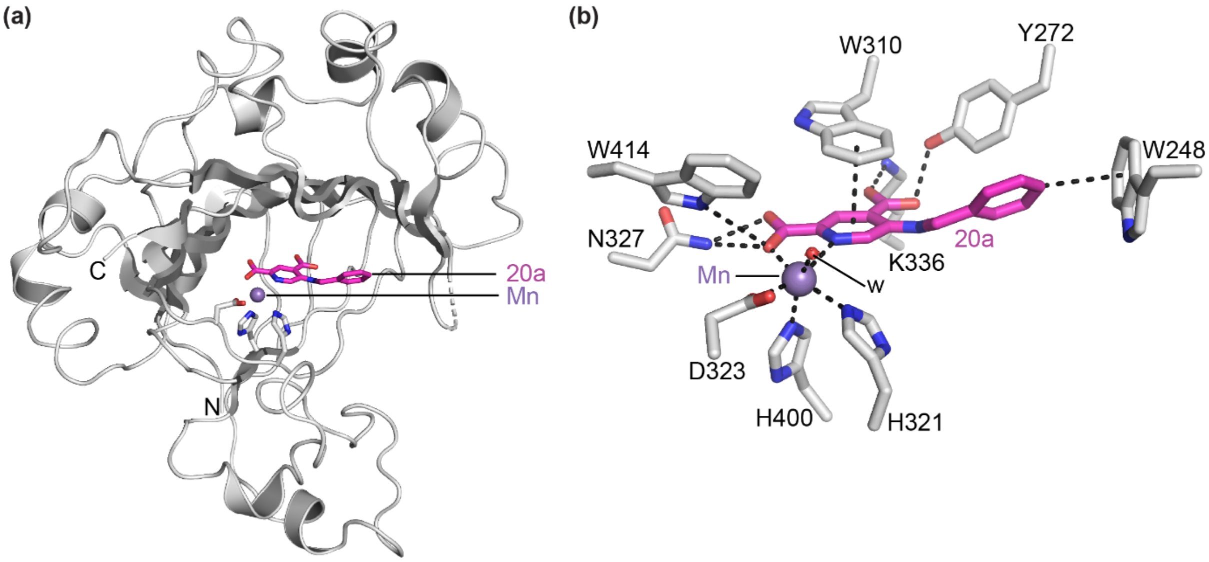

Supporting Figure S6. Views from a crystal structure of JMJD5 complexed with 5-((4-phenylbutyl)amino)pyridine-2,4-dicarboxylic acid (20d) and Mn (JMJD5:20d; PDB ID: 7DYU). Colour code: grey: JMJD5; green: carbon-backbone of 5-((4-phenylbutyl)amino)pyridine-2,4-dicarboxylic acid (**20d**); lavender blue: Mn; red: oxygen; blue: nitrogen. w: water.

(**a**) Overview of the JMJD5:**20d** complex structure (1.72 Å resolution); (**b**) active site view of the JMJD5:**20d** complex structure reveals that the pyridine heterocycle of **20d** is positioned to interact with the indole side chain of W310 via π-stacking (distance from the centers of the pyridine and W310 phenyl rings: 3.7 Å), that the C4 carboxylate of the 2,4-PDCA core is positioned to interact with the side chain amino group of K336 (2.6 Å) and the side chain hydroxyl group of Y272 (2.5 Å), and that the C2 carboxylate is positioned to interact with the side chain amide group of N327 (2.9 and 3.0 Å) and the indole NH group of W414 (3.2 Å). In addition, the C2 carboxylate is positioned to chelate the Mn ion (2.2 Å) together with the pyridine N atom (2.3 Å). The Mn ion is also complexed by the side chain carboxylate of D323 (2.2 Å) and the side chain imidazole $N\tau$ atoms of H321 (2.2 Å) and H400 (2.4 Å); a water molecule ligates the Mn ion (2.2 Å).

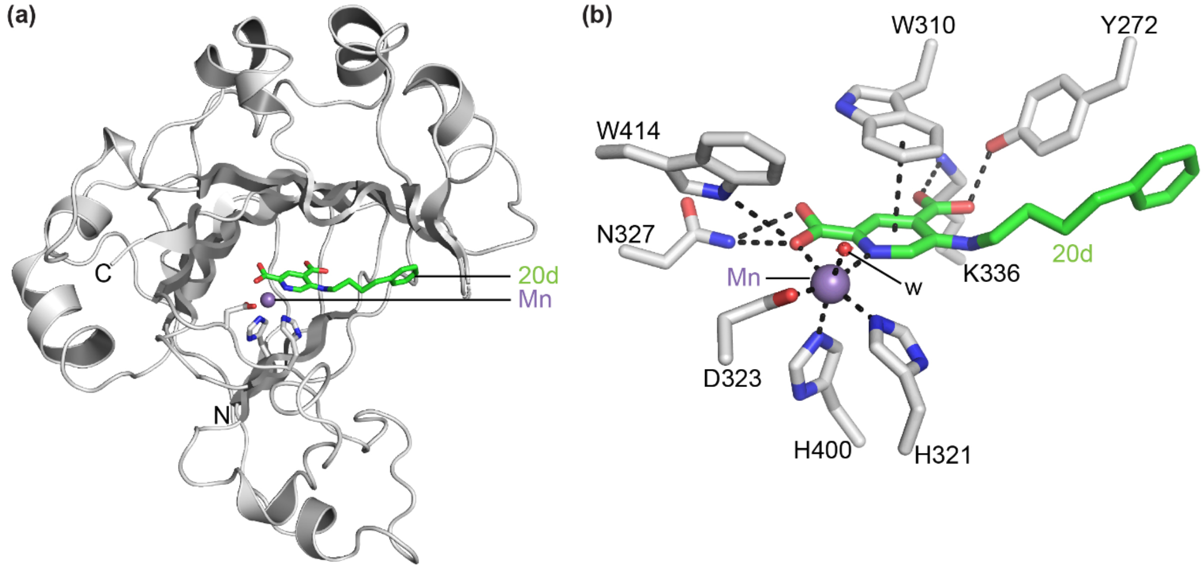

Supporting Figure S7. Views from a crystal structure of JMJD5 complexed with 5-((4-methoxybenzyl)amino)pyridine-2,4-dicarboxylic acid (20h) and Mn (JMJD5:20h; PDB ID: 7DYT). Colour code: grey: JMJD5; cyan: carbon-backbone of 5-((4-methoxybenzyl)amino)pyridine-2,4-dicarboxylic acid (**20h**); lavender blue: Mn; red: oxygen; blue: nitrogen. w: water.
(**a**) Overview of the JMJD5:**20h** complex structure (1.62 Å resolution); (**b**) active site view of the JMJD5:**20h** complex structure reveals that the pyridine heterocycle of **20h** is positioned to interact with the indole side chain of W310 via π-stacking (distance from the centers of the pyridine and W310 phenyl rings: 3.6 Å), that the C4 carboxylate of the 2,4-PDCA core is positioned to interact with the side chain amino group of K336 (2.7 Å) and the side chain hydroxyl group of Y272 (2.7 Å), and that the C2 carboxylate is positioned to interact with the side chain amide group of N327 (2.8 and 3.0 Å) and the indole NH group of W414 (3.3 Å). In addition, the C2 carboxylate is positioned to chelate the Mn ion (2.1 Å) together with the pyridine N atom (2.5 Å). The Mn ion is also complexed by the side chain carboxylate of D323 (2.2 Å) and the side chain imidazole *N*τ atoms of H321 (2.2 Å) and H400 (2.3 Å); a water molecule ligates the Mn ion (2.3 Å).

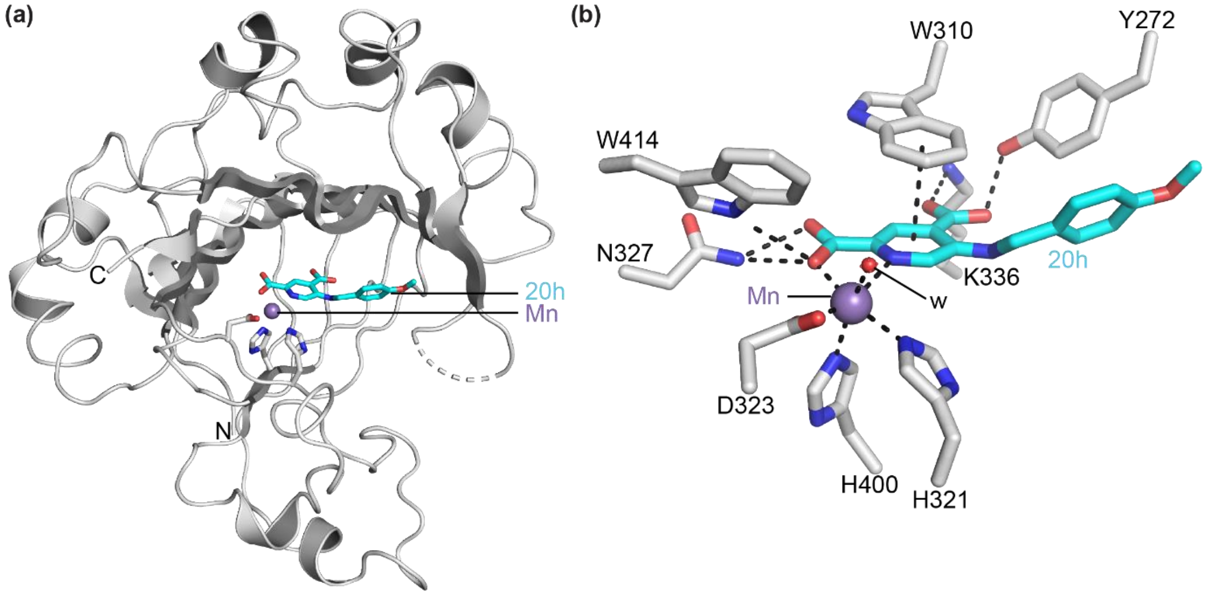

Supporting Figure S8. Views from a crystal structure of JMJD5 complexed with 5-((2-methoxybenzyl)amino)pyridine-2,4-dicarboxylic acid (20i) and Mn (JMJD5:20i; PDB ID: 7DYW). Colour code: grey: JMJD5; orange: carbon-backbone of 5-((2-methoxybenzyl)amino)pyridine-2,4-dicarboxylic acid (**20i**); lavender blue: Mn; red: oxygen; blue: nitrogen. w: water.

(**a**) Overview of the JMJD5:**20i** complex structure (2.13 Å resolution); (**b**) active site view of the JMJD5:**20i** complex structure reveals that the pyridine heterocycle of **20i** is positioned to interact with the indole side chain of W310 via π-stacking (distance from the centers of the pyridine and W310 phenyl rings: 3.7 Å), that the C4 carboxylate of the 2,4-PDCA core is positioned to interact with the side chain amino group of K336 (2.5 Å) and the side chain hydroxyl group of Y272 (2.6 Å), and that the C2 carboxylate is positioned to interact with the side chain amide group of N327 (2.7 and 2.9 Å) and the indole NH group of W414 (3.5 Å). In addition, the C2 carboxylate is positioned to chelate the Mn ion (2.3 Å) together with the pyridine N atom (2.3 Å). The Mn ion is also complexed by the side chain carboxylate of D323 (2.2 Å) and the side chain imidazole *N*τ atoms of H321 (2.2 Å) and H400 (2.4 Å); a water molecule ligates the Mn ion (2.4 Å). The 2-methoxyphenyl group of **20i** occupies a conformation in which its *para* C–H is positioned to interact with the indole side chain of W248 via a σ-π interaction (distance C_{para} to the center of the W248 phenyl ring: 3.7 Å).

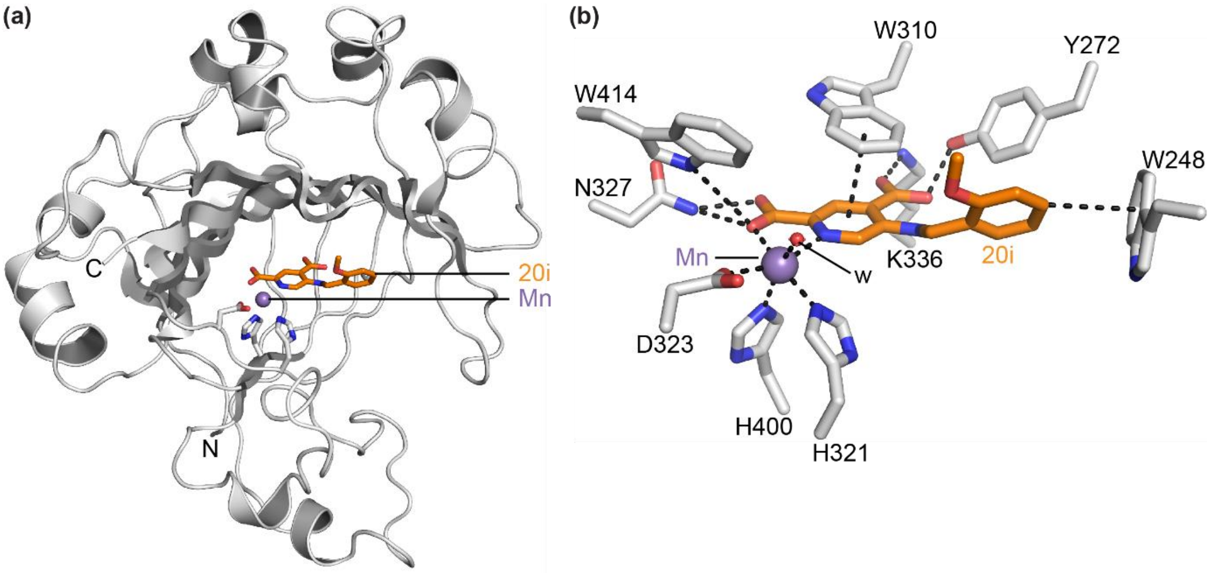

Supporting Figure S9. Views from a crystal structure of JMJD5 complexed with 5-((2-cyclopropylbenzyl)amino)pyridine-2,4-dicarboxylic acid (20j) and Mn (JMJD5:20j; PDB ID: 7DYX). Colour code: grey: JMJD5; salmon: carbon-backbone of 5-((2-cyclopropylbenzyl)amino)pyridine-2,4-dicarboxylic acid (**20j**); lavender blue: Mn; red: oxygen; blue: nitrogen. w: water.
(**a**) Overview of the JMJD5:**20j** complex structure (2.27 Å resolution); (**b**) active site view of the JMJD5:**20j** complex structure reveals that the pyridine heterocycle of **20j** is positioned to interact with the indole side chain of W310 via π-stacking (distance from the centers of the pyridine and W310 phenyl rings: 3.6 Å), that the C4 carboxylate of the 2,4-PDCA core is positioned to interact with the side chain amino group of K336 (2.5 Å) and the side chain hydroxyl group of Y272 (2.7 Å), and that the C2 carboxylate is positioned to interact with the side chain amide group of N327 (2.7 and 2.8 Å) and the indole NH group of W414 (3.5 Å). In addition, the C2 carboxylate is positioned to chelate the Mn ion (2.2 Å) together with the pyridine N atom (2.3 Å). The Mn ion is also complexed by the side chain carboxylate of D323 (2.2 Å) and the side chain imidazole $N\tau$ atoms of H321 (2.2 Å) and H400 (2.2 Å); a water molecule ligates the Mn ion (1.8 Å). The 2-methoxyphenyl group of **20j** occupies a conformation in which its *para* C–H is positioned to interact with the indole side chain of W248 via a σ-π interaction (distance C_{para} to the center of the W248 phenyl ring: 3.4 Å).

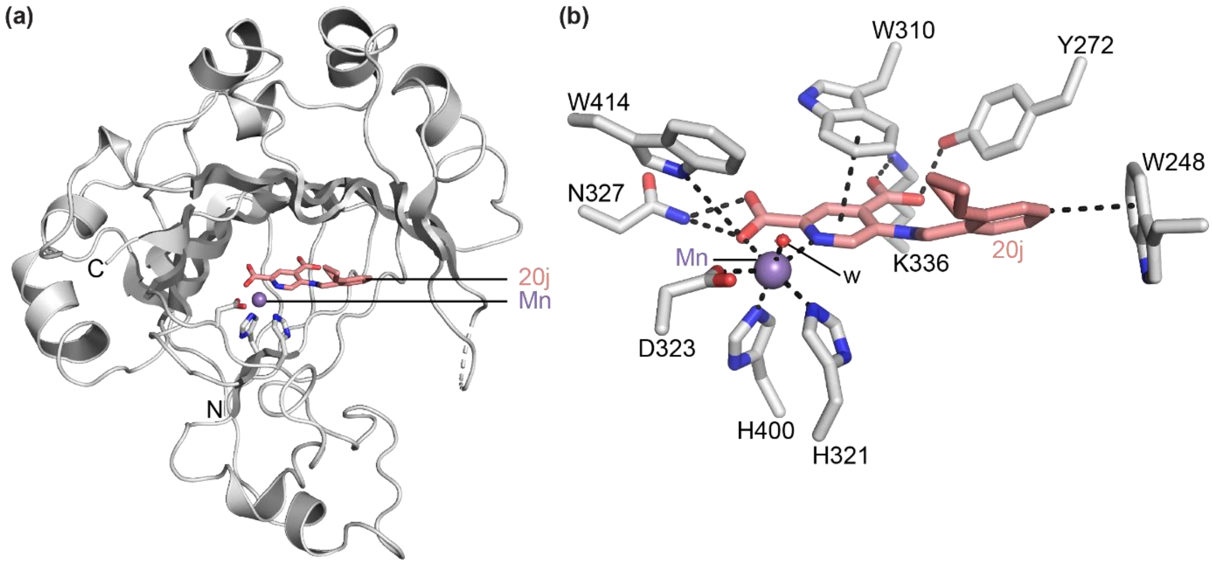

Supporting Figure S10. 5-Aminobenzylpyridine-2,4-dicarboxylic acid (20a) binds to JMJD5 in a similar manner as pyridine-2,4-dicarboxylic acid (1) and 2OG (continues on the following page). Colour code: purple: carbon-backbone of 5-aminobenzylpyridine-2,4-dicarboxylic acid (**20a**); yellow: carbon-backbone of 2,4-PDCA (**1**); slate blue: carbon-backbone of 2OG; red: oxygen; blue: nitrogen. w: water.
(**a**) Superimposition of views from the JMJD5:**20a** (grey: JMJD5; lavender blue: Mn; Supporting Figure S5) and the reported JMJD5:**1** (teal: JMJD5; pink: Mn; PDB ID: 6I9L[12]) complex structures reveals similar JMJD5 conformations (Cα RMSD = 0.149 Å). Note the conformational flexibility of the W248-bearing loop (G240 to W248) in proximity of the C5 benzyl substituent of **20a**; (**b**) superimposition of views from the active sites of the JMJD5:**20a** (grey: JMJD5; lavender blue: Mn; Supporting Figure S5) and the reported JMJD5:**1** (teal: JMJD5; pink: Mn; PDB ID: 6I9L[12]) complex structures reveals similar conformations of the side chains of important JMJD5 active site residues. Note that electron density for the indole ring of W248 was not observed in the reported JMJD5:**1** structure. The superimposition implies that both **20a** and **1** bind to JMJD5 in a similar manner; (**c**) superimposition of views from the JMJD5:**20a** (grey: JMJD5; lavender blue: Mn; Supporting Figure S5) and the reported JMJD5:2OG (brown: JMJD5; pink: Mn; PDB ID: 6F4N[13]) complex structures reveals similar JMJD5 conformations (Cα RMSD = 0.235 Å). Note the conformational flexibility of the W248-bearing loop (G240 to W248) in proximity of the C5 benzyl substituent of **20a** and that two JMJD5 molecules are present in the asymmetric unit of the reported JMJD5:2OG structure;[13] (**d**) superimposition of views from the active sites of the JMJD5:**20a** (grey: JMJD5; lavender blue: Mn; Supporting Figure S5) and the reported JMJD5:2OG (brown: JMJD5; pink: Mn; PDB ID: 6F4N[13]) complex structures reveals similar conformations of the side chains of important JMJD5 active site residues with the notable exception of W248. The superimposition implies the C2 and C4 carboxylate groups of **20a** interact with JMJD5 in a similar manner as the C1 and C5 carboxylate groups of 2OG and thus that both **20a** and 2OG bind JMJD5 in a similar manner.

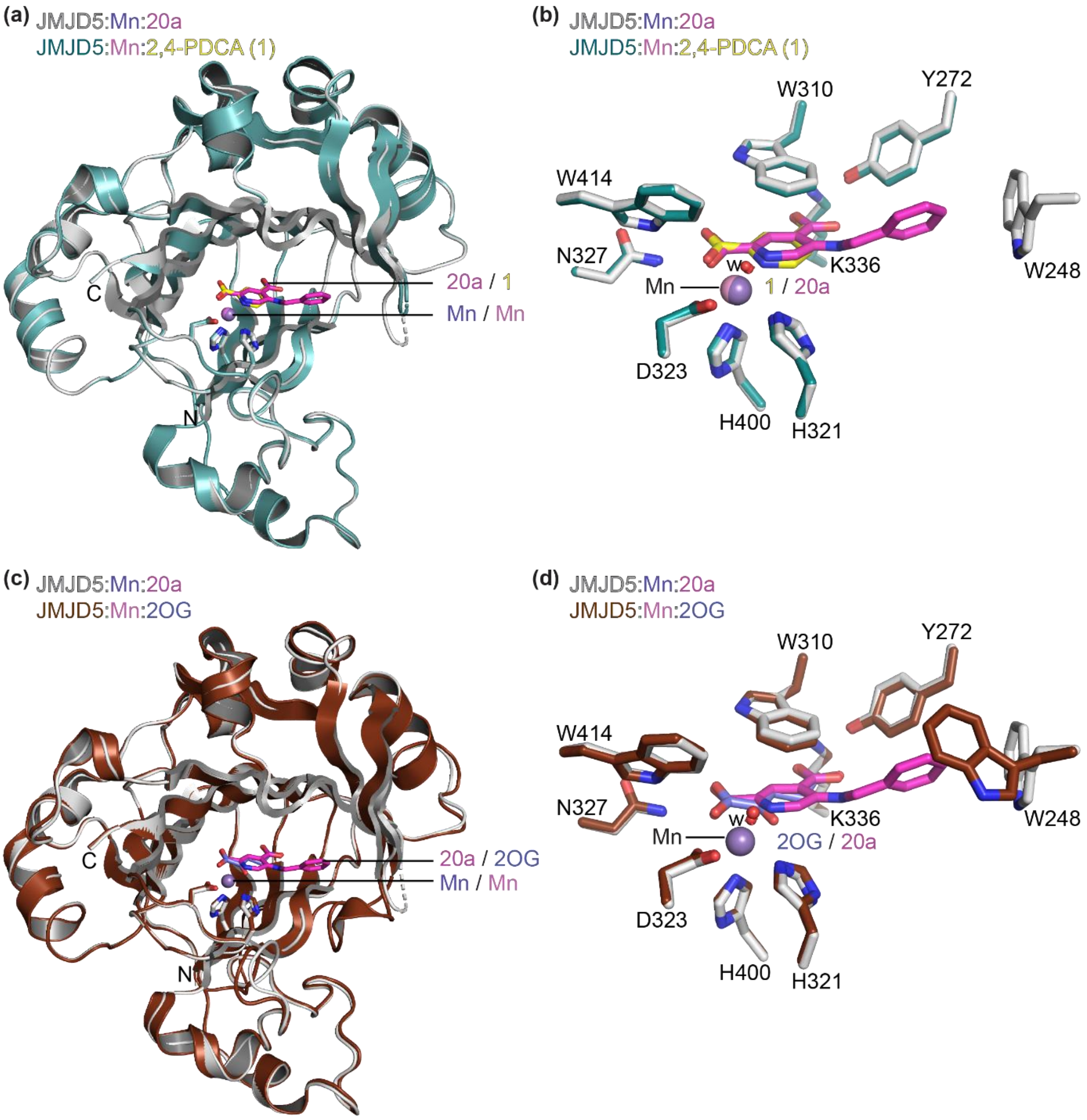
(a) JMJD5:Mn:20a
JMJD5:Mn:2,4-PDCA (1)
C
N
20a / 1
Mn / Mn
(b) JMJD5:Mn:20a
JMJD5:Mn:2,4-PDCA (1)
W310
Y272
W414
N327
w
K336
W248
Mn
1 / 20a
D323
H400
H321
(c) JMJD5:Mn:20a
JMJD5:Mn:2OG
C
N
20a / 2OG
Mn / Mn
(d) JMJD5:Mn:20a
JMJD5:Mn:2OG
W310
Y272
W414
N327
w
K336
W248
Mn
2OG / 20a
D323
H400
H321

Supporting Figure S11. 5-((4-Phenylbutyl)amino)pyridine-2,4-dicarboxylic acid (20d) binds to JMJD5 in a similar manner as pyridine-2,4-dicarboxylic acid (1) and 2OG (continues on the following page). Colour code: green: carbon-backbone of 5-((4-phenylbutyl)amino)pyridine-2,4-dicarboxylic acid (**20d**); yellow: carbon-backbone of 2,4-PDCA (**1**); slate blue: carbon-backbone of 2OG; red: oxygen; blue: nitrogen. w: water.
(**a**) Superimposition of views from the JMJD5:**20d** (grey: JMJD5; lavender blue: Mn; Supporting Figure S6) and the reported JMJD5:**1** (teal: JMJD5; pink: Mn; PDB ID: 6I9L[12]) complex structures reveals similar JMJD5 conformations (Cα RMSD = 0.115 Å); (**b**) superimposition of views from the active sites of the JMJD5:**20d** (grey: JMJD5; lavender blue: Mn; Supporting Figure S6) and the reported JMJD5:**1** (teal: JMJD5; pink: Mn; PDB ID: 6I9L[12]) complex structures reveals similar conformations of the side chains of important JMJD5 active site residues. The superimposition implies that both **20d** and **1** bind to JMJD5 in a similar manner; (**c**) superimposition of views from the JMJD5:**20d** (grey: JMJD5; lavender blue: Mn; Supporting Figure S6) and the reported JMJD5:2OG (brown: JMJD5; pink: Mn; PDB ID: 6F4N[13]) complex structures reveals similar JMJD5 conformations (Cα RMSD = 0.226 Å). Note the conformational flexibility of the W248-bearing loop (G240 to W248) in proximity of the **20d** C5 substituent's phenyl group and that two JMJD5 molecules are present in the asymmetric unit of the reported JMJD5:2OG structure;[13] (**d**) superimposition of views from the active sites of the JMJD5:**20d** (grey: JMJD5; lavender blue: Mn; Supporting Figure S6) and the reported JMJD5:2OG (brown: JMJD5; pink: Mn; PDB ID: 6F4N[13]) complex structures reveals similar conformations of the side chains of important JMJD5 active site residues. The superimposition implies the C2 and C4 carboxylate groups of **20d** interact with JMJD5 in a similar manner as the C1 and C5 carboxylate groups of 2OG and thus that both **20d** and 2OG bind JMJD5 in a similar manner.

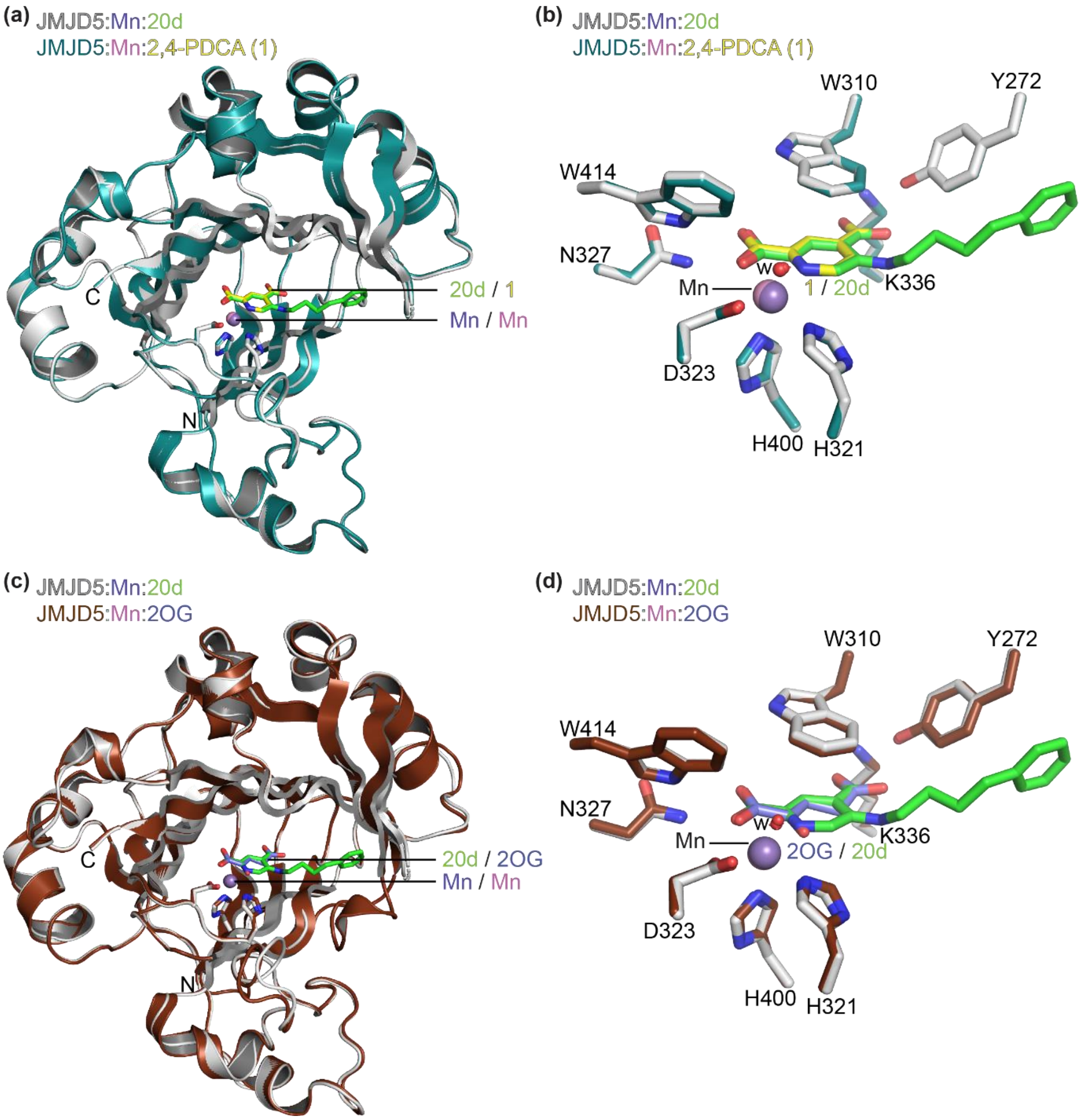
(a) JMJD5:Mn:20d
JMJD5:Mn:2,4-PDCA (1)
C
N
20d / 1
Mn / Mn
(b) JMJD5:Mn:20d
JMJD5:Mn:2,4-PDCA (1)
W310
Y272
W414
N327
w
Mn
1 / 20d
K336
D323
H400
H321
(c) JMJD5:Mn:20d
JMJD5:Mn:2OG
C
N
20d / 2OG
Mn / Mn
(d) JMJD5:Mn:20d
JMJD5:Mn:2OG
W310
Y272
W414
N327
w
Mn
2OG / 20d
K336
D323
H400
H321

Supporting Figure S12. 5-((4-Methoxybenzyl)amino)pyridine-2,4-dicarboxylic acid (20h) binds to JMJD5 in a similar manner as pyridine-2,4-dicarboxylic acid (1) and 2OG (continues on the following page). Colour code: cyan: carbon-backbone of 5-((4-methoxybenzyl)amino)pyridine-2,4-dicarboxylic acid (**20h**); yellow: carbon-backbone of 2,4-PDCA (**1**); slate blue: carbon-backbone of 2OG; red: oxygen; blue: nitrogen. w: water. (**a**) Superimposition of views from the JMJD5:**20h** (grey: JMJD5; lavender blue: Mn; Supporting Figure S7) and the reported JMJD5:**1** (teal: JMJD5; pink: Mn; PDB ID: 6I9L[12]) complex structures reveals similar JMJD5 conformations (Cα RMSD = 0.139 Å). Note the conformational flexibility of the W248-bearing loop (G240 to W248) in proximity of the C5 benzyl substituent of **20h**; (**b**) superimposition of views from the active sites of the JMJD5:**20h** (grey: JMJD5; lavender blue: Mn; Supporting Figure S7) and the reported JMJD5:**1** (teal: JMJD5; pink: Mn; PDB ID: 6I9L[12]) complex structures reveals similar conformations of the side chains of important JMJD5 active site residues. The superimposition implies that both **20h** and **1** bind to JMJD5 in a similar manner; (**c**) superimposition of views from the JMJD5:**20h** (grey: JMJD5; lavender blue: Mn; Supporting Figure S7) and the reported JMJD5:2OG (brown: JMJD5; pink: Mn; PDB ID: 6F4N[13]) complex structures reveals similar JMJD5 conformations (Cα RMSD = 0.222 Å). Note the conformational flexibility of the W248-bearing loop (G240 to W248) in proximity of the **20h** C5 substituent's phenyl group and that two JMJD5 molecules are present in the asymmetric unit of the reported JMJD5:2OG structure;[13] (**d**) superimposition of views from the active sites of the JMJD5:**20h** (grey: JMJD5; lavender blue: Mn; Supporting Figure S7) and the reported JMJD5:2OG (brown: JMJD5; pink: Mn; PDB ID: 6F4N[13]) complex structures reveals similar conformations of the side chains of important JMJD5 active site residues. The superimposition implies the C2 and C4 carboxylate groups of **20h** interact with JMJD5 in a similar manner as the C1 and C5 carboxylate groups of 2OG and thus that both **20h** and 2OG bind JMJD5 in a similar manner.

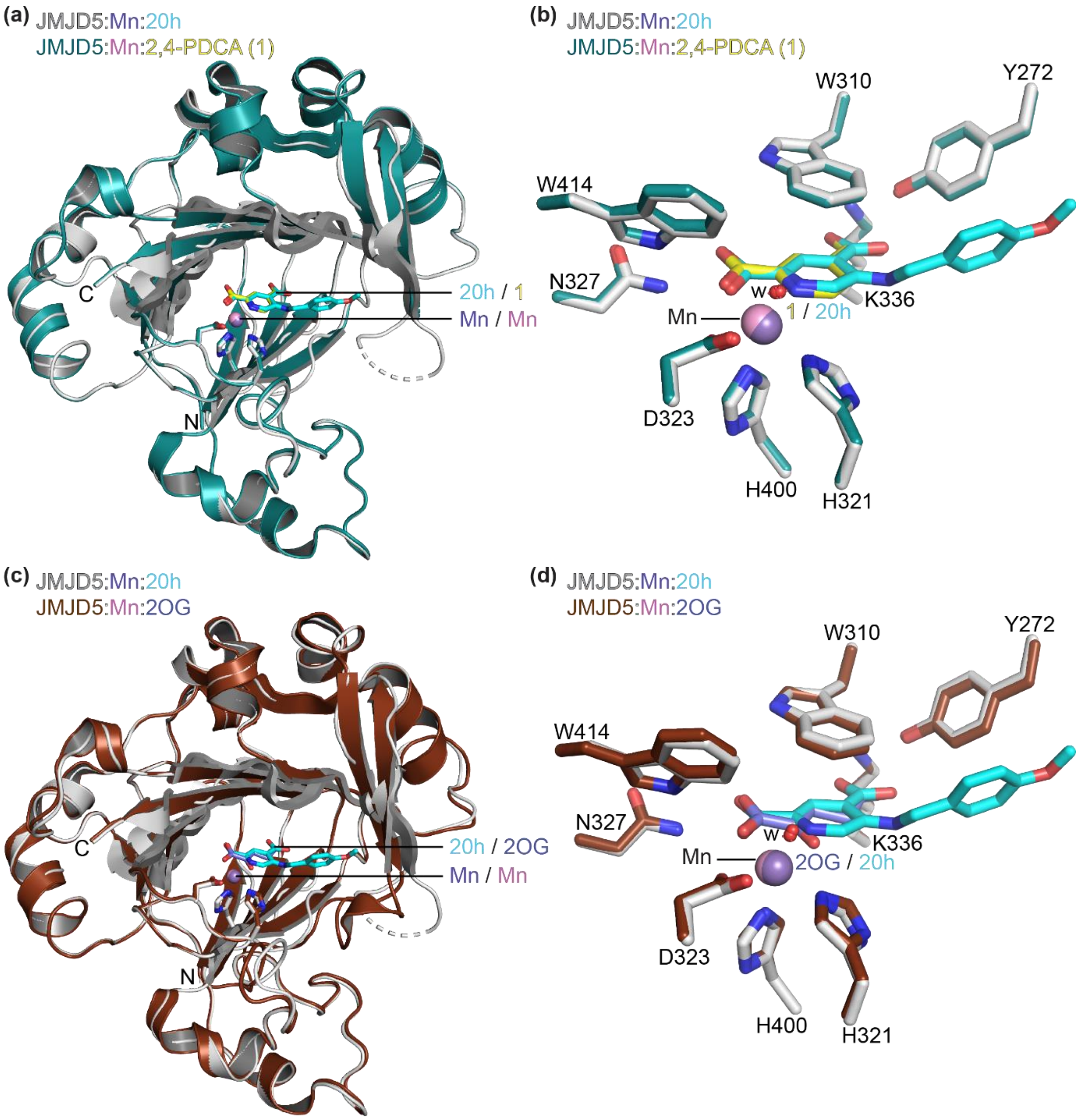
(a)
JMJD5:Mn:20h
JMJD5:Mn:2,4-PDCA (1)
C
N
20h / 1
Mn / Mn
(b)
JMJD5:Mn:20h
JMJD5:Mn:2,4-PDCA (1)
W310
Y272
W414
N327
w
K336
Mn
1 / 20h
D323
H400
H321
(c)
JMJD5:Mn:20h
JMJD5:Mn:2OG
C
N
20h / 2OG
Mn / Mn
(d)
JMJD5:Mn:20h
JMJD5:Mn:2OG
W310
Y272
W414
N327
w
K336
Mn
2OG / 20h
D323
H400
H321

Supporting Figure S13. 5-((2-Methoxybenzyl)amino)pyridine-2,4-dicarboxylic acid (20i) binds to JMJD5 in a similar manner as pyridine-2,4-dicarboxylic acid (1) and 2OG (continues on the following page). Colour code: orange: carbon-backbone of 5-((2-methoxybenzyl)amino)pyridine-2,4-dicarboxylic acid (**20i**); yellow: carbon-backbone of 2,4-PDCA (**1**); slate blue: carbon-backbone of 2OG; red: oxygen; blue: nitrogen. w: water. (**a**) Superimposition of views from the JMJD5:**20i** (grey: JMJD5; lavender blue: Mn; Supporting Figure S8) and the reported JMJD5:**1** (teal: JMJD5; pink: Mn; PDB ID: 6I9L[12]) complex structures reveals similar JMJD5 conformations (Cα RMSD = 0.182 Å). Note the conformational flexibility of the W248-bearing loop (G240 to W248) in proximity of the C5 benzyl substituent of **20i**; (**b**) superimposition of views from the active sites of the JMJD5:**20i** (grey: JMJD5; lavender blue: Mn; Supporting Figure S8) and the reported JMJD5:**1** (teal: JMJD5; pink: Mn; PDB ID: 6I9L[12]) complex structures reveals similar conformations of the side chains of important JMJD5 active site residues. Note that electron density for the indole ring of W248 was not observed in the reported JMJD5:**1** structure. The superimposition implies that both **20i** and **1** bind to JMJD5 in a similar manner; (**c**) superimposition of views from the JMJD5:**20i** (grey: JMJD5; lavender blue: Mn; Supporting Figure S8) and the reported JMJD5:2OG (brown: JMJD5; pink: Mn; PDB ID: 6F4N[13]) complex structures reveals similar JMJD5 conformations (Cα RMSD = 0.247 Å). Note the conformational flexibility of the W248-bearing loop (G240 to W248) in proximity of the C5 substituent of **20i** and that two JMJD5 molecules are present in the asymmetric unit of the reported JMJD5:2OG structure;[13] (**d**) superimposition of views from the active sites of the JMJD5:**20i** (grey: JMJD5; lavender blue: Mn; Supporting Figure S8) and the reported JMJD5:2OG (brown: JMJD5; pink: Mn; PDB ID: 6F4N[13]) complex structures reveals similar conformations of the side chains of important JMJD5 active site residues with the notable exception of W248. The superimposition implies the C2 and C4 carboxylate groups of **20i** interact with JMJD5 in a similar manner as the C1 and C5 carboxylate groups of 2OG and thus that both **20i** and 2OG bind JMJD5 in a similar manner.

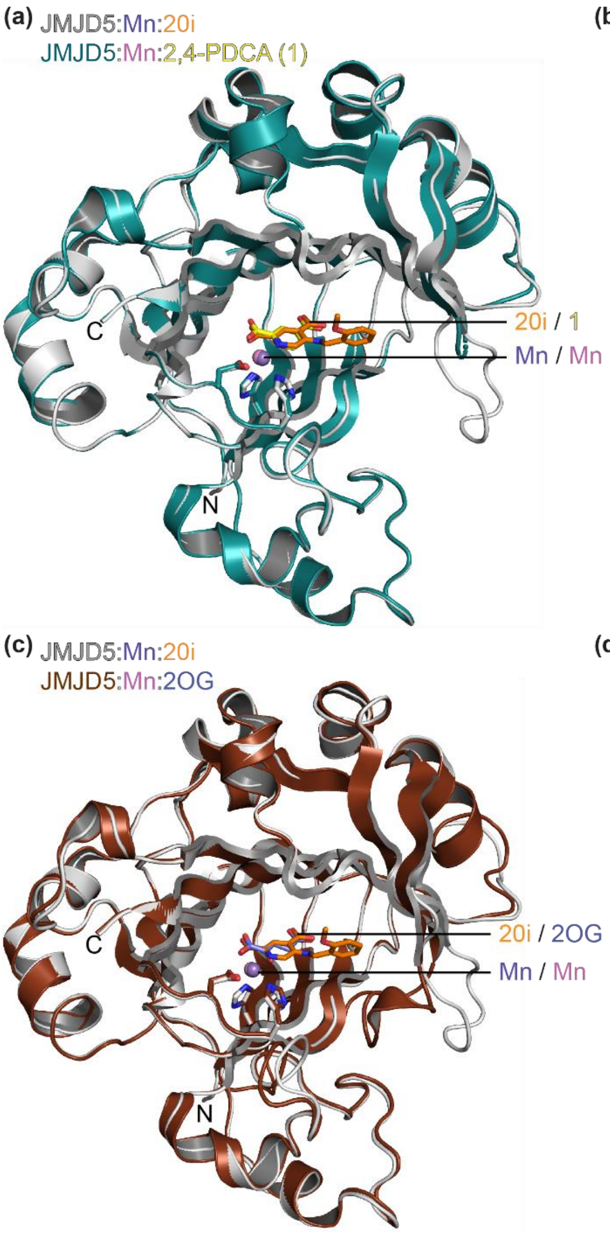
(a)
JMJD5:Mn:20i
JMJD5:Mn:2,4-PDCA (1)
C
N
20i / 1
Mn / Mn
(b)
JMJD5:Mn:20i
JMJD5:Mn:2,4-PDCA (1)
W310
Y272
W414
N327
W248
w
Mn
1 / 20i
K336
D323
H400
H321
(c)
JMJD5:Mn:20i
JMJD5:Mn:2OG
C
N
20i / 2OG
Mn / Mn
(d)
JMJD5:Mn:20i
JMJD5:Mn:2OG
W310
Y272
W414
N327
w
Mn
2OG / 20i
K336
W248
D323
H400
H321

Supporting Figure S14. 5-((2-Cyclopropylbenzyl)amino)pyridine-2,4-dicarboxylic acid (20j) binds to JMJD5 in a similar manner as pyridine-2,4-dicarboxylic acid (1) and 2OG (continues on the following page). Colour code: salmon: carbon-backbone of 5-((2-cyclopropylbenzyl)amino)pyridine-2,4-dicarboxylic acid (**20j**); yellow: carbon-backbone of 2,4-PDCA (**1**); slate blue: carbon-backbone of 2OG; red: oxygen; blue: nitrogen. w: water.

(**a**) Superimposition of views from the JMJD5:**20j** (grey: JMJD5; lavender blue: Mn; Supporting Figure S9) and the reported JMJD5:**1** (teal: JMJD5; pink: Mn; PDB ID: 6I9L[12]) complex structures reveals similar JMJD5 conformations (Cα RMSD = 0.240 Å). Note the conformational flexibility of the W248-bearing loop (G240 to W248) in proximity of the C5 benzyl substituent of **20j**; (**b**) superimposition of views from the active sites of the JMJD5:**20j** (grey: JMJD5; lavender blue: Mn; Supporting Figure S9) and the reported JMJD5:**1** (teal: JMJD5; pink: Mn; PDB ID: 6I9L[12]) complex structures reveals similar conformations of the side chains of important JMJD5 active site residues. Note that electron density for the indole ring of W248 was not observed in the reported JMJD5:**1** structure. The superimposition implies that both **20j** and **1** bind to JMJD5 in a similar manner; (**c**) superimposition of views from the JMJD5:**20j** (grey: JMJD5; lavender blue: Mn; Supporting Figure S9) and the reported JMJD5:2OG (brown: JMJD5; pink: Mn; PDB ID: 6F4N[13]) complex structures reveals similar JMJD5 conformations (Cα RMSD = 0.271 Å). Note the conformational flexibility of the W248-bearing loop (G240 to W248) in proximity of the C5 substituent of **20j** and that two JMJD5 molecules are present in the asymmetric unit of the reported JMJD5:2OG structure;[13] (**d**) superimposition of views from the active sites of the JMJD5:**20j** (grey: JMJD5; lavender blue: Mn; Supporting Figure S9) and the reported JMJD5:2OG (brown: JMJD5; pink: Mn; PDB ID: 6F4N[13]) complex structures reveals similar conformations of the side chains of important JMJD5 active site residues with the notable exception of W248. The superimposition implies the C2 and C4 carboxylate groups of **20j** interact with JMJD5 in a similar manner as the C1 and C5 carboxylate groups of 2OG and thus that both **20j** and 2OG bind JMJD5 in a similar manner.

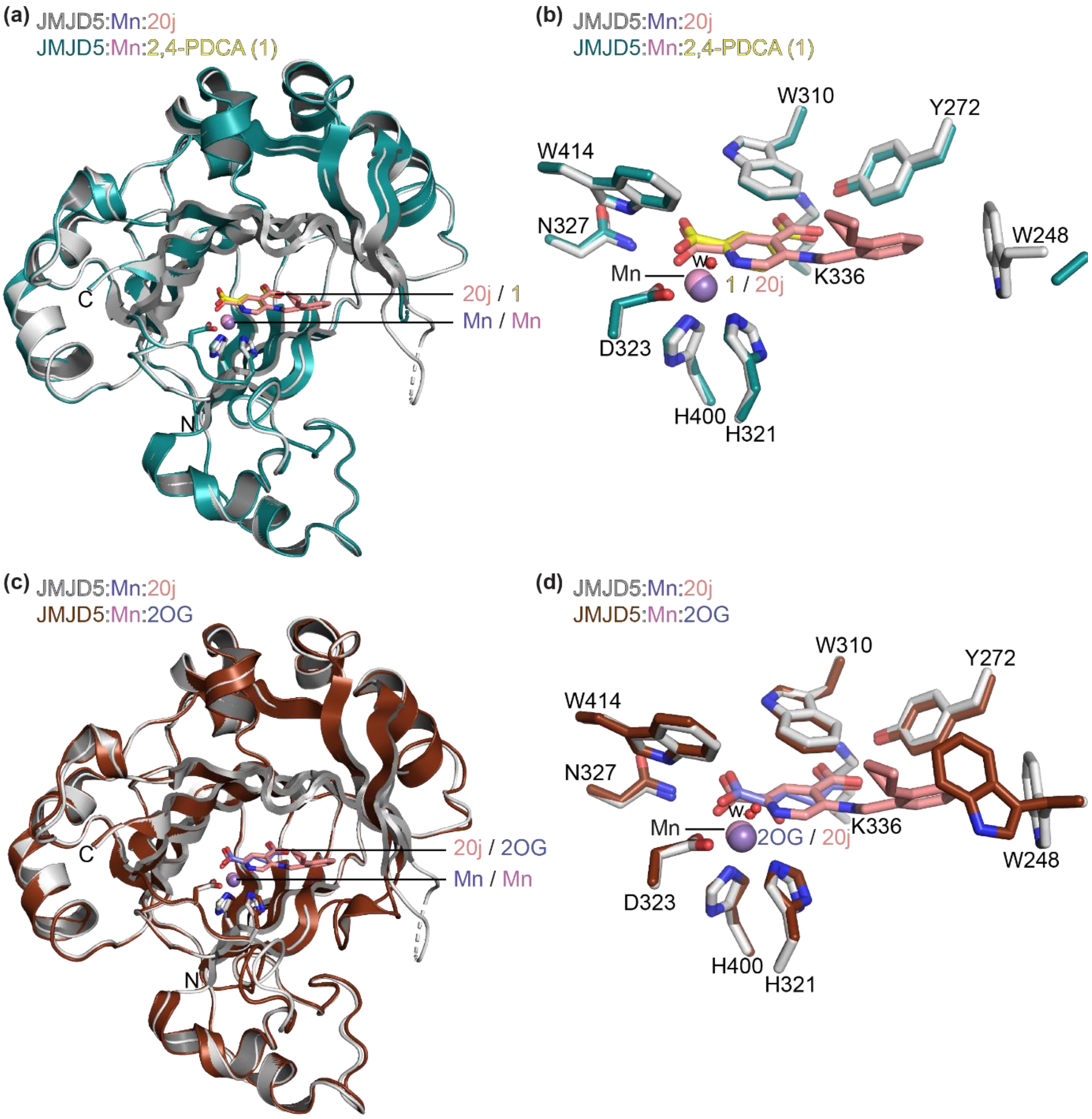
(a) JMJD5:Mn:20j
JMJD5:Mn:2,4-PDCA (1)
C
N
20j / 1
Mn / Mn
(b) JMJD5:Mn:20j
JMJD5:Mn:2,4-PDCA (1)
W310
Y272
W414
N327
W248
Mn
w
1 / 20j
K336
D323
H400
H321
(c) JMJD5:Mn:20j
JMJD5:Mn:2OG
C
N
20j / 2OG
Mn / Mn
(d) JMJD5:Mn:20j
JMJD5:Mn:2OG
W310
Y272
W414
N327
w
Mn
2OG / 20j
K336
W248
D323
H400
H321

Supporting Figure S15. JMJD5 has a similar fold in complex with 5-((2-cyclopropylbenzyl)amino)pyridine-2,4-dicarboxylic acid (20j) and 5-((4-methoxybenzyl)amino)pyridine-2,4-dicarboxylic acid (20h). Colour code: salmon: carbon-backbone of 5-((2-cyclopropylbenzyl)amino)pyridine-2,4-dicarboxylic acid (**20j**); cyan: carbon-backbone of 5-((4-methoxybenzyl)amino)pyridine-2,4-dicarboxylic acid (**20h**); red: oxygen; blue: nitrogen.

a and **b**) Superimposition of views from the JMJD5:**20j** (grey: JMJD5; lavender blue: Mn; Supporting Figure S9) and the JMJD5:**20h** (ochre: JMJD5; pink: Mn; Supporting Figure S7) complex structures reveals (**a**) overall similar JMJD5 conformations (Cα RMSD = 0.149 Å) and (**b**) the conformational flexibility of the W248-bearing loop (G240 to W248) which is part of the β-hairpin motif involving β3 and β4 (T234 to T254) and which is reported to be of importance in modulating substrate binding.[13]

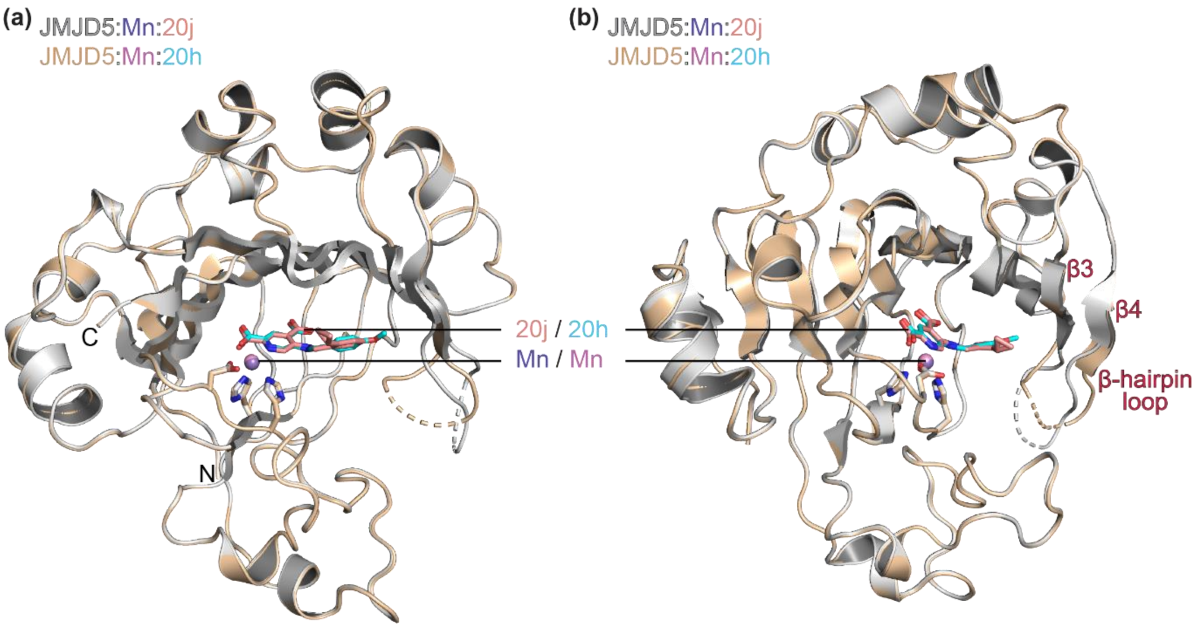

Supporting Figure S16. The C5 substituent of 5-aminoalkyl 2,4-PDCA derivatives extends into the substrate binding pocket of JMJD5. Colour code: salmon: carbon-backbone of 5-((2-cyclopropylbenzyl)amino)pyridine-2,4-dicarboxylic acid (**20j**); olive: carbon-backbone of *N*-oxalylglycine (NOG); red: oxygen; blue: nitrogen.
a) Superimposition of views from the JMJD5:**20j** (grey: JMJD5; lavender blue: Mn; Supporting Figure S9) and the reported JMJD5:NOG:RPS6$_{129-144}$ (pale blue: JMJD5; pink: Mn; lemon: carbon-backbone of R137 of RPS6$_{129-144}$; PDB ID: 6F4P[13]) complex structures reveals similar JMJD5 conformations (Cα RMSD = 0.263 Å); (**b**) superimposition of views from the active sites of the JMJD5:**20j** (grey: JMJD5; lavender blue: Mn; Supporting Figure S9) and the reported JMJD5:NOG:RPS6$_{129-144}$ (pale blue: JMJD5; pink: Mn; lemon: carbon-backbone of R137 of RPS6$_{129-144}$; PDB ID: 6F4P[13]) complex structures reveals similar conformations of the side chains of JMJD5 active site residues which are directly involved in the binding of **20j**, with the notable exception of W248 which is positioned in the JMJD5:**20j** complex structure to interact with the *para* C–H of the **20j** phenyl group through a σ-π interaction. Note that the side chain of W414 was refined in two alternative conformations in the JMJD5:NOG:RPS6$_{129-144}$ complex structure. The superimposition implies that the C5 substituent of **20j** extends into the JMJD5 substrate binding pocket and likely impairs substrate binding.

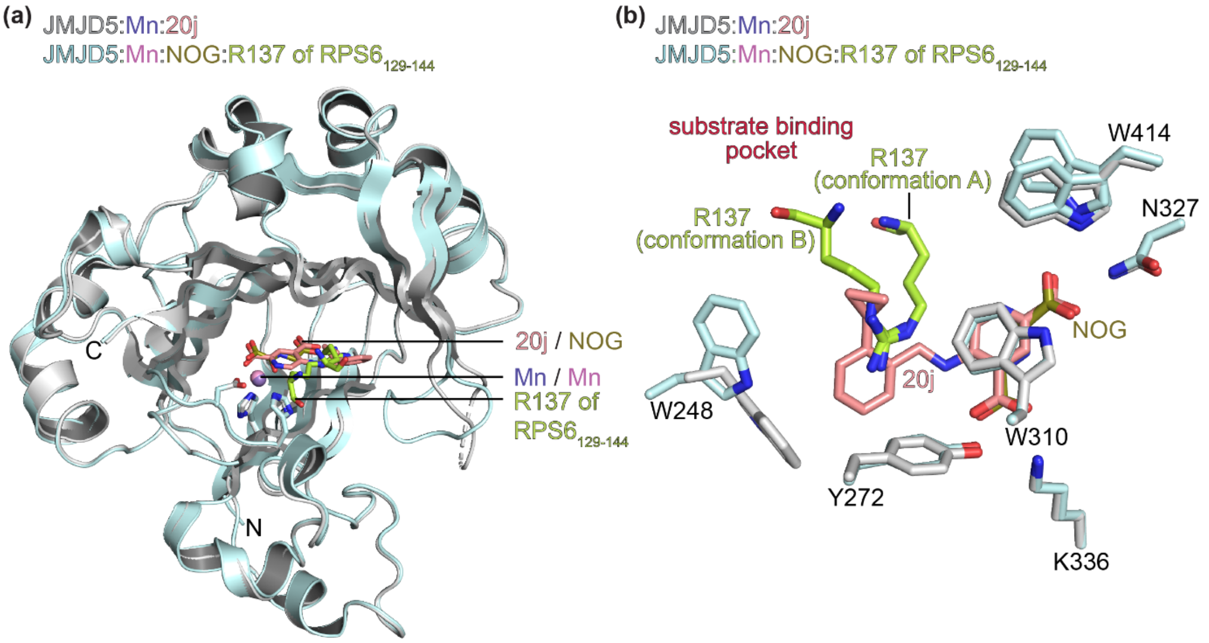

Supporting Figure S17. JMJD5 and FIH share a similar fold but have different substrate binding pockets (continues on the following page). Colour code: salmon: carbon-backbone of 5-((2-cyclopropylbenzyl)amino)pyridine-2,4-dicarboxylic acid (**20j**); yellow: carbon-backbone of 2,4-PDCA (**1**); olive: carbon-backbone of *N*-oxalylglycine (NOG); slate blue: carbon-backbone of 2OG; red: oxygen; blue: nitrogen. w: water.

a) Superimposition of views from the JMJD5:**20j** (grey: JMJD5; lavender blue: Mn; Supporting Figure S9) and the reported FIH:**1** (pale green: FIH; brown: Fe; PDB ID: 2W0X[14]) complex structures reveals a similar fold of JMJD5 and FIH (Cα RMSD = 1.252 Å). However, both the FIH C-terminus and the FIH loop corresponding to the JMJD5 W248-bearing loop (G240 to W248), which is part of the β-hairpin motif involving β3 and β4 (T234 to T254), are extended compared to JMJD5; (**b**) superimposition of views from the active sites of the JMJD5:**20j** (grey: JMJD5; lavender blue: Mn; Supporting Figure S9) and the reported FIH:**1** (pale green: FIH; brown: Fe; PDB ID: 2W0X[14]) complex structures reveals similar conformations of the side chains of JMJD5 and FIH active site residues directly involved in the binding of **20j** or **1**, respectively. Note that W310 of JMJD5 is positioned to interact with the 2,4-PDCA derivatives via π-π stacking with the 2,4-PDCA pyridine ring and that W310 is substituted for a leucine residue in FIH (L188) which cannot engage in an equivalent interaction with **1**. A serine residue (S118) is located at the position in the FIH structure corresponding to W248 in JMJD5; **c**) superimposition of views from the reported JMJD5:NOG:RPS6$_{129-144}$ (pale blue: JMJD5; lavender blue: Mn; lemon: carbon-backbone of R137 of RPS6$_{129-144}$; PDB ID: 6F4P[13]) and the reported FIH:2OG:HIF-1α$_{786-826}$ (pale pink: FIH; brown: Fe; dark yellow: carbon-backbone of HIF-1α$_{786-826}$; PDB ID: 1H2L[15]) complex structures reveals a similar fold of JMJD5 and FIH (Cα RMSD = 1.178 Å). However, both the FIH C-terminus and the FIH loop corresponding to the JMJD5 W248-bearing loop (G240 to W248) which is part of the β-hairpin motif involving β3 and β4 (T234 to T254) are extended compared to JMJD5, which may reduce the accessibility of the active site for substrates; **d**) superimposition of views from the active sites of the reported JMJD5:NOG:RPS6$_{129-144}$ (pale blue: JMJD5; lavender blue: Mn; lemon: carbon-backbone of R137 of RPS6$_{129-144}$; PDB ID: 6F4P[13]) and the reported FIH:2OG:HIF-1α$_{786-826}$ (pale pink: FIH; brown: Fe; dark yellow: carbon-backbone of HIF-1α$_{786-826}$; PDB ID: 1H2L[15]) complex structures reveals differences in substrate binding among JMJD5 and FIH. The loop corresponding to the W248-bearing loop (G240 to W248) in JMJD5, which is part of the β-hairpin motif involving β3 and β4 (T234 to T254), is extended by 15 residues in FIH compared to JMJD5 and. together with the extended C-terminus, apparently shields the FIH active site which may result in the observed different binding geometry of the HIF-1α$_{786-826}$ substrate in the FIH active site compared with the binding geometry of the RPS6$_{129-144}$ substrate in the JMJD5 active site. Note, however, that an accurate comparison of the substrate binding modes of FIH and JMJD5 would require a JMJD5 structure in complex with a larger substrate fragment.

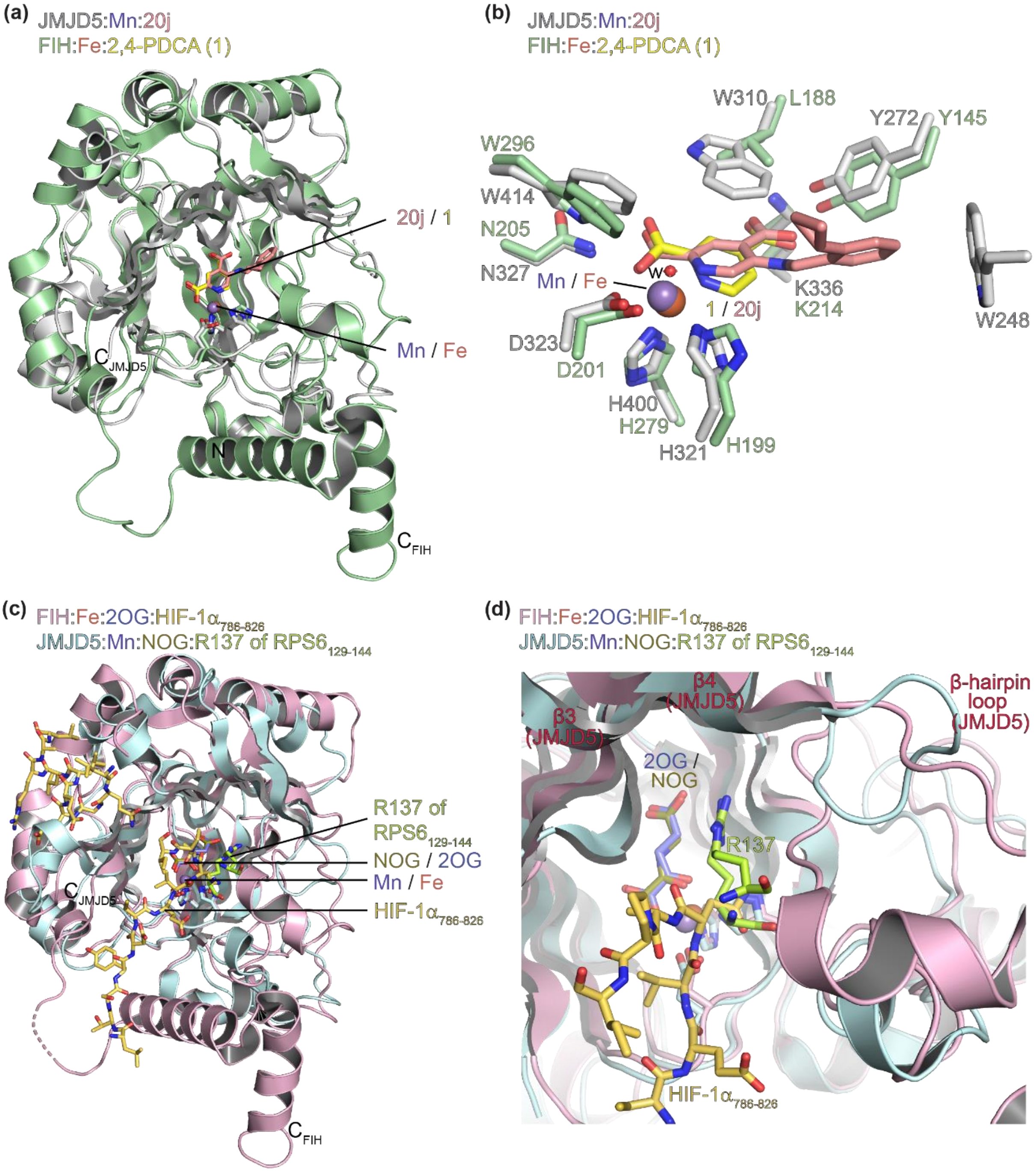
(a)
JMJD5:Mn:20j
FIH:Fe:2,4-PDCA (1)
20j / 1
Mn / Fe
C JMJD5
N
C FIH
(b)
JMJD5:Mn:20j
FIH:Fe:2,4-PDCA (1)
W310
L188
Y272
Y145
W296
W414
N205
N327
Mn / Fe
w
1 / 20j
K336
K214
W248
D323
D201
H400
H279
H321
H199
(c)
FIH:Fe:2OG:HIF-1α 786-826
JMJD5:Mn:NOG:R137 of RPS6 129-144
R137 of RPS6 129-144
NOG / 2OG
Mn / Fe
HIF-1α 786-826
C JMJD5
C FIH
(d)
FIH:Fe:2OG:HIF-1α 786-826
JMJD5:Mn:NOG:R137 of RPS6 129-144
β4 (JMJD5)
β3 (JMJD5)
β-hairpin loop (JMJD5)
2OG / NOG
R137
HIF-1α 786-826

Supporting Figure S18. The fold of JMJD5 in complex with 5-((2-cyclopropylbenzyl)amino)pyridine-2,4-dicarboxylic acid is similar to that of both KDM4E and the AspH oxygenase domain in complex with 2,4-PDCA. Colour code: salmon: carbon-backbone of 5-((2-cyclopropylbenzyl)amino)pyridine-2,4-dicarboxylic acid (**20j**); yellow: carbon-backbone of 2,4-PDCA (**1**); red: oxygen; blue: nitrogen. w: water.

a) Superimposition of views from the JMJD5:**20j** (grey: JMJD5; lavender blue: Mn; Supporting Figure S9) and the reported KDM4E:**1** (pale pink: JMJD5; green: Ni; PDB ID: 2W2I[10]) complex structures reveals a similar fold of JMJD5 and KDM4E (Cα RMSD = 1.104 Å). Note that three KDM4E molecules are present in the asymmetric unit of the reported KDM4E:**1** complex structure;[10] (**b**) superimposition of views from the JMJD5:**20j** (grey: JMJD5; lavender blue: Mn; Supporting Figure S9) and the reported AspH:**1**:hFX-EGFD$_{86\text{-}124}$ (pale blue: AspH; pink: Mn; violet: carbon-backbone of hFX-EGFD$_{86\text{-}124}$; PDB ID: 5JTC[16]) complex structures reveals a similar fold of JMJD5 and the oxygenase domain of AspH (Cα (oxygenase domain of AspH) RMSD = 3.69 Å); note that the crystallized AspH construct comprises both the catalytic oxygenase domain and the adjacent tetratricopeptide repeat (TPR) domain.

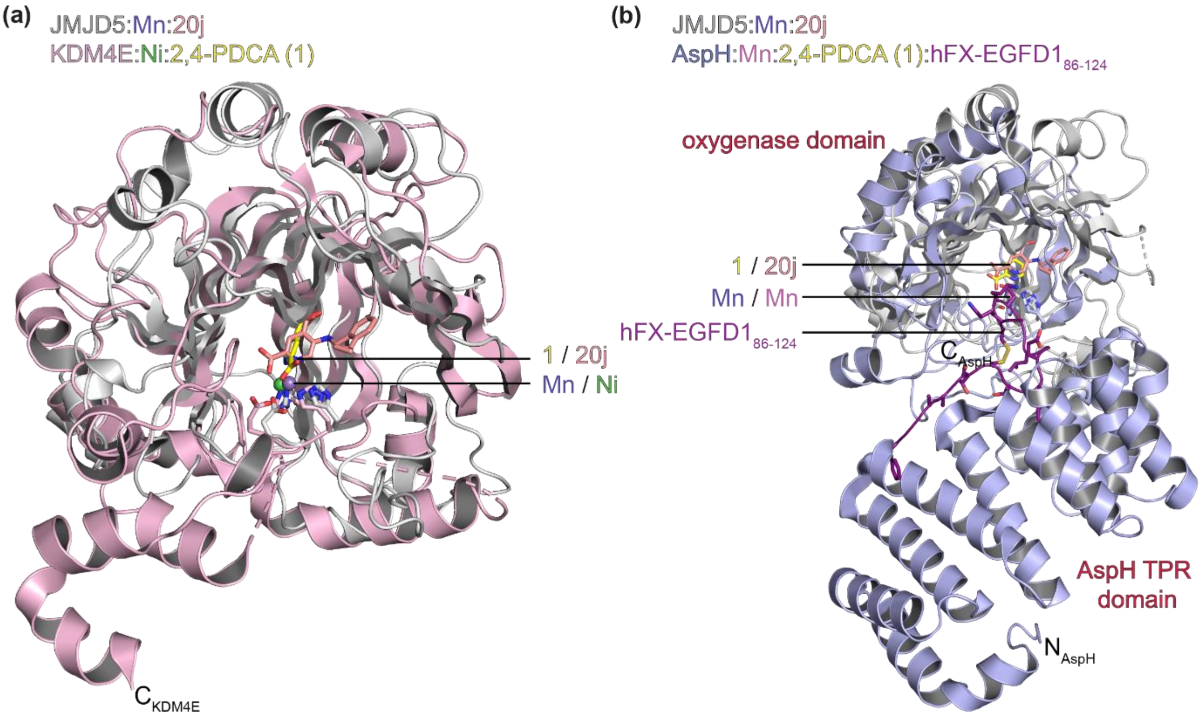

Supporting Figure S19. The 5-aminoalkyl-substituted 2,4-PDCA derivatives 19i and 19j cause reduced viability of U2OS bone osteosarcoma cells. U2OS cells were treated with the indicated doses of 2,4-PDCA derivatives **19i** and **19j** (1.0, 5.0, 10, 50, 100 μM) for 72 h and then tested for viability using the CyQuant assay, which measures DNA content. Data represent mean ± SEM from independent experiments.

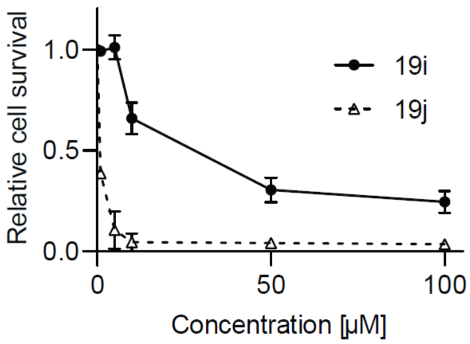

2. Supporting tables

Supporting Table S1. 2,4-PDCA derivatives inhibit human JMJD5 (continues on the following page). SPE-MS JMJD5 inhibition assays were performed as described using isolated recombinant JMJD5 (0.15 μM), 2OG (2.0 μM), Fe(II) (2.0 μM), LAA (100 μM), and $RPS6_{128\text{-}148}$ (2.0 μM) in buffer (50 mM MOPS, pH 7.5, 20 °C),[1] representative dose-response curves are shown in Supporting Figure S1. The 2,4-PDCA derivatives **2-16** and **21-31** were synthesized as described.[17-19]

All 3-substituted 2,4-PDCA derivatives investigated for JMJD5 inhibition were less potent than 2,4-PDCA (**1**). In general, the tested 3-aminoaryl substituted 2,4-PDCA derivatives were relatively inefficient JMJD5 inhibitors (*i.e.* IC_{50} > 20 μM), with the exception of **2** (IC_{50} ~ 16.8 μM; Entry F). Note that **2** inhibited JMJD5 and AspH with approximately similar potency, indicating that substituents on the 2,4-PDCA scaffold have the potential to alter the selectivity profile of **1** considering that **1** inhibits JMJD5 ~15-fold less efficiently than AspH (Entry A). By contrast to the tested 3-aminoaryl substituted 2,4-PDCA derivatives, some of the corresponding 3-aminoalkyl-substituted 2,4-PDCA derivatives inhibited JMJD5 more efficiently (*i.e.* IC_{50} ≲ 10 μM), in particular the 3-aminobenzyl-substituted 2,4-PDCA derivatives **3** and **6-9** (Entries L, O, P, Q, S). However, even the most potent JMJD5 inhibitor identified from this set of 2,4-PDCA derivatives, *i.e.* racemic **8**, inhibited ~5-fold less efficiently than 2,4-PDCA (IC_{50} ~ 2.6 μM; Entry Q).

3-Fluoro-2,4-PDCA (**13**), 3-trifluoromethyl-2,4-PDCA (**14**), and 5-trifluoromethyl-2,4-PDCA (**16**) did not efficiently inhibit JMJD5 (Entries Y, Z, and AB), whereas the inhibition potency observed for 5-fluoro-2,4-PDCA (**15**) was similar to that of the 3-aminobenzyl-substituted 2,4-PDCA derivatives **3** and **6-9** (IC_{50} ~ 4.5 μM; Entry AA). The dimethyl ester derivative of **1** does not inhibit isolated recombinant JMJD5 efficiently (Entry AC).

	2,4-PDCA derivative	[a]IC_{50} JMJD5 [μM]	[b]IC_{50} KDM4E [μM]	[c]IC_{50} AspH [μM]		2,4-PDCA derivative	[a]IC_{50} JMJD5 [μM]	[b]IC_{50} KDM4E [μM]	[c]IC_{50} AspH [μM]
A	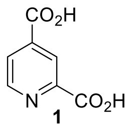 **1**	0.5 ± 0.2	0.3 ± 0.1	0.03 ± 0.01[16]	F	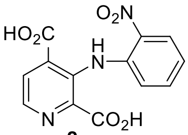 **2**	17.9 ± 2.6	56[17]	16.1 ± 4.0[18]
B	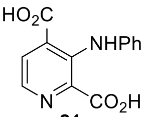 **21**	>50	19[17]	38.8 ± 7.0[18]	G	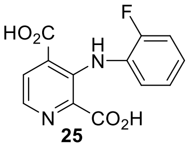 **25**	46.9 ± 4.6	2.5[17]	22.1 ± 1.5[18]
C	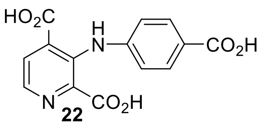 **22**	>50	66[17]	inactive [18]	H	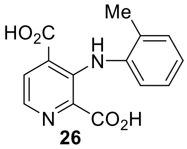 **26**	>50	41[17]	12.9 ± 1.5[18]
D	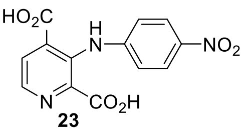 **23**	>50	95[17]	inactive [18]	I	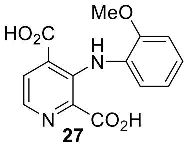 **27**	29.6 ± 4.3	19[17]	13.8 ± 0.2[18]
E	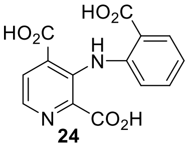 **24**	>50	166[17]	10.4 ± 1.8[18]	J	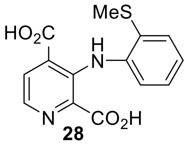 **28**	41.5 ± 2.2	48[17]	16.1 ± 2.5[18]

	2,4-PDCA derivative	[a]IC50 JMJD5 [μM]	[b]IC50 KDM4E [μM]	[c]IC50 AspH [μM]		2,4-PDCA derivative	[a]IC50 JMJD5 [μM]	[b]IC50 KDM4E [μM]	[c]IC50 AspH [μM]
K	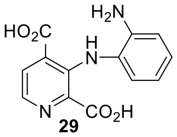 **29**	39.6 ± 11.2	0.43 ± 0.05[18]	4.67 ± 0.29[18]	U	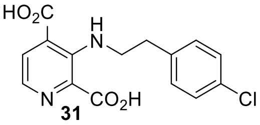 **31**	10.3 ± 3.1	4.14 ± 0.27[18]	3.67 ± 0.74[18]
L	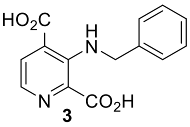 **3**	9.5 ± 2.2	2.36 ± 0.13[18]	3.95 ± 0.33[18]	V	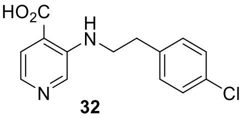 **32**	>50	not reported	not reported
M	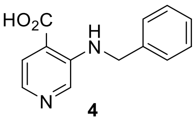 **4**	>50	not reported	not reported	W	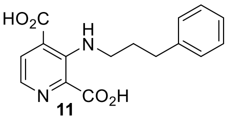 **11**	19.7 ± 3.0	not reported	10.91 ± 1.83[18]
N	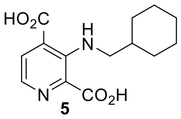 **5**	25.3 ± 0.5	not reported	13.13 ± 4.84[18]	X	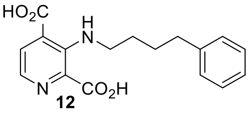 **12**	20.8 ± 0.2	not reported	10.76 ± 0.28[18]
O	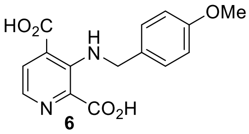 **6**	5.8 ± 2.9	2.45 ± 0.59[18]	0.58 ± 0.06[18]	Y	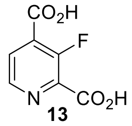 **13**	27.2 ± 2.9	1.3 ± 0.2[19]	0.11 ± 0.01[19]
P	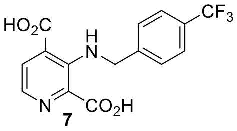 **7**	10.4 ± 4.4	3.57 ± 0.38[18]	1.86 ± 1.06[18]	Z	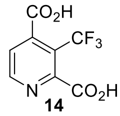 **14**	>50	inactive[19]	>50[19]
Q[d]	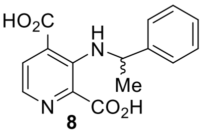 **8**	3.1 ± 1.5	0.99 ± 0.31[18]	0.28 ± 0.11[18]	AA	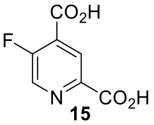 **15**	4.5 ± 0.9	1.6 ± 0.3[19]	0.05 ± 0.01[19]
R[d]	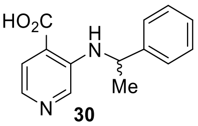 **30**	>50	not reported	not reported	AB	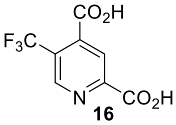 **16**	>50	inactive[19]	4.2 ± 1.0[19]
S[d]	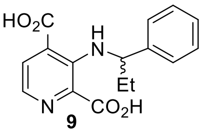 **9**	7.5 ± 2.0	3.69 ± 0.85[18]	1.22 ± 0.26[18]	AC	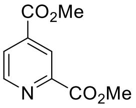 **2,4-PDCA dimethyl ester**	>50	not reported	not reported
T	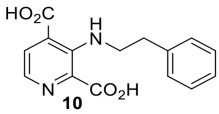 **10**	19.6 ± 3.0	5.71 ± 0.04[18]	7.66 ± 1.45[18]					

a) Determined using reported SPE-MS inhibition assays,[1] data are a mean of three independent runs (n = 3; mean ± standard deviation, SD). Pyridine carboxylates were synthesized as reported;[17-19] b) reported IC_{50} values of the 2,4-PDCA derivatives were either obtained using a formaldehyde dehydrogenase (FDH)-coupled, spectrophotometric KDM4E inhibition assays[17] or using SPE-MS KDM4E inhibition assays;[18-19] c) reported IC_{50} values of the 2,4-PDCA derivatives were obtained using SPE-MS AspH inhibition assays;[18-19] d) chiral 2,4-PDCA derivatives were prepared as racemic mixtures.

Supporting Table S2. Selected crystallographic data for 2,4-PDCA derivatives 18 and 19a.

Compound	**18**	**19a**
CCDC	2266944	2266945
Empirical formula	C9 H8 Cl N O4	C16 H16 N2 O4
Formula weight	229.62	300.31
Crystal system	orthorhombic	monoclinic
Space group	P n a 2_1	I 2/a
Unit cell dimensions	a = 38.7697(6) Å α= 90°	a = 28.9645(14) Å α= 90°
	b = 3.82160(10) Å β= 90°	b = 9.6931(7) Å β= 103.108(5)°
	c = 6.54010(10) Å γ = 90°	c = 42.381(2) Å γ = 90°
Volume	969.00(3) Å^3	11588.7(12) Å^3
Z	4	32
Density (calculated)	1.574 Mg/m^3	1.377 Mg/m^3
F(000)	472	5056
Reflections collected	17727	24646
Independent reflections	2022 [R(int) = 0.039]	12006 [R(int) = 0.053]
Data / restraints / parameters	2020 / 1 / 137	12005 / 0 / 793
Goodness-of-fit on F^2	1.0000	1.0011
Final R indices [I > 2σ(I)]	R1 = 0.0331, wR2 = 0.0906	R1 = 0.0566, wR2 = 0.1272
R indices (all data)	R1 = 0.0336, wR2 = 0.0909	R1 = 0.1164, wR2 = 0.1698
Absolute structure parameter	0.007(5)	-

Supporting Table S3. Crystallization conditions, data collection, and refinement statistics for the JMJD5:2,4-PDCA derivative complexes.[a)]

	JMJD5·Mn^{II}·**20h** (JMJD5:**20h**)	JMJD5·Mn^{II}·**20d** (JMJD5:**20d**)	JMJD5·Mn^{II}·**20a** (JMJD5:**20a**)	JMJD5·Mn^{II}·**20i** (JMJD5:**20i**)	JMJD5·Mn^{II}·**20j** (JMJD5:**20j**)
**PDB ID**	7DYT	7DYU	7DYV	7DYW	7DYX
**Data collection**					
Space group	$P2_12_12_1$	$P2_12_12_1$	$P2_12_12_1$	$P2_12_12_1$	$P2_12_12_1$
Symmetry	orthorhombic	orthorhombic	orthorhombic	orthorhombic	orthorhombic
Cell dimensions:					
a, *b*, *c* (Å)	49.35, 65.00, 78.61	49.37, 64.56, 78.49	49.35, 65.30, 78.63	49.64, 65.42, 79.57	48.92, 64.75, 78.65
α, β, γ (°)	90.00, 90.00, 90.00	90.00, 90.00, 90.00	90.00, 90.00, 90.00	90.00, 90.00, 90.00	90.00, 90.00, 90.00
X-Ray source[b)]	Synchrotron (DLS I24)	Synchrotron (DLS I24)	Synchrotron (DLS I03)	Synchrotron (ESRF ID30A-1)	Synchrotron (ESRF ID30A-1)
Resolution (Å)[c)]	50.09-1.62 (1.65-1.62)	49.86-1.72 (1.85-1.72)	50.25-1.92 (1.95-1.92)	42.12-2.13 (2.26-2.13)	48.92-2.27 (2.41-2.27)
R_{merge}	0.161 (1.117)	0.155 (1.805)	0.711 (1.277)	0.202 (2.864)	0.435 (2.966)
$I / \sigma I$	11.2 (1.1)	11.9 (1.2)	3.4 (0.8)	6.0 (0.4)	2.7 (0.4)
CC (1/2)	0.996 (0.451)	0.996 (0.351)	0.821 (0.196)	0.991 (0.129)	0.943 (0.098)
Total number of reflections	313972	258193	223985	76417	50356
Total number unique reflections	30389	20810	19900	27195	21676
Completeness (%)	92.5 (59.2) [d)]	94.2 (58.5) [d)]	99.3 (99.6)	97.3 (92.6)	97.1 (98.2)
Multiplicity	10.3	12.4	11.3	2.8	2.3
**Refinement**					
R_{work} / R_{free}	0.163 / 0.189	0.160 / 0.195	0.204 / 0.240	0.214 / 0.239	0.242 / 0.280
No. atoms:	4350	4117	4174	4159	4044
B-factors:	28.0	33.0	41.0	57.0	53.0
R.m.s. deviations:					
Bond lengths (Å)	0.005	0.017	0.003	0.012	0.004
Bond angles (°)	0.763	1.271	0.588	0.940	0.583

[a)]Experimental details are specified in the Experimental Section; [b)]DLS: Diamond Light Source, ESRF: The European Synchrotron Radiation Facility; [c)]Values in parentheses are for highest-resolution shell; [d)]Ellipsoidal completeness, as defined by autoPROC/STARANISO (Experimental Section).

3. General information

Unless otherwise stated, all reagents were from commercial sources (Sigma-Aldrich, Inc.; Fluorochem Ltd) and used as received. 2,4-PDCA derivatives **2**, **3**, **5-16**, **21-29**, and **31** were synthesized as reported.[17-19] Anhydrous solvents were from Sigma-Aldrich, Inc. and kept under an atmosphere of nitrogen. Solvents, liquids, and solutions were transferred using nitrogen-flushed stainless-steel needles and syringes. Milli-Q® Ultrapure (MQ-grade) water was used for buffers; LCMS grade solvents (Merck) were used for solid phase extraction coupled to mass spectrometry (SPE-MS).

Purifications were performed using an automated Biotage Isolera One purification machine (wavelength monitored: 254 and 280 nm) equipped with pre-packed Biotage® SNAP KP-Sil or Biotage® SNAP Ultra flash chromatography cartridges. The cartridge size and solvent gradients (in column volumes, CV) used, are specified in the individual experimental procedures. HPLC grade solvents (ethyl acetate and cyclohexane; Sigma-Aldrich Inc.) were used for reaction work-ups, extractions, and purifications.

Thin layer chromatography (TLC) was carried out using Merck silica gel 60 F_{254} TLC plates and visualized under UV light. Melting points (m.p.) were determined using a Stuart SMP-40 automated melting point apparatus. Infrared (IR) spectroscopy was performed using a Bruker Tensor-27 Fourier transform infrared (FT-IR) spectrometer. High-resolution mass spectrometry (HRMS) was performed using electro-spray ionization (ESI) mass spectrometry (MS) in the positive or negative ionization modes employing a Thermo Scientific Exactive mass spectrometer (ThermoFisher Scientific); data are presented as a mass-to-charge ratio (*m/z*).

Single crystal X-ray diffraction data were collected using an Oxford Diffraction SuperNova diffractometer (Rigaku). Structures were solved using SUPERFLIP software[20] and refined using the CRYSTALS software suite[21-22]. Crystallographic data can be obtained from the Cambridge Crystallographic Data Centre (CCDC 2266944 for **18**; CCDC 2266945 for **19a**).

Nuclear magnetic resonance (NMR) spectroscopy was performed using a Bruker AVANCE AVIIIHD 600 machine equipped with a 5mm BB-F/1H Prodigy N_2 cryoprobe. Chemical shifts for protons are reported in parts per million (ppm) downfield from tetramethylsilane and are referenced to residual protium in the NMR solvent ($CDCl_3$: δ = 7.28 ppm; D_2O: δ = 4.79 ppm). For ^{13}C NMR, chemical shifts are reported in the scale relative to the NMR solvent (*i.e.* $CDCl_3$: δ = 77.00 ppm). For ^{19}F NMR, chemical shifts are reported in the scale relative to $CFCl_3$.

NMR data are reported as follows: chemical shift, multiplicity (s: singlet, d: doublet, dd: doublet of doublets, t: triplet, q: quartet, m: multiplet, br: broad signal), coupling constant (*J*, Hz; accurate to 0.1 Hz), and integration. ^{13}C NMR chemical shift numbers in brackets indicate close signals that can be differentiated by considering second decimal numbers.

4. General synthetic procedures

4.1 General Procedure A

To cesium carbonate (1.4 equiv.), palladium acetate (0.04 equiv.), and 4,5-bis(diphenylphosphino)-9,9-dimethylxanthene (Xantphos,[23] 0.06 equiv.) in a capped 20 mL microwave reaction vial were added sequentially anhydrous toluene (0.25 M), methyl 3-bromopyridine-4-carboxylate (1.0 equiv.), and an *N*-alkylamine (1.2 equiv.) at ambient temperature. Nitrogen gas was bubbled through the reaction mixture for 15 min, the reaction mixture was then placed into a preheated sand bath (150 °C) and stirred overnight (14–18 h) under a nitrogen atmosphere. The reaction mixture was then cooled to ambient temperature, concentrated, and purified by column chromatography to afford the desired purified methyl 5-aminoalkyl pyridine-4-carboxylate which was used in the next reaction according to General Procedure C.

4.2 General Procedure B

To dimethyl 5-chloropyridine-2,4-dicarboxylate **18** (1.0 equiv.), palladium acetate (0.04 equiv.), and (*R*)-1-[(S_P)-2-(dicyclohexylphosphino)ferrocenyl]ethyldi-*tert*-butylphosphine (Josiphos SL-J009-1)[24-25] (0.06 equiv.; note that the commercially-sourced enantiopure Josiphos SL-J009-1 ligand was used for this achiral transformation) in a capped 20 mL Biotage microwave reaction vial were added sequentially anhydrous 1,4-dioxane (0.07 M), *N*,*N*-diisopropylethylamine (3.0 equiv),[26] and an *N*-alkylamine (3.0 equiv.) at ambient temperature. Nitrogen gas was bubbled through the reaction mixture for 15 min, the reaction mixture was then placed into a preheated sand bath (170 °C) and stirred for 24 h under a nitrogen atmosphere. The reaction mixture was cooled to ambient temperature, concentrated, and purified by column chromatography to afford the desired purified dimethyl 5-aminoalkyl pyridine-2,4-dicarboxylate which was used in the next reaction according to General Procedure C.

4.3 General Procedure C

A procedure reported for the synthesis of C3 substituted pyridine-2,4-dicarboxylate derivatives was followed:[18-19] To a solution of a 5-substituted dimethyl pyridine-2,4-dicarboxylate (1.0 equiv.) in methanol (0.2 M, HPLC grade) was added an aqueous solution of lithium hydroxide (0.4 M, 2.8 equiv.) under an ambient atmosphere at 0 °C. The reaction mixture was slowly warmed to ambient temperature overnight (14 – 18 h). The methanol was then removed under reduced pressure and the remaining aqueous reaction mixture was extracted three times with dichloromethane (the organic extracts were discarded). The aqueous phase was acidified (pH ≈ 7.0 to 7.7) using Dowex® 50XW8 (H^+-form, mesh 200–400), filtered, and lyophilized to afford the solid C5 substituted pyridine-2,4-dicarboxylate. The crude product was sufficiently pure as judged by ^{1}H and ^{13}C NMR and used without further purification in the biological assays. pKa-values for the 2,4-PDCA derivatives were not determined, thus, some might have been isolated as the corresponding mono- or dilithium salts; note that some 2,4-PDCA derivatives contain trace amounts of acetic acid, as confirmed by ^{1}H and ^{13}C NMR analysis, which has likely been introduced into the samples during lyophilization.

5. Synthetic procedures and analytical data

Methyl 3-(benzylamino)isonicotinate (33).

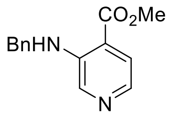

According to General Procedure A, methyl ester **33** (321 mg, 66%) was obtained from commercially-sourced methyl 3-bromopyridine-4-carboxylate (432 mg, 2.0 mmol) and benzylamine, following column chromatography (25 g Ultra cartridge; 50 mL/min; initially, 100% cyclohexane (3 column volumes, CV), followed by a linear gradient (15 CV): 0%→20% ethyl acetate in cyclohexane).

Brown solid, m.p.: 86−88 °C; ^{1}H NMR (600 MHz, 300 K, $CDCl_3$): δ = 8.23 (s, 1H), 7.94 (d, J = 5.1 Hz, 1H), 7.83 (s, 1H), 7.65 (d, J = 5.1 Hz, 1H), 7.40–7.34 (m, 4H), 7.32–7.29 (m, 1H), 4.55 (d, J = 5.7 Hz, 2H), 3.92 ppm (s, 3H); ^{13}C NMR (150 MHz, 300 K, $CDCl_3$): δ = 168.0, 144.6, 138.0, 136.4, 136.3, 128.8, 127.5, 127.1, 123.0, 115.2, 52.1, 46.9 ppm; IR (film): ṽ = 3393, 2944, 1689, 1568, 1444, 1307, 1221, 1176 cm^{-1}; HRMS (ESI): *m/z* calculated for $C_{14}H_{15}N_2O_2$ $[M+H]^+$: 243.1128, found: 243.1129.

3-(Benzylamino)isonicotinic acid (4).

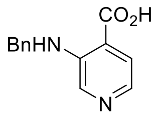

Carboxylic acid **4** (78 mg, 68%) was obtained from methyl 3-(benzylamino)isonicotinate **33** (121 mg, 0.5 mmol) according to General Procedure C. The analytical data of **4** are in agreement with those reported.[27]

Yellow solid, m.p.: >250 °C (decomposition); ^{1}H NMR (600 MHz, 300 K, D_2O): δ = 8.00 (s, 1H), 7.81 (d, J = 5.0 Hz, 1H), 7.54 (d, J = 5.0 Hz, 1H), 7.44–7.33 (m, 4H), 7.32 (s, 1H), 4.49 ppm (d, J = 3.8 Hz, 2H); ^{13}C NMR (150 MHz, 300 K, D_2O): δ = 173.9, 143.7, 139.0, 136.6, 134.9, 128.8, 127.2(2), 127.1(9), 127.0, 124.2, 46.3 ppm; IR (film): ṽ = 3324, 1619, 1574, 1504, 1432, 1385, 1235 cm^{-1}; HRMS (ESI): *m/z* calculated for $C_{13}H_{13}N_2O_2$ $[M+H]^+$: 229.0972, found: 229.0973.

Methyl 2,5-dichloroisonicotinate (34).

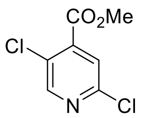

To a solution of commercially-sourced 2,5-dichloroisonicotinic acid (12.1 g, 63.0 mmol, 1.0 equiv.) in anhydrous methanol (100 mL) was added dropwise thionyl chloride (6.9 mL, 94.5 mmol, 1.5 equiv.) at ambient temperature under a nitrogen atmosphere. The reaction mixture was refluxed for 3 h, cooled to ambient temperature, and concentrated. The residue was dissolved in ethyl acetate and washed twice with saturated aqueous $NaHCO_3$ solution and once with brine. The organic phase was dried over anhydrous Na_2SO_4, filtered, evaporated, and purified by column chromatography (100 g KP-Sil cartridge; 80 mL/min; initially, 100% cyclohexane (2 CV), followed by a linear gradient (4 CV): 0%→20% ethyl acetate in cyclohexane) to afford 12.3 g (95%) of purified methyl ester **34**. The analytical data of **34** are in agreement with those reported.[28]

White solid, m.p.: 35−36 °C; ^{1}H NMR (600 MHz, 300 K, $CDCl_3$): δ = 8.51 (s, 1H), 7.72 (s, 1H), 4.00 ppm (s, 3H); ^{13}C NMR (150 MHz, 300 K, $CDCl_3$): δ = 163.3, 150.9, 150.0, 139.2, 129.0, 125.0, 53.2 ppm; IR (film): ṽ = 3087, 3008, 2956, 1744, 1575, 1533, 1448, 1434, 1342, 1323, 1275, 1215, 1138, 1120, 1048, 965 cm^{-1}; HRMS (ESI): *m/z* calculated for $C_7H_6O_2NCl_2$ $[M+H]^+$: 205.9770, found: 205.9772.

Dimethyl 5-chloropyridine-2,4-dicarboxylate (18).

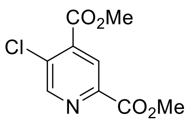

To a solution of methyl 2,5-dichloroisonicotinate **34** (2.06 g, 10 mmol, 1.0 equiv.) and dichloro[2,2′-bis(diphenylphosphino)-1,1′-binaphthyl]palladium(II) [(*rac*-BINAP)$PdCl_2$] (80 mg, 0.1 mmol, 0.01 equiv.) in anhydrous methanol (50 mL) in a 250 mL J-Young Schlenk tube was added *N*,*N*-diisopropylethylamine (2.09 mL, 12 mmol, 1.2 equiv.) at ambient temperature. Carbon monoxide gas (synthesis grade) was bubbled through the solution for 10 minutes. *Caution*: Carbon monoxide is a highly toxic and flammable gas; it should be handled in a well-vented fume cupboard taking

appropriate safety measures. The Schlenk tube was then sealed under CO-pressure (~1.5−2.0 atm) and placed in a sand bath; the tube was then heated with stirring behind a safety shield at 100° C for 20−22 h. The reaction mixture was cooled to ambient temperature, concentrated (^{1}H NMR analysis of the crude reaction mixture indicated an ~8:1 ratio of **18** and trimethyl pyridine-2,4,5-tricarboxylate), and purified by column chromatography (50 g KP-Sil cartridge; 50 mL/min; initially, 100% cyclohexane (3 CV), followed by a linear gradient (15 CV): 0%→25% ethyl acetate in cyclohexane) to afford 1.87 g (81%) of purified dimethyl ester **18**. Single-crystals suitable for X-ray diffraction analysis were obtained from a concentrated solution of **18** in cyclohexane/dichloromethane by slow solvent evaporation at ambient temperature and atmosphere. Data were refined using the full-matrix least-squares on F^2 method, CCDC 2266944 contains the corresponding complete supplementary crystallographic data file, selected crystallographic data are compiled in Supporting Table S2.
White solid, m.p.: 101−102 °C; ^{1}H NMR (600 MHz, 300 K, $CDCl_3$): δ = 8.84 (s, 1H), 8.50 (s, 1H), 4.05 (s, 3H), 4.03 ppm (s, 3H); ^{13}C NMR (150 MHz, 300 K, $CDCl_3$): δ = 164.3, 163.6, 151.5, 146.5, 137.7, 134.1, 125.9, 53.3, 53.2 ppm; IR (film): ṽ = 3098, 3021, 2963, 1743, 1717, 1466, 1444, 1432, 1376, 1313, 1283, 1266, 1227, 1215, 1145, 1126, 1053 cm^{-1}; HRMS (ESI): *m/z* calculated for $C_9H_9O_4NCl$ $[M+H]^+$: 230.0215, found: 230.0217.

Dimethyl 5-(benzylamino)pyridine-2,4-dicarboxylate (19a).

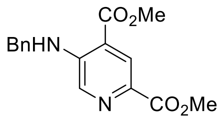

According to General Procedure B, dimethyl ester **19a** (149 mg, 45%) was obtained from dimethyl 5-chloropyridine-2,4-dicarboxylate **18** (230 mg, 1.0 mmol) and commercially sourced benzylamine, following column chromatography (25 g KP-Sil cartridge; 50 mL/min; initially, 100% cyclohexane (3 CV), followed by a linear gradient (15 CV): 0%→40% ethyl acetate in cyclohexane). Single-crystals suitable for X-ray diffraction analysis were obtained from a concentrated solution of analytically pure pyridine **19a** in cyclohexane/dichloromethane by slow solvent evaporation at ambient temperature and atmosphere. Data were refined using the full-matrix least-squares on F^2 method, CCDC 2266945 contains the complete supplementary crystallographic data file for pyridine xx and selected crystallographic data are shown in Supporting Table S2.
Pale yellow solid, m.p.: 93−92 °C; ^{1}H NMR (600 MHz, 300 K, $CDCl_3$): δ = 8.55 (s, 1H), 8.42 (brt, J = 5.5 Hz, 1H), 8.29 (s, 1H), 7.39−7.34 (m, 4H), 7.32−7.30 (m, 1H), 4.61 (d, J = 5.7 Hz, 2H), 3.97 (s, 3H), 3.95 ppm (s, 3H); ^{13}C NMR (150 MHz, 300 K, $CDCl_3$): δ = 167.6, 165.5, 146.7, 137.0, 136.0, 134.4, 129.0, 127.9, 127.2, 126.4, 114.7, 52.4, 52.3, 46.9 ppm; IR (film): ṽ = 3351, 3030, 2952, 1732, 1696, 1574, 1495, 1440, 1410, 1348, 1315, 1280, 1233, 1209, 1130, 1097, 1080, 1064, 990 cm^{-1}; HRMS (ESI): *m/z* calculated for $C_{16}H_{17}O_4N_2$ $[M+H]^+$: 301.1183, found: 301.1183.

5-(Benzylamino)pyridine-2,4-dicarboxylic acid (20a).

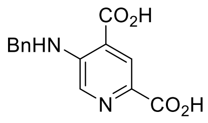

Dicarboxylic acid **20a** (91 mg, 67%) was obtained from dimethyl 5-(benzylamino)pyridine-2,4-dicarboxylate **19a** (149 mg, 0.5 mmol) according to General Procedure C.
Pale yellow solid, m.p.: >345 °C (decomposition); ^{1}H NMR (600 MHz, 300 K, D_2O): δ = 8.21 (s, 1H), 8.02 (s, 1H), 7.48−7.43 (m, 4H), 7.38−7.35 (m, 1H), 4.63 ppm (s, 2H); ^{13}C NMR (150 MHz, 300 K, D_2O): δ = 173.5, 172.2, 145.6, 139.0 (br), 138.7, 133.0 (br), 128.9, 127.4, 127.1, 125.9 (br), 125.4, 46.0 ppm; IR (film): ṽ = 3281, 2924, 2853, 1596, 1497, 1449, 1385, 1300, 1161, 1108 cm^{-1}; HRMS (ESI): *m/z* calculated for $C_{14}H_{11}O_4N_2$ $[M-H]^-$: 271.0724, found: 271.0722.

Dimethyl 5-(2-phenethylamino)pyridine-2,4-dicarboxylate (19b).

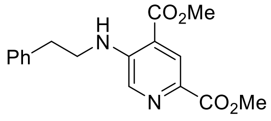

According to General Procedure B, dimethyl ester **19b** (111 mg, 35%) was obtained from dimethyl 5-chloropyridine-2,4-dicarboxylate **18** (229.6 mg, 1.0 mmol) and commercially sourced 2-phenylethylamine, following column chromatography (25 g KP-Sil cartridge; 50 mL/min; initially, 100% cyclohexane (3 CV), followed by a

linear gradient (20 CV): 0%→35% ethyl acetate in cyclohexane).

Pale yellow solid, m.p.: 120−122 °C; ^{1}H NMR (600 MHz, 300 K, $CDCl_3$): δ = 8.53 (s, 1H), 8.34 (s, 1H), 8.05 (brt, J = 4.6 Hz, 1H), 7.38−7.36 (m, 2H), 7.30−7.28 (m, 3H), 3.99 (s, 3H), 3.93 (s, 3H), 3.68−3.65 (m, 2H), 3.04 ppm (t, J = 7.2 Hz, 2H); ^{13}C NMR (150 MHz, 300 K, $CDCl_3$): δ = 167.5, 165.6, 146.7, 138.0, 135.5, 134.0, 128.8, 128.7, 126.9, 126.6, 114.4, 52.4, 52.2, 44.2, 35.5 ppm; IR (film): ṽ = 3346, 3027, 2952, 1731, 1696, 1577, 1495, 1438, 1350, 1315, 1280, 1233, 1128, 1098, 1071, 991 cm^{-1}; HRMS (ESI): *m/z* calculated for $C_{17}H_{19}O_4N_2$ [M+H]$^+$: 315.1339, found: 315.1341.

5-(2-Phenethylamino)pyridine-2,4-dicarboxylic acid (20b).

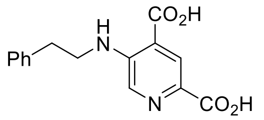

Dicarboxylic acid **20b** (88 mg, 88%) was obtained from dimethyl 5-((2-phenylethyl)amino)pyridine-2,4-dicarboxylate **19b** (111 mg, 0.35 mmol) according to General Procedure C.

Yellow solid, m.p.: >305 °C (decomposition); ^{1}H NMR (600 MHz, 300 K, D_2O): δ = 8.19 (s, 1H), 8.01 (s, 1H), 7.38−7.34 (m, 4H), 7.30−7.27 (m, 1H), 3.62 (t, J = 6.6 Hz, 2H), 3.01 ppm (t, J = 6.5 Hz, 2H); ^{13}C NMR (150 MHz, 300 K, D_2O): δ = 172.8, 170.7 (br), 146.4, 139.4, 136.9 (br), 131.3, 129.1, 128.7, 126.6, 126.4, 125.8, 43.7, 34.7 ppm; HRMS (ESI): *m/z* calculated for $C_{15}H_{15}O_4N_2$ [M+H]$^+$: 287.1026, found: 287.1028.

Dimethyl 5-((3-phenylpropyl)amino)pyridine-2,4-dicarboxylate (19c).

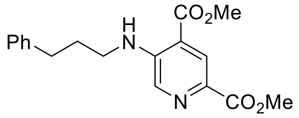

According to General Procedure B, dimethyl ester **19c** (144 mg, 44%) was obtained from dimethyl 5-chloropyridine-2,4-dicarboxylate **18** (229.6 mg, 1.0 mmol) and commercially sourced 3-phenylpropylamine, following column chromatography (25 g KP-Sil cartridge; 50 mL/min; initially, 100% cyclohexane (3 CV), followed by a linear gradient (20 CV): 0%→35% ethyl acetate in cyclohexane).

Clear yellow oil; ^{1}H NMR (600 MHz, 300 K, $CDCl_3$): δ = 8.53 (s, 1H), 8.26 (s, 1H), 8.05 (brt, J = 4.7 Hz, 1H), 7.33−7.30 (m, 2H), 7.24−7.20 (m, 3H), 3.98 (s, 3H), 3.94 (s, 3H), 3.42−3.38 (m, 2H), 2.79 (t, J = 7.5 Hz, 2H), 2.08 ppm (quint., J = 7.3 Hz, 2H); ^{13}C NMR (150 MHz, 300 K, $CDCl_3$): δ = 167.6, 165.6, 146.9, 140.7, 135.5, 133.8, 128.6, 128.3, 126.6, 126.2, 114.1, 52.4, 52.2, 41.8, 33.0, 30.5 ppm; IR (film): ṽ = 3348, 3026, 2950, 2861, 1732, 1694, 1576, 1496, 1437, 1351, 1312, 1280, 1231, 1175, 1128, 1101, 1074, 991, 964 cm^{-1}; HRMS (ESI): *m/z* calculated for $C_{18}H_{21}O_4N_2$ [M+H]$^+$: 329.1496, found: 329.1497.

5-((3-Phenylpropyl)amino)pyridine-2,4-dicarboxylic acid (20c).

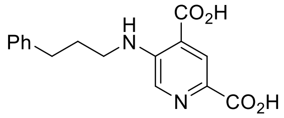

Dicarboxylic acid **20c** (71 mg, 54%) was obtained from dimethyl 5-((3-phenylpropyl)amino)pyridine-2,4-dicarboxylate **19c** (144 mg, 0.44 mmol) according to General Procedure C.

Pale yellow solid, m.p.: >330 °C (decomposition); ^{1}H NMR (600 MHz, 300 K, D_2O): δ = 8.24 (s, 1H), 8.00 (s, 1H), 7.35−7.30 (m, 4H), 7.26−7.23 (m, 1H), 3.35 (t, J = 6.7 Hz, 2H), 2.80 (t, J = 7.4 Hz, 2H), 2.06 ppm (quint., J = 7.0 Hz, 2H); ^{13}C NMR (150 MHz, 300 K, D_2O): δ = 172.5, 169.6 (br), 146.6, 141.9, 135.3 (br), 129.7 (br), 128.6, 128.5, 127.0 (br), 126.2, 126.0, 41.6, 32.3, 29.4 ppm; IR (film): ṽ = 3348, 3215, 2941, 1639, 1598, 1566, 1498, 1453, 1430, 1365, 1294, 1257, 1165, 1107, 1084 cm^{-1}; HRMS (ESI): *m/z* calculated for $C_{16}H_{15}O_4N_2$ [M−H]$^-$: 299.1037, found: 299.1040.

Dimethyl 5-((4-phenylbutyl)amino)pyridine-2,4-dicarboxylate (19d).

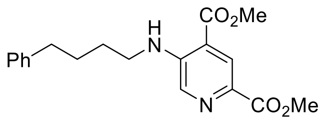

According to General Procedure B, dimethyl ester **19d** (199 mg, 58%) was obtained from dimethyl 5-chloropyridine-2,4-dicarboxylate **18** (229.6 mg, 1.0 mmol) and commercially sourced 4-phenylbutylamine, following column chromatography (25 g KP-Sil cartridge; 50 mL/min; initially, 100% cyclohexane (3 CV), followed by a linear gradient (20 CV): 0%→30% ethyl acetate in cyclohexane).

Clear yellow oil; ^{1}H NMR (600 MHz, 300 K, $CDCl_3$): δ = 8.52 (s, 1H), 8.28 (s, 1H), 7.98 (brt, J = 4.7 Hz, 1H), 7.31−7.29 (m, 2H), 7.22−7.19 (m, 3H), 3.98 (s, 3H), 3.93 (s, 3H), 3.93 (q, J = 6.1 Hz, 2H), 2.70 (t, J = 7.0 Hz, 2H), 1.83−1.76 ppm (m, 4H); ^{13}C NMR (150 MHz, 300 K, $CDCl_3$): δ = 167.6, 165.6, 146.9, 141.7, 135.5, 133.7, 128.4, 128.3, 126.6, 126.0, 114.0, 52.4, 52.2, 42.5, 35.5, 28.6, 28.5(6) ppm; IR (film): ṽ = 3349, 3026, 2949, 2859, 1733, 1695, 1577, 1495, 1438, 1350, 1280, 1233, 1168, 1128, 1076, 991 cm^{-1}; HRMS (ESI): *m/z* calculated for $C_{19}H_{23}O_4N_2$ [M+H]$^+$: 343.1652, found: 343.1652.

5-((4-Phenylbutyl)amino)pyridine-2,4-dicarboxylic acid (20d).

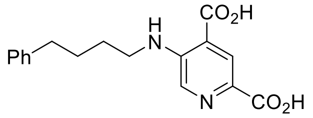

Dicarboxylic acid **20d** (134 mg, 74%) was obtained from dimethyl 5-((4-phenylbutyl)amino)pyridine-2,4-dicarboxylate **19d** (199 mg, 0.58 mmol) according to General Procedure C.

Yellow solid, m.p.: >330 °C (decomposition); ^{1}H NMR (600 MHz, 300 K, D_2O): δ = 8.17 (s, 1H), 8.08 (s, 1H), 7.35 (t, J = 7.5 Hz, 2H), 7.30 (d, J = 7.2 Hz, 2H), 7.24 (t, J = 7.2 Hz, 1H), 3.35 (t, J = 6.6 Hz, 2H), 2.72 (t, J = 7.3 Hz, 2H), 1.81−1.76 (m, 2H), 1.74−1.70 ppm (m, 2H); ^{13}C NMR (150 MHz, 300 K, D_2O): δ = 173.8, 172.8 (br), 145.9, 142.9, 139.0 (br), 133.5, 128.6, 128.5, 125.8, 125.4, 125.0, 42.1, 34.6, 27.9, 27.5 ppm; IR (film): ṽ = 3370, 3220, 3023, 2921, 1650, 1602, 1566, 1496, 1455, 1375, 1297, 1268, 1115, 1090 cm^{-1}; HRMS (ESI): *m/z* calculated for $C_{17}H_{17}O_4N_2$ [M−H]$^-$: 313.1194, found: 313.1196.

***rac*-Dimethyl 5-((1-phenylethyl)amino)pyridine-2,4-dicarboxylate (19e).**

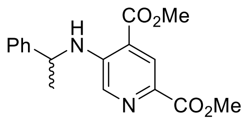

According to General Procedure B, dimethyl ester **19e** (54 mg, 17%) was obtained from dimethyl 5-chloropyridine-2,4-dicarboxylate **18** (229.6 mg, 1.0 mmol) and commercially sourced racemic 1-phenylethylamine, following column chromatography (25 g KP-Sil cartridge; 50 mL/min; initially, 100% cyclohexane (3 CV), followed by a linear gradient (20 CV): 0%→30% ethyl acetate in cyclohexane).

White solid, m.p.: 135−137 °C; ^{1}H NMR (600 MHz, 300 K, $CDCl_3$): δ = 8.52 (s, 1H), 8.42 (brd, J = 5.5 Hz, 1H), 8.11 (s, 1H), 7.35−7.32 (m, 4H), 7.27−7.24 (m, 1H), 4.78 (quint., J = 6.5 Hz, 1H), 3.98 (s, 3H), 3.93 (s, 3H), 1.67 ppm (d, J = 6.7 Hz, 3H); ^{13}C NMR (150 MHz, 300 K, $CDCl_3$): δ = 167.7, 165.5, 145.9, 143.2, 136.9, 134.1, 129.1, 127.6, 126.4, 125.6, 114.5, 53.0, 52.3(5), 52.3, 24.8 ppm; IR (film): ṽ = 3343, 2953, 1734, 1697, 1573, 1493, 1440, 1408, 1347, 1280, 1235, 1201, 1137, 1081, 991 cm^{-1}; HRMS (ESI): *m/z* calculated for $C_{17}H_{19}O_4N_2$ [M+H]$^+$: 315.1339, found: 315.1340.

***rac*-5-((1-Phenylethyl)amino)pyridine-2,4-dicarboxylic acid (20e).**

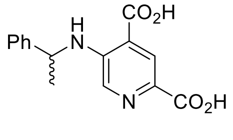

Dicarboxylic acid **20e** (45 mg, 93%) was obtained from *rac*-dimethyl 5-((1-phenylethyl)amino)pyridine-2,4-dicarboxylate **19e** (54 mg, 0.2 mmol) according to General Procedure C.

Pale yellow solid, m.p.: >330 °C (decomposition); ^{1}H NMR (600 MHz, 300 K, D_2O): δ = 8.28 (s, 1H), 7.79 (s, 1H), 7.46 (d, J = 7.4 Hz, 2H), 7.41 (t, J = 7.6 Hz, 2H), 7.32 (t, J = 7.3 Hz, 1H), 4.83−4.80 (m, 1H), 1.61 ppm (d, J = 6.7 Hz, 3H); ^{13}C NMR (150 MHz, 300 K, D_2O): δ = 172.6, 170.0, 145.4, 144.0, 136.6, 131.2, 129.0, 127.5, 127.3, 126.0, 125.9(5), 52.4, 23.8 ppm; IR (film): ṽ = 3231, 3025, 2926, 1601, 1561, 1493, 1443, 1372, 1352, 1267, 1210, 1130 cm^{-1}; HRMS (ESI): *m/z* calculated for $C_{15}H_{13}O_4N_2$ [M−H]$^-$: 285.0881, found:

285.0877.

Dimethyl 5-((cyclohexylmethyl)amino)pyridine-2,4-dicarboxylate (19f).

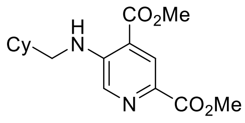

According to General Procedure B, dimethyl ester **19f** (157 mg, 51%) was obtained from dimethyl 5-chloropyridine-2,4-dicarboxylate **18** (229.6 mg, 1.0 mmol) and commercially sourced cyclohexanemethylamine, following column chromatography (25 g KP-Sil cartridge; 50 mL/min; initially, 100% cyclohexane (3 CV), followed by a linear gradient (20 CV): 0%→25% ethyl acetate in cyclohexane).

Pale yellow solid, m.p.: 84−86 °C; ^{1}H NMR (600 MHz, 300 K, $CDCl_3$): δ = 8.52 (s, 1H), 8.29 (s, 1H), 8.09 (brt, J = 4.9 Hz, 1H), 3.98 (s, 3H), 3.94 (s, 3H), 3.22 (t, J = 6.1 Hz, 2H), 1.86 (d, J = 13.0 Hz, 2H), 1.81−1.78 (m, 2H), 1.73−1.66 (m, 2H), 1.33−1.18 (m, 3H), 1.86 ppm (qd, J = 12.2, 3.0 Hz, 2H); ^{13}C NMR (150 MHz, 300 K, $CDCl_3$): δ = 167.8, 165.6, 147.2, 135.7, 133.5, 126.6, 113.8, 52.4, 52.2, 49.1, 37.6, 31.0, 26.3, 25.8 ppm; IR (film): ṽ = 3349, 2925, 2852, 1734, 1694, 1577, 1492, 1439, 1414, 1350, 1313, 1281, 1259, 1230, 1211, 1148, 1127, 1105, 1080, 1068, 992, 962 cm^{-1}; HRMS (ESI): *m/z* calculated for $C_{16}H_{23}O_4N_2$ $[M+H]^+$: 307.1652, found: 307.1650.

5-((Cyclohexylmethyl)amino)pyridine-2,4-dicarboxylic acid (20f).

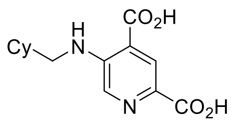

Dicarboxylic acid **20f** (67 mg, 47%) was obtained from dimethyl 5-((cyclohexylmethyl)amino)pyridine-2,4-dicarboxylate **19f** (157 mg, 0.51 mmol) along with some impurities, which could not be further separated, according to General Procedure C.

Yellow solid, m.p.: >285 °C (decomposition); ^{1}H NMR (600 MHz, 300 K, D_2O): δ = 8.21 (s, 1H), 8.10 (s, 1H), 3.16 (t, J = 6.7 Hz, 2H), 1.79 (d, J = 12.5 Hz, 2H), 1.74−1.72 (m, 2H), 1.67−1.65 (m, 2H), 1.29−1.15 (m, 3H), 1.07−1.01 ppm (m, 2H); ^{13}C NMR (150 MHz, 300 K, D_2O): δ = 173.5, 172.0, 146.5, 137.9, 132.7, 125.6, 125.1, 48.9, 36.9, 30.5, 26.0, 25.5 ppm; IR (film): ṽ = 3275, 2922, 2850, 1592, 1499, 1449, 1372, 1325, 1305, 1292, 1268, 1163, 1114, 1068, 1018 cm^{-1}; HRMS (ESI): *m/z* calculated for $C_{14}H_{17}O_4N_2$ $[M-H]^-$: 277.1183, found: 277.1192.

Dimethyl 5-((4-(trifluoromethyl)benzyl)amino)pyridine-2,4-dicarboxylate (19g).

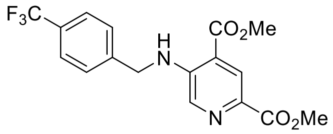

According to General Procedure B, dimethyl ester **19g** (89 mg, 24%) was obtained from dimethyl 5-chloropyridine-2,4-dicarboxylate **18** (229.6 mg, 1.0 mmol) and commercially sourced 4-(trifluoromethyl)benzylamine, following column chromatography (25 g KP-Sil cartridge; 50 mL/min; initially, 100% cyclohexane (3 CV), followed by a linear gradient (20 CV): 0%→35% ethyl acetate in cyclohexane).

White solid, m.p.: 109−111 °C; ^{1}H NMR (600 MHz, 300 K, $CDCl_3$): δ = 8.57 (s, 1H), 8.49 (brt, J = 5.3 Hz, 1H), 8.21 (s, 1H), 7.64 (d, J = 8.1 Hz, 2H), 7.48 (d, J = 8.0 Hz, 2H), 4.69 (d, J = 5.9 Hz, 2H), 3.97 (s, 3H), 3.96(9) ppm (s, 3H); ^{19}F NMR (565 MHz, 300 K, $CDCl_3$): δ = −62.6 ppm (s, 3F); ^{13}C NMR (150 MHz, 300 K, $CDCl_3$): δ = 167.6, 165.4, 146.4, 141.2, 135.8, 135.0, 130.3 (q, J = 32.2 Hz), 127.4, 126.4, 126.0 (q, J = 3.7 Hz), 123.9 (q, J = 272.3 Hz), 115.2, 52.5, 52.4, 46.4 ppm; IR (film): ṽ = 3351, 2955, 1732, 1699, 1619, 1575, 1497, 1440, 1420, 1348, 1326, 1280, 1236, 1210, 1164, 1124, 1068, 1018, 989 cm^{-1}; HRMS (ESI): *m/z* calculated for $C_{17}H_{16}O_4N_2F_3$ $[M+H]^+$: 369.1057, found: 369.1056.

5-((4-(Trifluoromethyl)benzyl)amino)pyridine-2,4-dicarboxylic acid (20g).

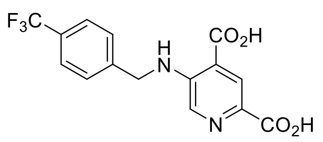

Dicarboxylic acid **20g** (53 mg, 62%) was obtained from dimethyl 5-((4-(trifluoromethyl)benzyl)amino)pyridine-2,4-dicarboxylate **19g** (89 mg, 0.24 mmol) along with some impurities, which could not be further separated, according to General Procedure C.

White solid, m.p.: >325 °C (decomposition); ^{1}H NMR (600 MHz, 300 K, D_2O): δ = 8.19 (s, 1H), 7.97 (s, 1H), 7.73 (d, J = 8.2 Hz, 2H), 7.61 (d, J = 8.1 Hz, 2H), 4.71 ppm (s, 2H); ^{13}C NMR (150 MHz, 300 K, D_2O): δ = 173.9, 173.2, 145.1, 143.4, 140.2, 134.0, 128.6 (q, J = 32.1 Hz), 127.4, 125.6 (q, J = 3.8 Hz), 125.3, 125.2, 124.3 (q, J = 271.3 Hz), 45.6 ppm; IR (film): ṽ = 3274, 2939, 2837, 1596, 1499, 1449, 1419, 1386, 1326, 1163, 1115, 1067, 1018 cm^{-1}; HRMS (ESI): *m/z* calculated for $C_{15}H_{10}O_4N_2F_3$ [M−H]$^{-}$: 339.0598, found: 339.0601.

Dimethyl 5-((4-methoxybenzyl)amino)pyridine-2,4-dicarboxylate (19h).

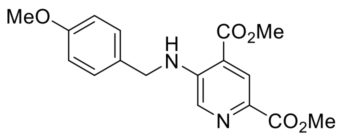

According to General Procedure B, dimethyl ester **19h** (147 mg, 44%) was obtained from dimethyl 5-chloropyridine-2,4-dicarboxylate **18** (229.6 mg, 1.0 mmol) and commercially sourced 4-methoxybenzylamine, following column chromatography (25 g KP-Sil cartridge; 50 mL/min; initially, 100% cyclohexane (3 CV), followed by a linear gradient (20 CV): 0%→30% ethyl acetate in cyclohexane).

Yellow solid, m.p.: 108−110 °C; ^{1}H NMR (600 MHz, 300 K, $CDCl_3$): δ = 8.54 (s, 1H), 8.34−8.31 (m, 2H), 7.29−7.28 (m, 2H), 6.91−6.89 (m, 2H), 4.54 (d, J = 5.6 Hz, 2H), 3.97 (s, 3H), 3.94 (s, 3H), 3.82 ppm (s, 3H); ^{13}C NMR (150 MHz, 300 K, $CDCl_3$): δ = 167.6, 165.6, 159.3, 146.6, 136.0, 134.2, 129.0, 128.6, 126.5, 114.6, 114.4, 55.3, 52.4, 52.3, 46.4 ppm; IR (film): ṽ = 3354, 2999, 2953, 2909, 2838, 1731, 1696, 1613, 1574, 1514, 1494, 1440, 1409, 1349, 1304, 1280, 1234, 1209, 1177, 1130, 1093, 1065, 1033, 990 cm^{-1}; HRMS (ESI): *m/z* calculated for $C_{17}H_{19}O_5N_2$ [M+H]$^{+}$: 331.1288, found: 331.1288.

5-((4-Methoxybenzyl)amino)pyridine-2,4-dicarboxylic acid (20h).

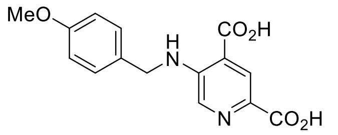

Dicarboxylic acid **20h** (121 mg, 91%) was obtained from dimethyl 5-((4-methoxybenzyl)amino)pyridine-2,4-dicarboxylate **19h** (147 mg, 0.44 mmol) according to General Procedure C.

Yellow solid, m.p.: >300 °C (decomposition); ^{1}H NMR (600 MHz, 300 K, D_2O): δ = 8.20 (s, 1H), 8.02 (s, 1H), 7.39−7.38 (m, 2H), 7.00−6.98 (m, 2H), 4.52 (s, 2H), 3.82 ppm (s, 3H); ^{13}C NMR (150 MHz, 300 K, D_2O): δ = 173.5, 172.2, 158.0, 145.6, 138.9 (br), 133.0, 131.2, 128.6, 125.8, 125.4, 114.3, 55.4, 45.4 ppm; IR (film): ṽ = 3274, 2939, 2836, 1594, 1512, 1447, 1370, 1325, 1301, 1244, 1174, 1114, 1067, 1031, 1019 cm^{-1}; HRMS (ESI): *m/z* calculated for $C_{15}H_{13}O_5N_2$ [M−H]$^{-}$: 301.0830, found: 301.0828.

Dimethyl 5-((2-methoxybenzyl)amino)pyridine-2,4-dicarboxylate (19i).

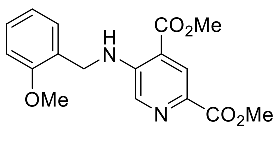

According to General Procedure B, dimethyl ester **19i** (156 mg, 47%) was obtained from dimethyl 5-chloropyridine-2,4-dicarboxylate **18** (229.6 mg, 1.0 mmol) and commercially sourced 2-methoxybenzylamine, following column chromatography (25 g Ultra cartridge; 50 mL/min; initially, 100% cyclohexane (3 CV), followed by a linear gradient (20 CV): 0%→30% ethyl acetate in cyclohexane).

White solid, m.p.: 145−146 °C; ^{1}H NMR (600 MHz, 300 K, $CDCl_3$): δ = 8.55 (s, 1H), 8.52 (brs, 1H), 8.41 (s, 1H), 7.31−7.29 (m, 1H), 7.28−7.26 (m, 1H), 6.94−6.92 (m, 2H), 4.62 (d, J = 6.0 Hz, 2H), 3.98 (s, 3H), 3.95 (s, 3H), 3.91 ppm (s, 3H); ^{13}C NMR (150 MHz, 300 K, $CDCl_3$): δ = 167.3, 164.9, 157.3, 147.1, 135.5, 132.6, 129.2, 128.7, 126.9, 124.8, 120.7, 115.0, 110.6, 55.4, 52.5, 52.3, 42.0 ppm; IR (film): ṽ = 3352, 3000, 2952, 2840, 1696, 1573, 1493, 1438, 1349, 1316, 1280, 1206, 1130, 1090, 1064, 1027, 992 cm^{-1}; HRMS (ESI): *m/z* calculated for $C_{17}H_{19}O_5N_2$ [M+H]$^{+}$: 331.1288, found: 331.1289.

5-((2-Methoxybenzyl)amino)pyridine-2,4-dicarboxylic acid (20i).

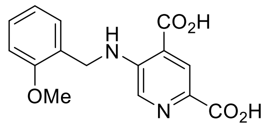

Dicarboxylic acid **20i** (26 mg, 43%) was obtained from dimethyl 5-((2-methoxybenzyl)amino)pyridine-2,4-dicarboxylate **19i** (66 mg, 0.2 mmol) according to General Procedure C.

Yellow solid, m.p.: >260 °C (decomposition); ^{1}H NMR (600 MHz, 300 K, D_2O): δ = 8.19 (s, 1H), 8.11 (s, 1H), 7.40−7.37 (m, 2H), 7.13 (d, J = 8.1 Hz, 1H), 7.03 (t, J = 7.4 Hz, 1H), 4.58 (s, 2H), 3.94 ppm (s, 3H); ^{13}C NMR (150 MHz, 300 K, D_2O): δ = 173.4, 172.2, 157.3, 145.7, 139.0, 133.2, 129.1, 128.8, 126.5, 126.2, 125.4, 120.9, 111.5, 55.6, 41.9 ppm; IR (film): ṽ = 3221, 3066, 2994, 1604, 1572, 1495, 1450, 1382, 1297, 1270 cm^{-1}; HRMS (ESI): *m/z* calculated for $C_{15}H_{13}O_5N_2$ [M−H]$^-$: 301.0830, found: 301.0836.

Dimethyl 5-((2-cyclopropylbenzyl)amino)pyridine-2,4-dicarboxylate (19j).

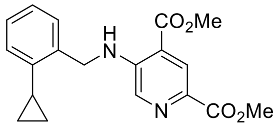

According to General Procedure B, dimethyl ester **19j** (134 mg, 54%) was obtained from dimethyl 5-chloropyridine-2,4-dicarboxylate **18** (168 mg, 0.73 mmol) and commercially sourced 2-cyclopropylbenzylamine, following column chromatography (25 g Ultra cartridge; 50 mL/min; initially, 100% cyclohexane (3 CV), followed by a linear gradient (20 CV): 0%→25% ethyl acetate in cyclohexane).

Pale yellow solid, m.p.: 107−109 °C; ^{1}H NMR (600 MHz, 300 K, $CDCl_3$): δ = 8.58 (s, 1H), 8.42 (brs, 1H), 8.36 (s, 1H), 7.29−7.24 (m, 2H), 7.20−7.17 (m, 1H), 7.12 (d, J = 7.6 Hz, 1H), 4.78 (d, J = 5.4 Hz, 2H), 3.99 (s, 3H), 3.95 (s, 3H), 1.97−1.92 (m, 1H), 1.00−0.97 (m, 2H), 0.75−0.73 ppm (m, 2H); ^{13}C NMR (150 MHz, 300 K, $CDCl_3$): δ = 167.3, 164.9, 146.9, 140.9, 135.8, 135.3, 133.1, 128.1, 127.5, 126.9 (2C), 126.4, 115.1, 52.6, 52.4, 44.9, 12.9, 6.9 ppm; IR (film): ṽ = 3351, 3072, 3001, 2952, 1696, 1573, 1492, 1438, 1348, 1317, 1278, 1232, 1208, 1131, 1088, 1063, 1033, 991 cm^{-1}; HRMS (ESI): *m/z* calculated for $C_{19}H_{21}O_4N_2$ [M+H]$^+$: 341.1496, found: 341.1493.

5-((2-Cyclopropylbenzyl)amino)pyridine-2,4-dicarboxylic acid (20j).

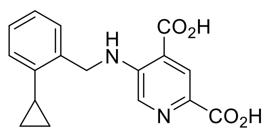

Dicarboxylic acid **20j** (46 mg, 74%) was obtained from dimethyl 5-((2-cyclopropylbenzyl)amino)pyridine-2,4-dicarboxylate **19j** (68 mg, 0.2 mmol) according to General Procedure C.

White solid, m.p.: >320 °C (decomposition); ^{1}H NMR (600 MHz, 300 K, D_2O): δ = 8.19 (s, 1H), 8.08 (s, 1H), 7.42 (d, J = 7.5 Hz, 1H), 7.32 (t, J = 7.5 Hz, 1H), 7.26 (t, J = 7.4 Hz, 1H), 7.19 (d, J = 7.6 Hz, 1H), 2.09−2.05 (m, 1H), 1.04−1.01 (m, 2H), 0.75−0.72 ppm (m, 2H); ^{13}C NMR (150 MHz, 300 K, D_2O): δ = 173.9, 173.3, 145.4, 141.6, 139.9, 137.7, 134.1, 127.8, 127.6, 126.1, 125.9, 125.2, 124.9, 44.3, 12.1, 6.6 ppm; IR (film): ṽ = 3234, 1603, 1571, 1495, 1448, 1381, 1297, 1248 cm^{-1}; HRMS (ESI): *m/z* calculated for $C_{17}H_{17}O_4N_2$ [M+H]$^+$: 313.1183, found: 313.1183.

***rac*-Methyl 3-((1-phenylethyl)amino)isonicotinate (35).**

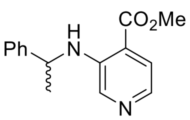

According to General Procedure A, methyl ester **35** (233 mg, 46%) was obtained from commercially-sourced methyl 3-bromopyridine-4-carboxylate (432 mg, 2.0 mmol) and racemic 1-phenylethylamine, following column chromatography (25 g Ultra cartridge; 50 mL/min; initially, 100% cyclohexane (3 CV), followed by a linear gradient (15 CV): 0%→20% ethyl acetate in cyclohexane).

Yellow oil; ^{1}H NMR (600 MHz, 300 K, $CDCl_3$): δ = 8.04 (s, 1H), 7.87 (d, J = 5.1 Hz, 2H), 7.62 (d, J = 5.1 Hz, 1H), 7.38–7.31 (m, 4H), 7.27–7.23 (m, 1H), 4.73–4.69 (m, 1H), 3.95 (s, 3H), 1.62 ppm (d, J = 6.8 Hz, 3H); ^{13}C NMR (150 MHz, 300 K, $CDCl_3$): δ = 168.2, 144.0, 143.9, 137.1, 136.0, 128.9, 127.3, 125.7, 122.9, 115.0, 52.7, 52.0, 25.1 ppm; IR (film): ṽ = 3364, 2953, 1698, 1570, 1499, 1439, 1304, 1226, 1176, 1132 cm^{-1}; HRMS (ESI): *m/z* calculated for $C_{15}H_{17}N_2O_2$ [M+H]$^+$: 257.1285, found: 257.1286.

***rac*-3-((1-Phenylethyl)amino)isonicotinic acid (30).**

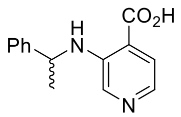

Carboxylic acid **30** (80 mg, 66%) was obtained from *rac*-methyl 3-((1-phenylethyl)amino)isonicotinate **35** (128 mg, 0.5 mmol) according to General Procedure C. The analytical data of **30** are in agreement with those reported.[27]

Yellow solid, m.p.: >250 °C (decomposition); ^{1}H NMR (600 MHz, 300 K, D_2O): δ = 7.87 (s, 1H), 7.76 (d, J = 5.0 Hz, 1H), 7.53 (d, J = 4.9 Hz, 1H), 7.44–7.38 (m, 2H), 7.37–7.33 (m, 2H), 7.26 (t, J = 7.6 Hz, 1H), 4.74 (q, J = 6.7 Hz, 1H), 1.53 ppm (d, J = 6.7 Hz, 3H); ^{13}C NMR (150 MHz, 300 K, D_2O): δ = 173.9, 144.7, 143.2, 136.6, 135.9, 128.9, 127.2, 127.0, 126.0, 124.3, 52.5, 23.9 ppm; IR (film): ṽ = 3292, 1616, 1573, 1503, 1434, 1384, 1248 cm^{-1}; HRMS (ESI): *m/z* calculated for $C_{14}H_{15}N_2O_2$ [M+H]$^+$: 243.1128, found: 243.1129.

Methyl 3-((4-chlorophenethyl)amino)isonicotinate (36).

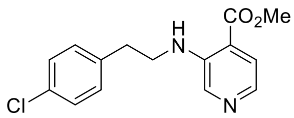

According to General Procedure A, methyl ester **36** (441 mg, 76%) was obtained from commercially-sourced methyl 3-bromopyridine-4-carboxylate (432 mg, 2.0 mmol) and 2-(4-chlorophenyl)ethan-1-amine, following column chromatography (25 g Ultra cartridge; 50 mL/min; initially, 100% cyclohexane (3 CV), followed by a linear gradient (15 CV): 0%→30% ethyl acetate in cyclohexane).

Orange solid, m.p.: 51−53 °C; ^{1}H NMR (600 MHz, 300 K, $CDCl_3$): δ = 8.28 (s, 1H), 7.94 (d, J = 5.1 Hz, 1H), 7.63 (d, J = 5.1 Hz, 1H), 7.41 (d, J = 5.7 Hz, 1H), 7.34–7.29 (m, 2H), 7.23–7.18 (m, 2H), 3.89 (s, 3H), 3.56 (td, J = 7.1, 5.5 Hz, 2H), 2.99 ppm (t, J = 7.1 Hz, 2H); ^{13}C NMR (150 MHz, 300 K, $CDCl_3$): δ = 167.9, 144.6, 137.1, 136.2, 135.7, 132.5, 130.1, 128.8, 123.2, 115.1, 52.0, 43.9, 34.9 ppm; IR (film): ṽ = 3356, 2954, 1689, 1567, 1493, 1439, 1303, 1218, 1168 cm^{-1}; HRMS (ESI): *m/z* calculated for $C_{15}H_{16}N_2O_2Cl$ [M+H]$^+$: 291.0895, found: 291.0895.

3-((4-Chlorophenethyl)amino)isonicotinic acid (32).

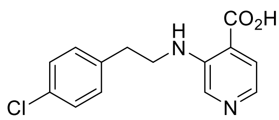

Carboxylic acid **32** (113 mg, 86%) was obtained from methyl 3-(4-chlorophenethyl)isonicotinate **36** (138 mg, 0.47 mmol) according to General Procedure C.

Yellow solid, m.p. >250 °C (decomposition); ^{1}H NMR (600 MHz, 300 K, D_2O): δ = 7.97 (s, 1H), 7.73 (d, J = 5.0 Hz, 1H), 7.41 (d, J = 4.9 Hz, 1H), 7.25–7.19 (m, 2H), 7.17–7.13 (m, 2H), 3.41 (t, J = 6.5 Hz, 2H), 2.84 ppm (t, J = 6.5 Hz, 2H); ^{13}C NMR (150 MHz, 300 K, D_2O): δ = 173.8, 144.1, 137.7, 136.6, 135.0, 132.0, 130.5, 128.4, 126.9, 124.1, 43.9, 34.1 ppm; IR (film): ṽ = 3301, 2980, 1617, 1574, 1504, 1386, 1250, 1091 cm^{-1}; HRMS (ESI): *m/z* calculated for $C_{14}H_{14}N_2O_2Cl$ [M+H]$^+$: 277.0738, found: 277.0739.

6. References

1. Tumber, A.; Salah, E.; Brewitz, L.; Corner, T. P.; Schofield, C. J., Kinetic and inhibition studies on human Jumonji-C (JmjC) domain-containing protein 5. *RSC Chem. Biol.* **2023**, *4*, 399-413.
2. Zhang, J.-H.; Chung, T. D. Y.; Oldenburg, K. R., A simple statistical parameter for use in evaluation and validation of high throughput screening assays. *J. Biomol. Screen.* **1999**, *4* (2), 67-73.
3. Epstein, A. C. R.; Gleadle, J. M.; McNeill, L. A.; Hewitson, K. S.; O'Rourke, J.; Mole, D. R.; Mukherji, M.; Metzen, E.; Wilson, M. I.; Dhanda, A.; Tian, Y.-M.; Masson, N.; Hamilton, D. L.; Jaakkola, P.; Barstead, R.; Hodgkin, J.; Maxwell, P. H.; Pugh, C. W.; Schofield, C. J.; Ratcliffe, P. J., C. elegans EGL-9 and mammalian homologs define a family of dioxygenases that regulate HIF by prolyl hydroxylation. *Cell* **2001**, *107* (1), 43-54.
4. Bruick, R. K.; McKnight, S. L., A conserved family of prolyl-4-hydroxylases that modify HIF. *Science* **2001**, *294* (5545), 1337-1340.
5. Choi, H.; Hardy, A. P.; Leissing, T. M.; Chowdhury, R.; Nakashima, Y.; Ge, W.; Markoulides, M.; Scotti, J. S.; Gerken, P. A.; Thorbjornsrud, H.; Kang, D.; Hong, S.; Lee, J.; McDonough, M. A.; Park, H.; Schofield, C. J., A human protein hydroxylase that accepts *D*-residues. *Commun. Chem.* **2020**, *3* (1), 52.
6. Hewitson, K. S.; McNeill, L. A.; Riordan, M. V.; Tian, Y.-M.; Bullock, A. N.; Welford, R. W.; Elkins, J. M.; Oldham, N. J.; Bhattacharya, S.; Gleadle, J. M.; Ratcliffe, P. J.; Pugh, C. W.; Schofield, C. J., Hypoxia-inducible factor (HIF) asparagine hydroxylase is identical to factor inhibiting HIF (FIH) and is related to the cupin structural family. *J. Biol. Chem.* **2002**, *277* (29), 26351-26355.
7. Stenflo, J.; Holme, E.; Lindstedt, S.; Chandramouli, N.; Tsai Huang, L. H.; Tam, J. P.; Merrifield, R. B., Hydroxylation of aspartic acid in domains homologous to the epidermal growth factor precursor is catalyzed by a 2-oxoglutarate-dependent dioxygenase. *Proc. Natl. Acad. Sci. USA* **1989**, *86* (2), 444-447.
8. Pfeffer, I.; Brewitz, L.; Krojer, T.; Jensen, S. A.; Kochan, G. T.; Kershaw, N. J.; Hewitson, K. S.; McNeill, L. A.; Kramer, H.; Münzel, M.; Hopkinson, R. J.; Oppermann, U.; Handford, P. A.; McDonough, M. A.; Schofield, C. J., Aspartate/asparagine-β-hydroxylase crystal structures reveal an unexpected epidermal growth factor-like domain substrate disulfide pattern. *Nat. Commun.* **2019**, *10* (1), 4910.
9. Brewitz, L.; Onisko, B. C.; Schofield, C. J., Combined proteomic and biochemical analyses redefine the consensus sequence requirement for epidermal growth factor-like domain hydroxylation. *J. Biol. Chem.* **2022**, *298* (8), 102129.
10. Hillringhaus, L.; Yue, W. W.; Rose, N. R.; Ng, S. S.; Gileadi, C.; Loenarz, C.; Bello, S. H.; Bray, J. E.; Schofield, C. J.; Oppermann, U., Structural and evolutionary basis for the dual substrate selectivity of human KDM4 histone demethylase family. *J. Biol. Chem.* **2011**, *286* (48), 41616-41625.
11. Ge, W.; Wolf, A.; Feng, T.; Ho, C.-h.; Sekirnik, R.; Zayer, A.; Granatino, N.; Cockman, M. E.; Loenarz, C.; Loik, N. D.; Hardy, A. P.; Claridge, T. D. W.; Hamed, R. B.; Chowdhury, R.; Gong, L.; Robinson, C. V.; Trudgian, D. C.; Jiang, M.; Mackeen, M. M.; McCullagh, J. S.; Gordiyenko, Y.; Thalhammer, A.; Yamamoto, A.; Yang, M.; Liu-Yi, P.; Zhang, Z.; Schmidt-Zachmann, M.; Kessler, B. M.; Ratcliffe, P. J.; Preston, G. M.; Coleman, M. L.; Schofield, C. J., Oxygenase-catalyzed ribosome hydroxylation occurs in prokaryotes and humans. *Nat. Chem. Biol.* **2012**, *8* (12), 960-962.
12. Islam, M. S.; Markoulides, M.; Chowdhury, R.; Schofield, C. J., Structural analysis of the 2-oxoglutarate binding site of the circadian rhythm linked oxygenase JMJD5. *Sci. Rep.* **2022**, *12* (1), 20680.
13. Wilkins, S. E.; Islam, M. S.; Gannon, J. M.; Markolovic, S.; Hopkinson, R. J.; Ge, W.; Schofield, C. J.; Chowdhury, R., JMJD5 is a human arginyl C-3 hydroxylase. *Nat. Commun.* **2018**, *9* (1), 1180.
14. Conejo-Garcia, A.; McDonough, M. A.; Loenarz, C.; McNeill, L. A.; Hewitson, K. S.; Ge, W.; Liénard, B. M.; Schofield, C. J.; Clifton, I. J., Structural basis for binding of cyclic 2-oxoglutarate analogues to factor-inhibiting hypoxia-inducible factor. *Bioorg. Med. Chem. Lett.* **2010**, *20* (20), 6125-6128.

15. Elkins, J. M.; Hewitson, K. S.; McNeill, L. A.; Seibel, J. F.; Schlemminger, I.; Pugh, C. W.; Ratcliffe, P. J.; Schofield, C. J., Structure of factor-inhibiting hypoxia-inducible factor (HIF) reveals mechanism of oxidative modification of HIF-1α. *J. Biol. Chem.* **2003**, *278* (3), 1802-1806.
16. Brewitz, L.; Tumber, A.; Pfeffer, I.; McDonough, M. A.; Schofield, C. J., Aspartate/asparagine-β-hydroxylase: a high-throughput mass spectrometric assay for discovery of small molecule inhibitors. *Sci. Rep.* **2020**, *10* (1), 8650.
17. Thalhammer, A.; Mecinović, J.; Loenarz, C.; Tumber, A.; Rose, N. R.; Heightman, T. D.; Schofield, C. J., Inhibition of the histone demethylase JMJD2E by 3-substituted pyridine 2,4-dicarboxylates. *Org. Biomol. Chem.* **2011**, *9* (1), 127-135.
18. Brewitz, L.; Tumber, A.; Thalhammer, A.; Salah, E.; Christensen, K. E.; Schofield, C. J., Synthesis of novel pyridine-carboxylates as small-molecule inhibitors of human aspartate/asparagine-β-hydroxylase. *ChemMedChem* **2020**, *15*, 1139-1149.
19. Brewitz, L.; Nakashima, Y.; Tumber, A.; Salah, E.; Schofield, C. J., Fluorinated derivatives of pyridine-2,4-dicarboxylate are potent inhibitors of human 2-oxoglutarate dependent oxygenases. *J. Fluor. Chem.* **2021**, *247*, 109804.
20. Palatinus, L.; Chapuis, G., SUPERFLIP - a computer program for the solution of crystal structures by charge flipping in arbitrary dimensions. *J. Appl. Cryst.* **2007**, *40* (4), 786-790.
21. Parois, P.; Cooper, R. I.; Thompson, A. L., Crystal structures of increasingly large molecules: meeting the challenges with CRYSTALS software. *Chem. Cent. J.* **2015**, *9* (1), 30.
22. Cooper, R. I.; Thompson, A. L.; Watkin, D. J., CRYSTALS enhancements: dealing with hydrogen atoms in refinement. *J. Appl. Cryst.* **2010**, *43* (5), 1100-1107.
23. Kamer, P. C. J.; van Leeuwen, P. W. N. M.; Reek, J. N. H., Wide bite angle diphosphines: Xantphos ligands in transition metal complexes and catalysis. *Acc. Chem. Res.* **2001**, *34* (11), 895-904.
24. Shen, Q.; Shekhar, S.; Stambuli, J. P.; Hartwig, J. F., Highly reactive, general, and long-lived catalysts for coupling heteroaryl and aryl chlorides with primary nitrogen nucleophiles. *Angew. Chem. Int. Ed.* **2005**, *44* (9), 1371-1375.
25. Shen, Q.; Ogata, T.; Hartwig, J. F., Highly reactive, general and long-lived catalysts for palladium-catalyzed amination of heteroaryl and aryl chlorides, bromides, and iodides: Scope and structure–activity relationships. *J. Am. Chem. Soc.* **2008**, *130* (20), 6586-6596.
26. Hünig, S.; Kiessel, M., Spezifische Protonenacceptoren als Hilfsbasen bei Alkylierungs- und Dehydrohalogenierungsreaktionen. *Chem. Ber.* **1958**, *91* (2), 380-392.
27. Westaway, S. M.; Preston, A. G. S.; Barker, M. D.; Brown, F.; Brown, J. A.; Campbell, M.; Chung, C.-w.; Diallo, H.; Douault, C.; Drewes, G.; Eagle, R.; Gordon, L.; Haslam, C.; Hayhow, T. G.; Humphreys, P. G.; Joberty, G.; Katso, R.; Kruidenier, L.; Leveridge, M.; Liddle, J.; Mosley, J.; Muelbaier, M.; Randle, R.; Rioja, I.; Rueger, A.; Seal, G. A.; Sheppard, R. J.; Singh, O.; Taylor, J.; Thomas, P.; Thomson, D.; Wilson, D. M.; Lee, K.; Prinjha, R. K., Cell penetrant inhibitors of the KDM4 and KDM5 families of histone lysine demethylases. 1. 3-Amino-4-pyridine carboxylate derivatives. *J. Med. Chem.* **2016**, *59* (4), 1357-1369.
28. Norcott, P.; Burns, M. J.; Rayner, P. J.; Mewis, R. E.; Duckett, S. B., Using ^{2}H labelling to improve the NMR detectability of pyridine and its derivatives by SABRE. *Magn. Reson. Chem.* **2018**, *56* (7), 663-671.

7. ^{1}H and ^{13}C NMR spectra of C5 substituted 2,4-PDCA derivatives prepared for this study

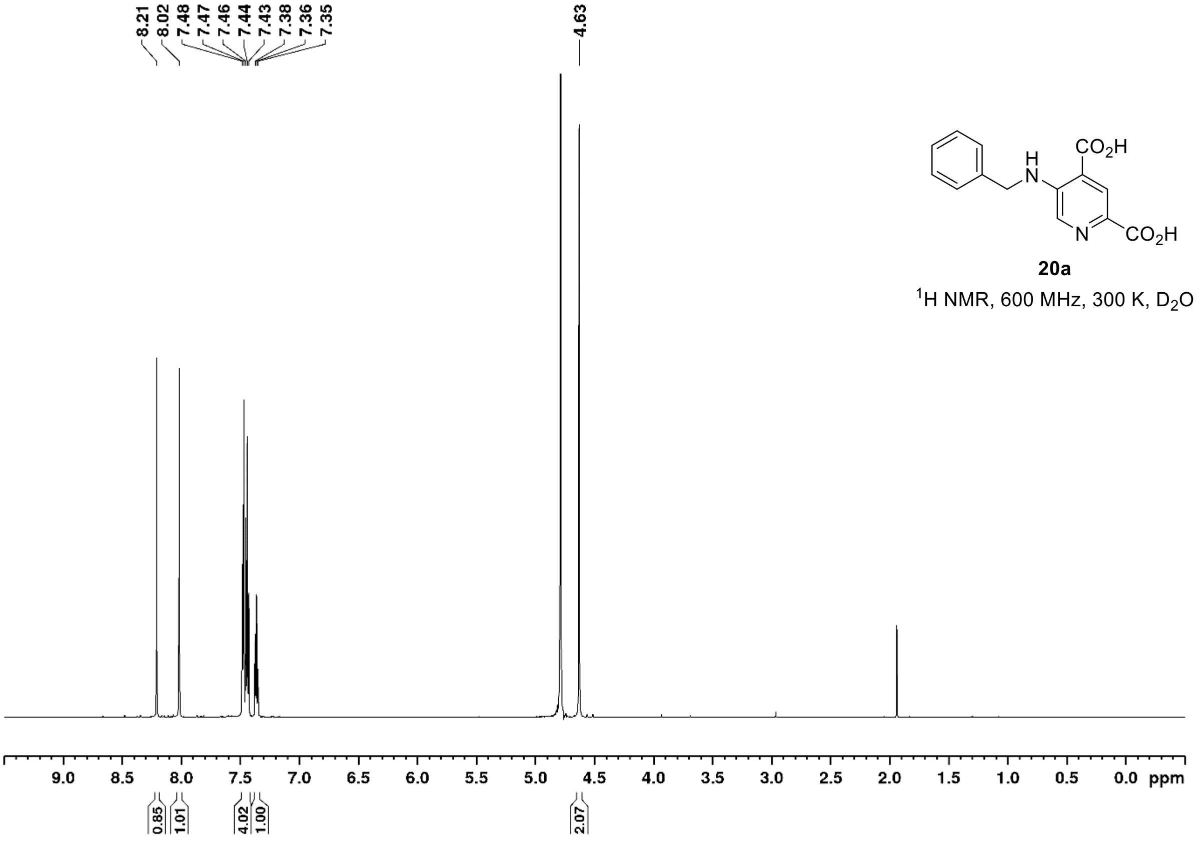

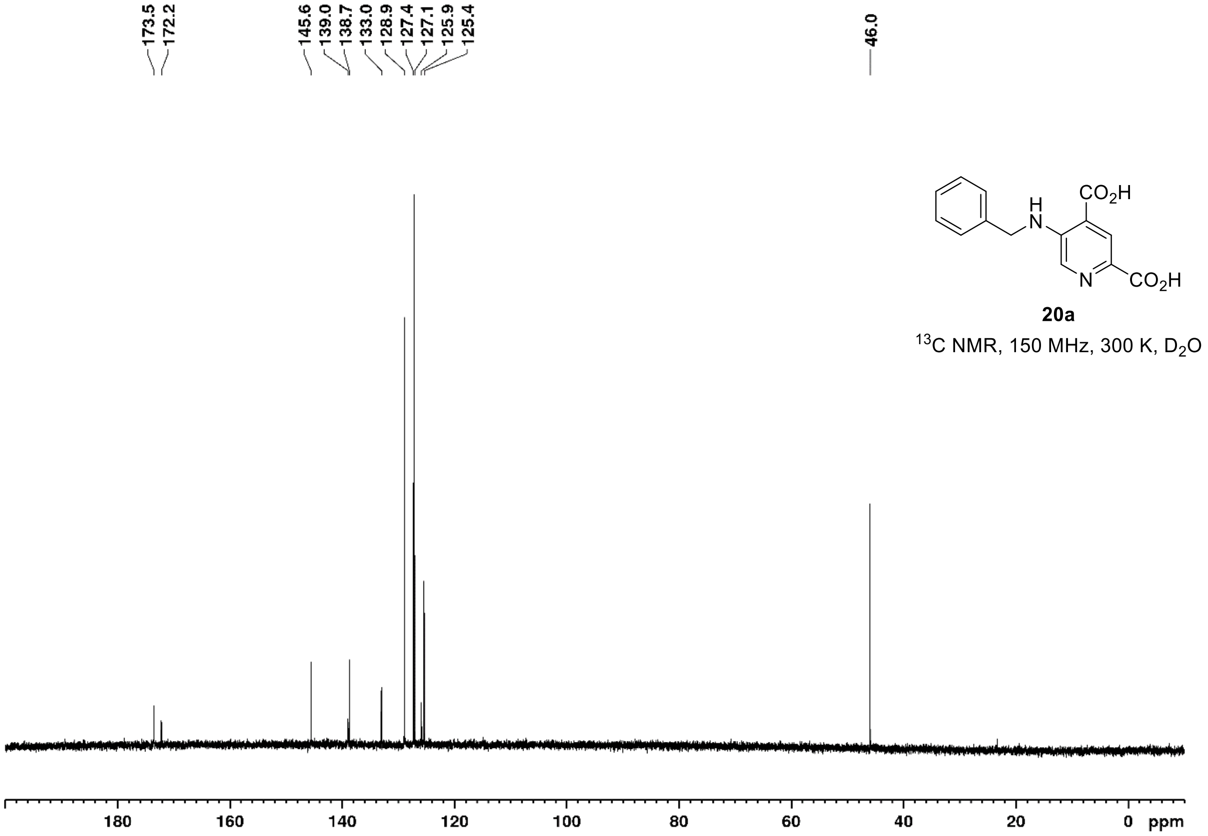

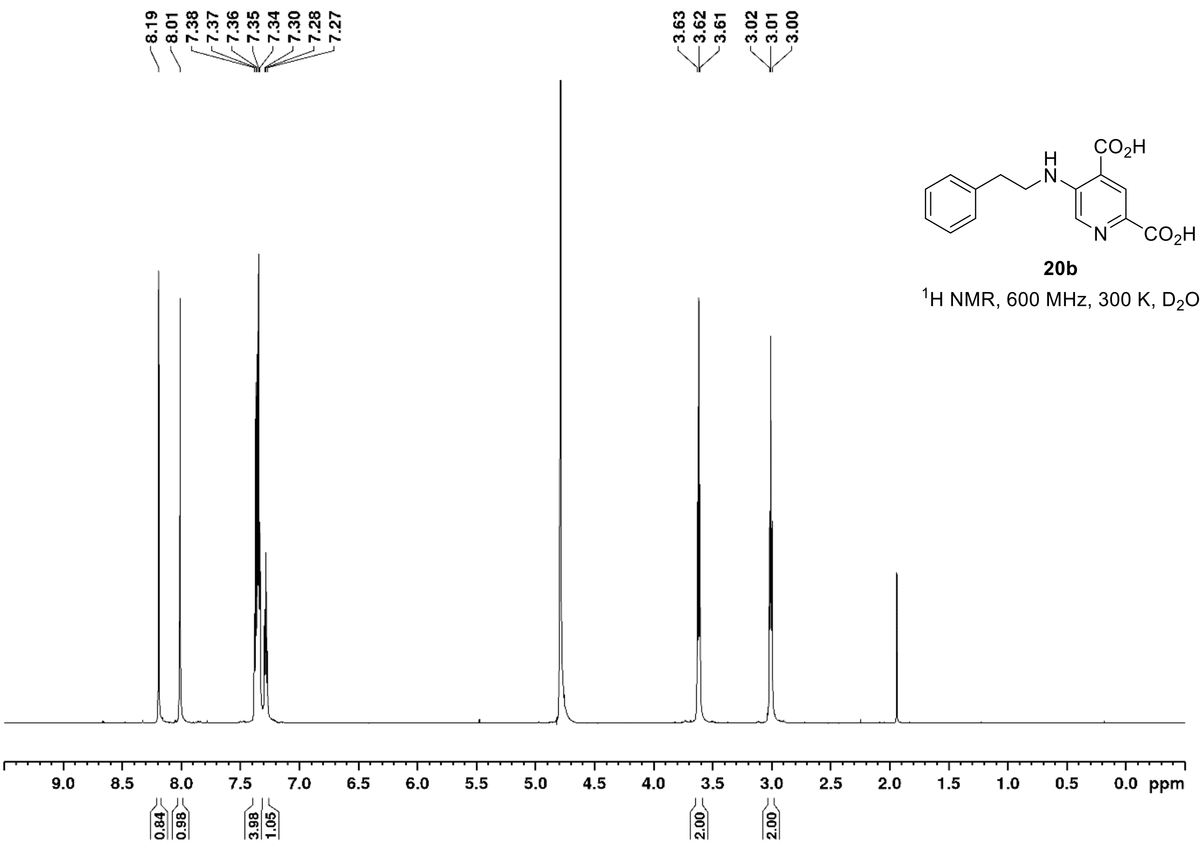
8.19
8.01
7.38
7.37
7.36
7.35
7.34
7.30
7.28
7.27
3.63
3.62
3.61
3.02
3.01
3.00
CO2H
H
N
N
CO2H
20b
1H NMR, 600 MHz, 300 K, D2O
9.0 8.5 8.0 7.5 7.0 6.5 6.0 5.5 5.0 4.5 4.0 3.5 3.0 2.5 2.0 1.5 1.0 0.5 0.0 ppm
0.84
0.98
3.98
1.05
2.00
2.00

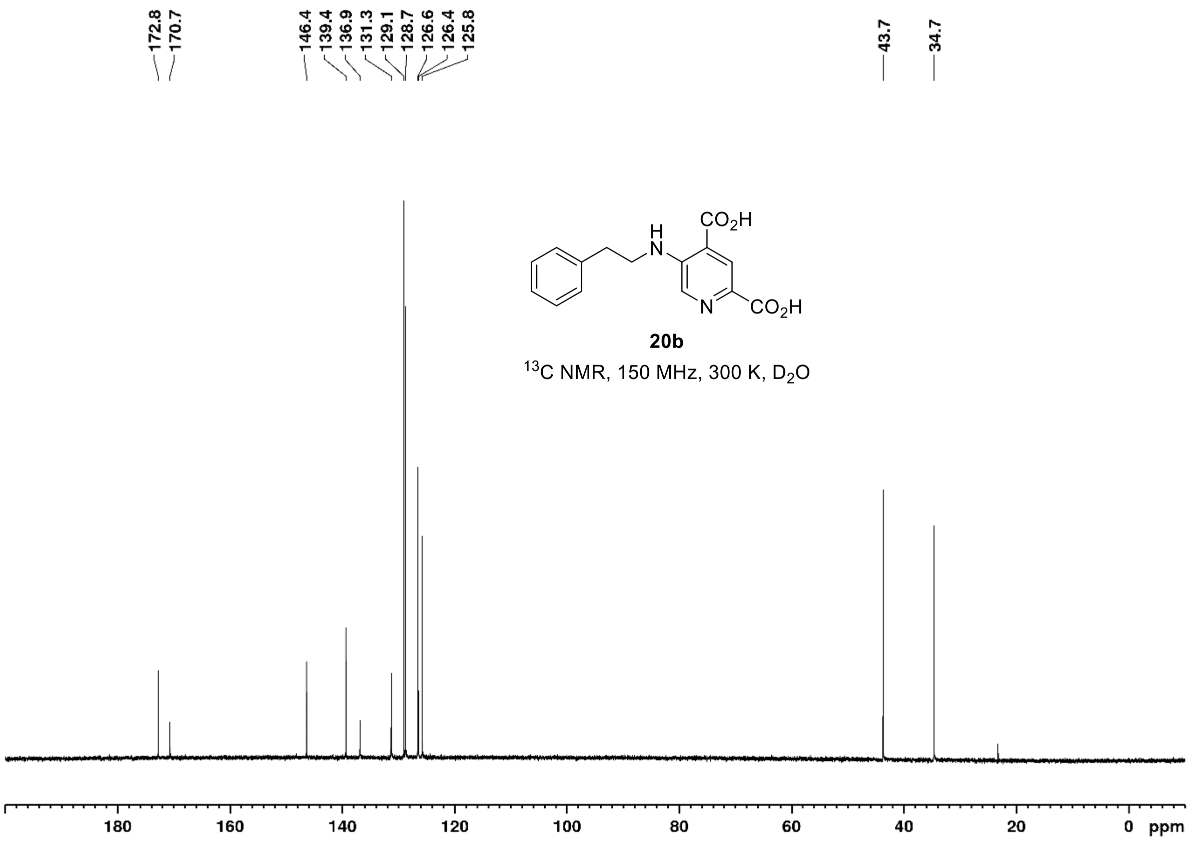
172.8
170.7
146.4
139.4
136.9
131.3
129.1
128.7
126.6
126.4
125.8
43.7
34.7
CO2H
H
N
N
CO2H
20b
13C NMR, 150 MHz, 300 K, D2O
180 160 140 120 100 80 60 40 20 0 ppm

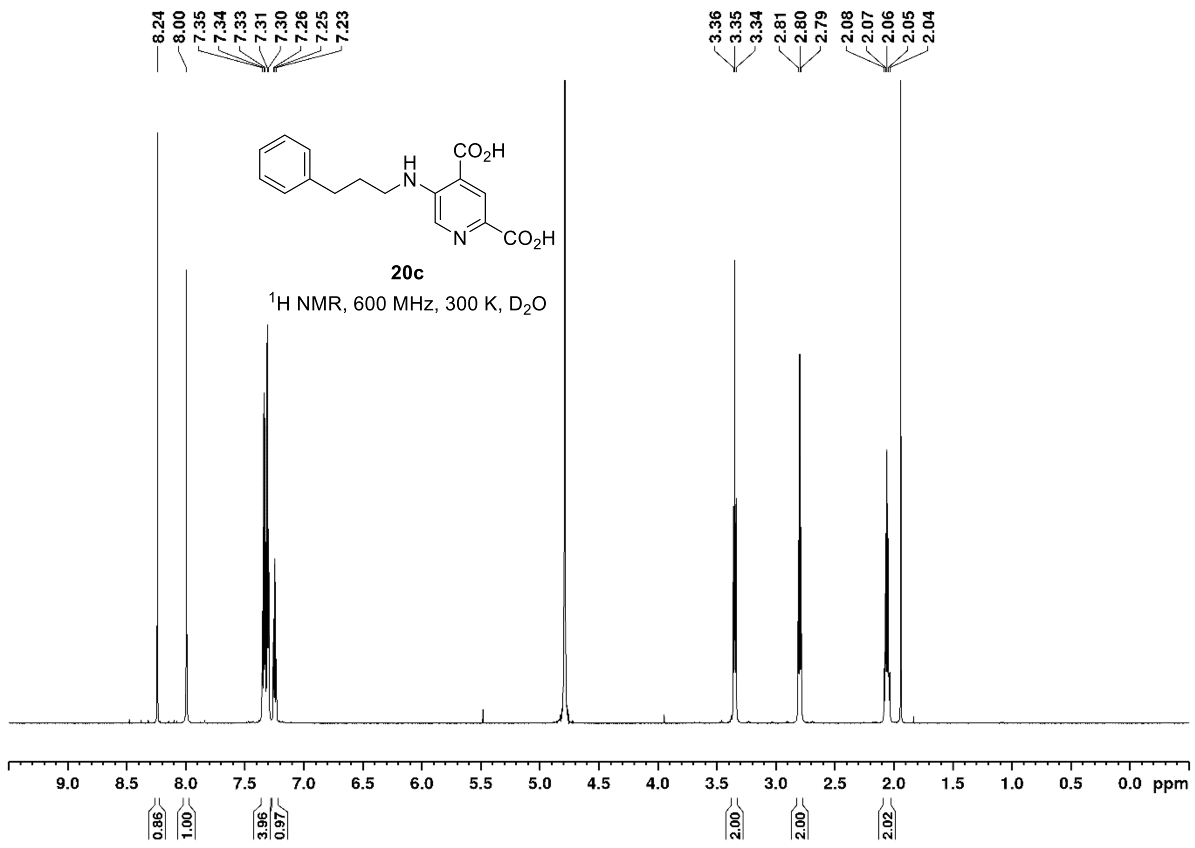
8.24
8.00
7.35
7.34
7.33
7.31
7.30
7.26
7.25
7.23
3.36
3.35
3.34
2.81
2.80
2.79
2.08
2.07
2.06
2.05
2.04
H
N
CO_2H
N
CO_2H
20c
^1H NMR, 600 MHz, 300 K, D_2O
9.0 8.5 8.0 7.5 7.0 6.5 6.0 5.5 5.0 4.5 4.0 3.5 3.0 2.5 2.0 1.5 1.0 0.5 0.0 ppm
0.86
1.00
3.96
0.97
2.00
2.00
2.02

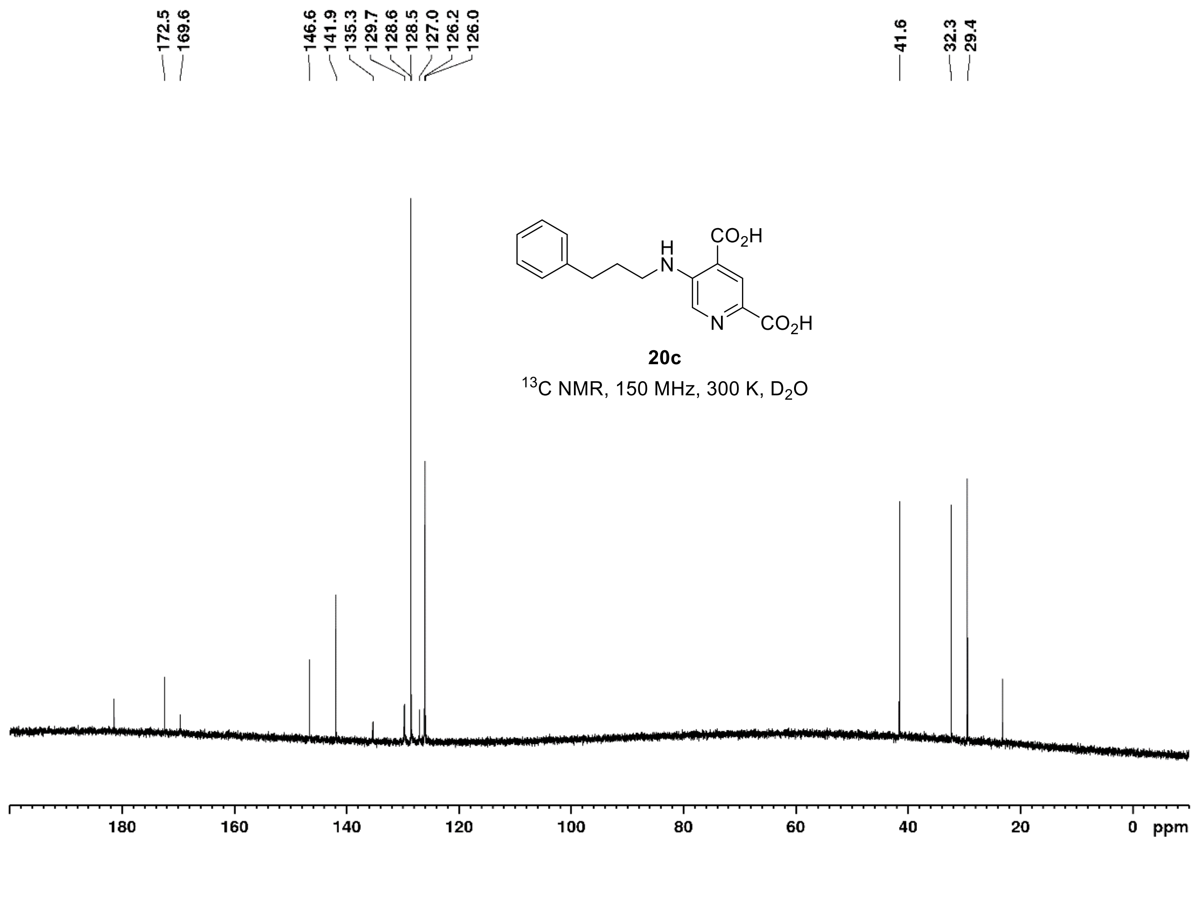
172.5
169.6
146.6
141.9
135.3
129.7
128.6
128.5
127.0
126.2
126.0
41.6
32.3
29.4
H
N
CO_2H
N
CO_2H
20c
^{13}C NMR, 150 MHz, 300 K, D_2O
180 160 140 120 100 80 60 40 20 0 ppm

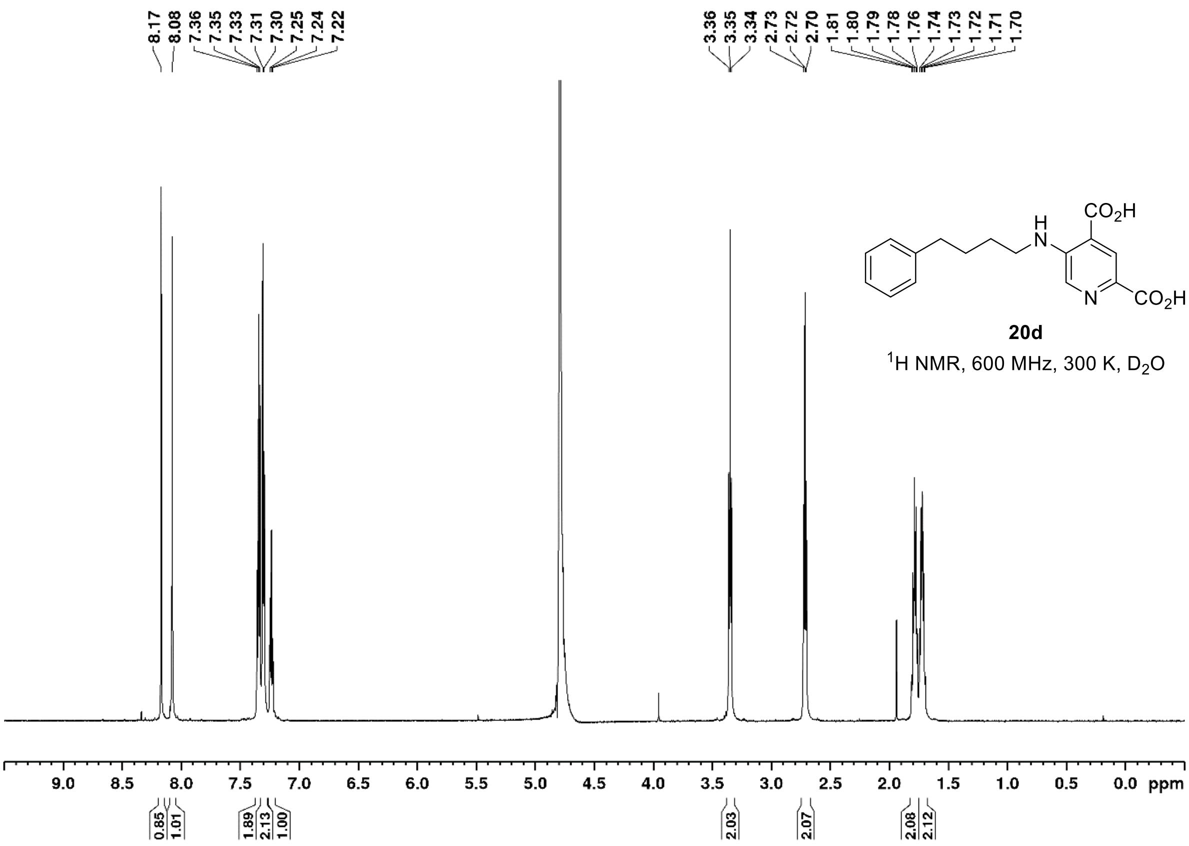
8.17 8.08 7.36 7.35 7.33 7.31 7.30 7.25 7.24 7.22
3.36 3.35 3.34 2.73 2.72 2.70 1.81 1.80 1.79 1.78 1.76 1.74 1.73 1.72 1.71 1.70
CO_2H
H
N
N
CO_2H
20d
^{1}H NMR, 600 MHz, 300 K, D_2O
9.0 8.5 8.0 7.5 7.0 6.5 6.0 5.5 5.0 4.5 4.0 3.5 3.0 2.5 2.0 1.5 1.0 0.5 0.0 ppm
0.85 1.01 1.89 2.13 1.00 2.03 2.07 2.08 2.12

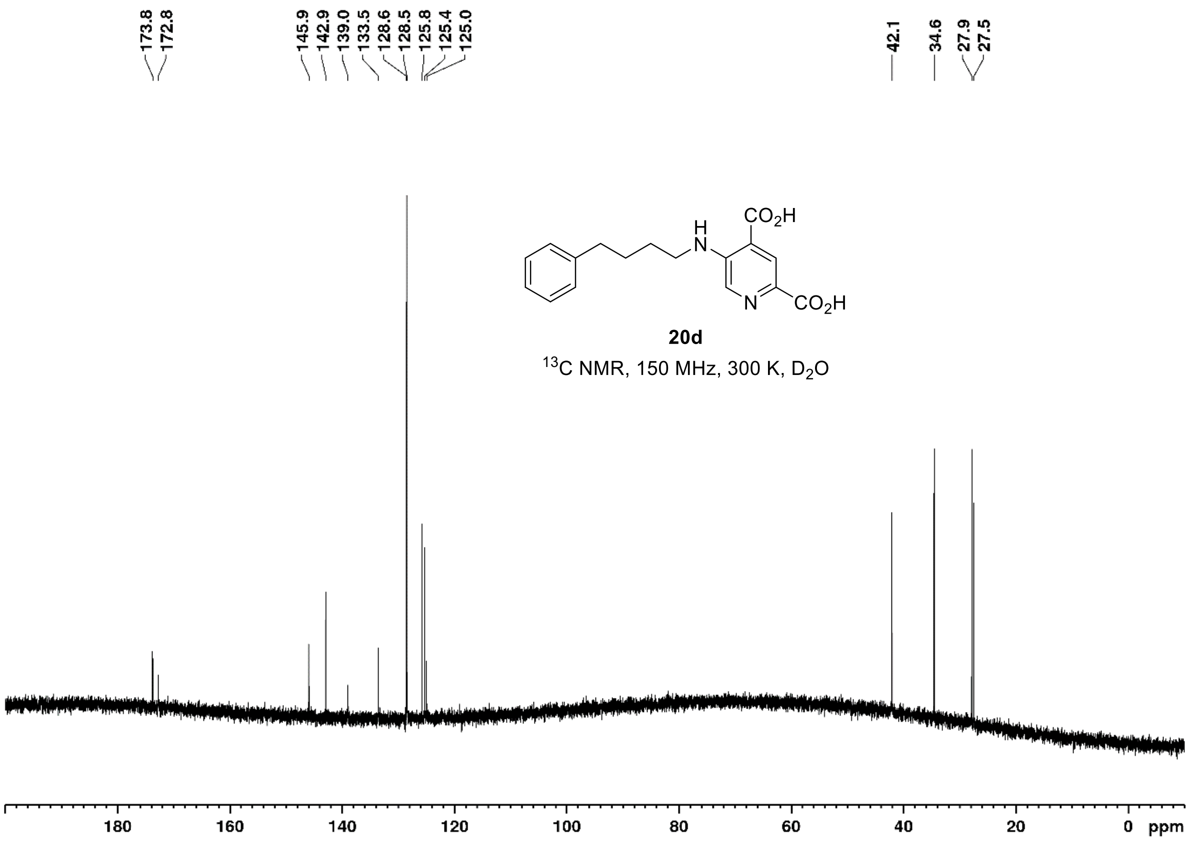
173.8 172.8
145.9 142.9 139.0 133.5 128.6 128.5 125.8 125.4 125.0
42.1 34.6 27.9 27.5
CO_2H
H
N
N
CO_2H
20d
^{13}C NMR, 150 MHz, 300 K, D_2O
180 160 140 120 100 80 60 40 20 0 ppm

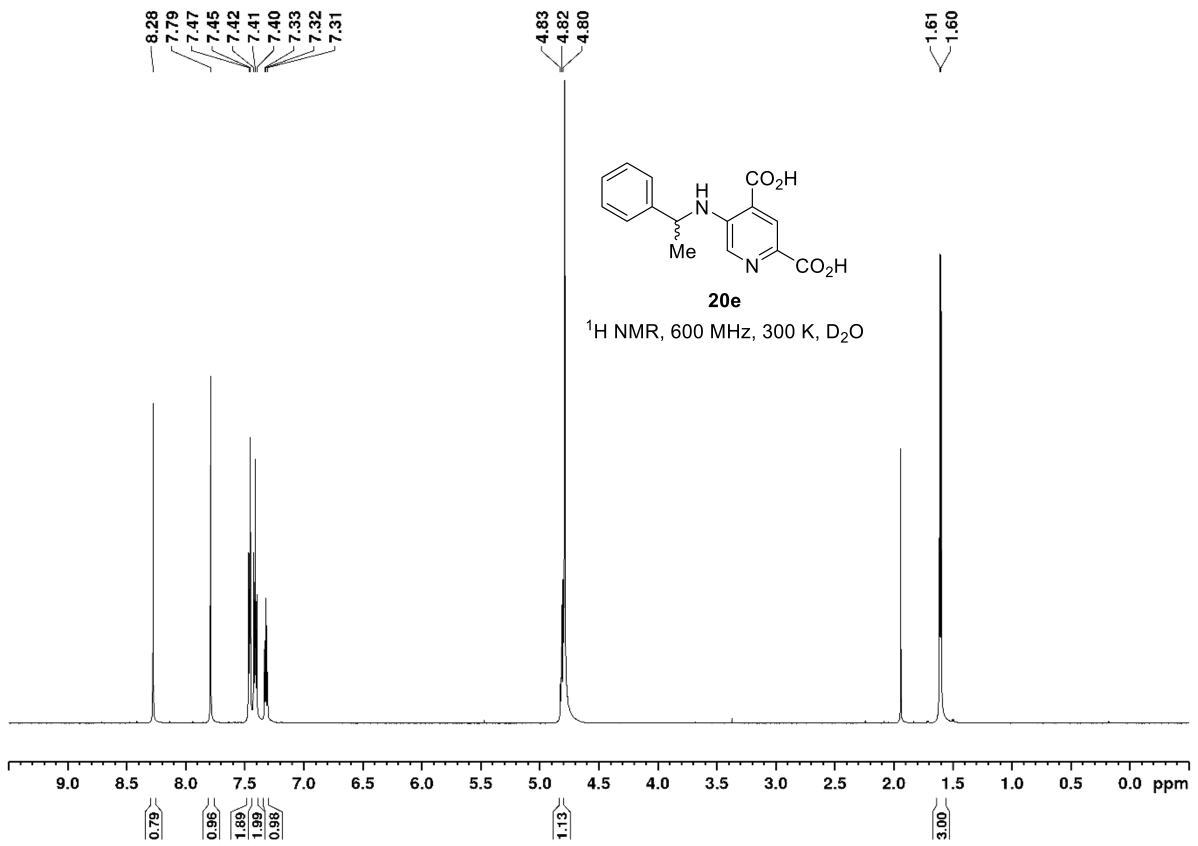
8.28
7.79
7.47
7.45
7.42
7.41
7.40
7.33
7.32
7.31
4.83
4.82
4.80
1.61
1.60
H
N
CO2H
Me
N
CO2H
20e
1H NMR, 600 MHz, 300 K, D2O
9.0 8.5 8.0 7.5 7.0 6.5 6.0 5.5 5.0 4.5 4.0 3.5 3.0 2.5 2.0 1.5 1.0 0.5 0.0 ppm
0.79
0.96
1.89
1.99
0.98
1.13
3.00

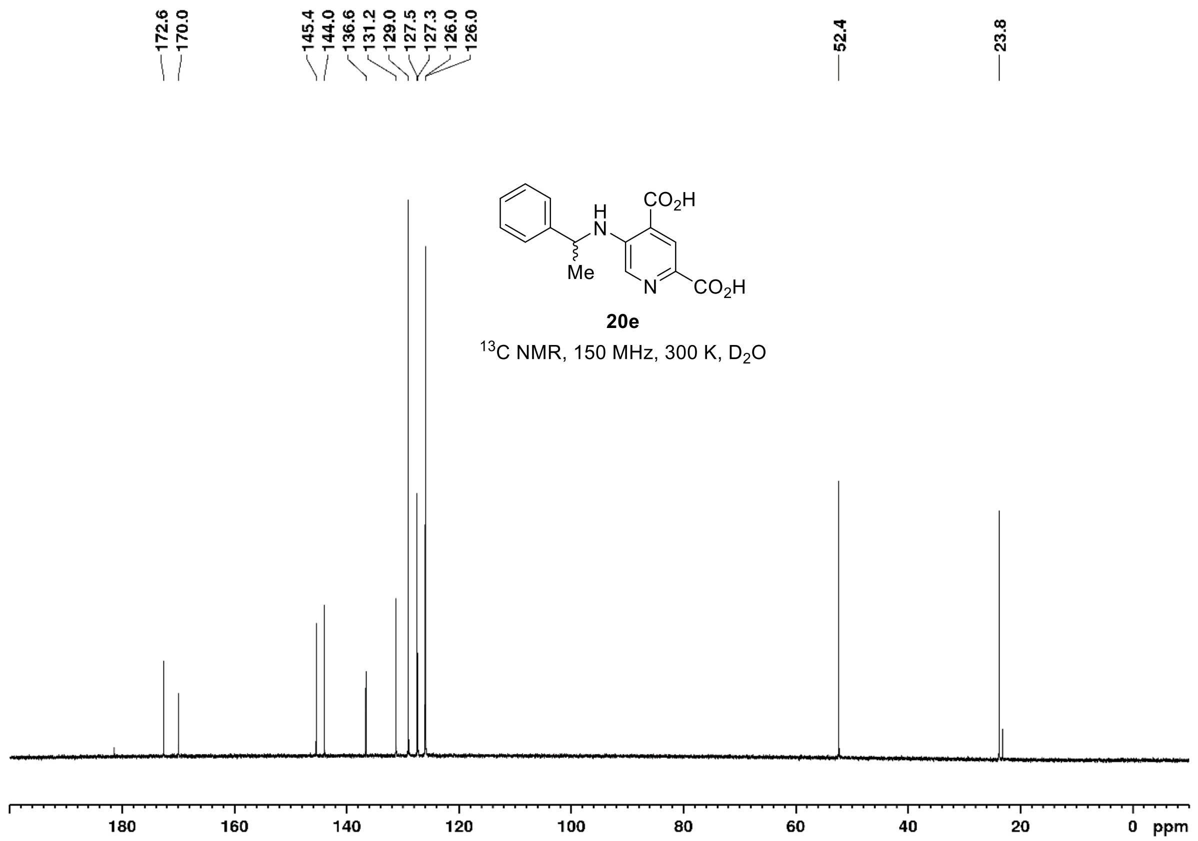
172.6
170.0
145.4
144.0
136.6
131.2
129.0
127.5
127.3
126.0
126.0
52.4
23.8
H
N
CO2H
Me
N
CO2H
20e
13C NMR, 150 MHz, 300 K, D2O
180 160 140 120 100 80 60 40 20 0 ppm

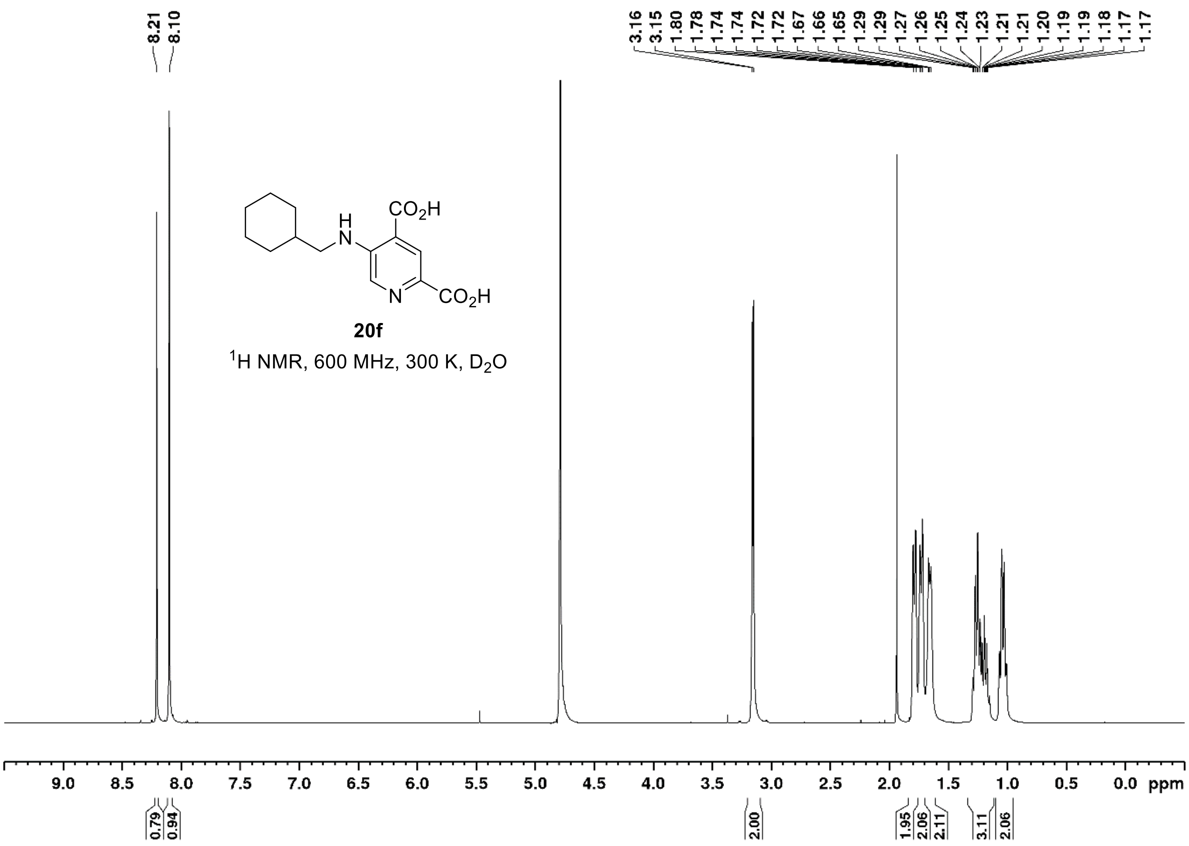
3.16 3.15 1.80 1.78 1.74 1.74 1.72 1.72 1.67 1.66 1.65 1.29 1.29 1.27 1.26 1.25 1.24 1.23 1.21 1.21 1.20 1.19 1.19 1.18 1.17 1.17
8.21 8.10
CO_2H
H
N
N
CO_2H
20f
^1H NMR, 600 MHz, 300 K, D_2O
9.0 8.5 8.0 7.5 7.0 6.5 6.0 5.5 5.0 4.5 4.0 3.5 3.0 2.5 2.0 1.5 1.0 0.5 0.0 ppm
0.79 0.94 2.00 1.95 2.06 2.11 3.11 2.06

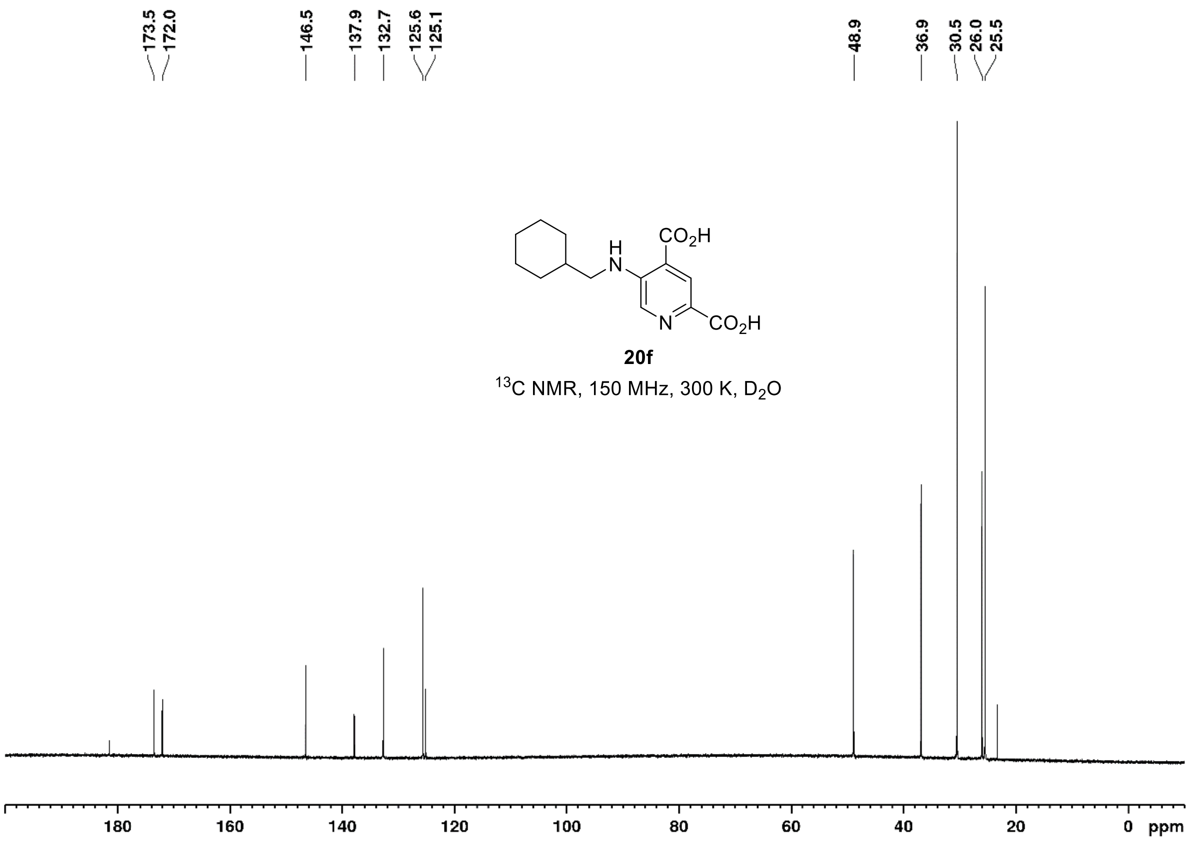
173.5 172.0 146.5 137.9 132.7 125.6 125.1 48.9 36.9 30.5 26.0 25.5
CO_2H
H
N
N
CO_2H
20f
^{13}C NMR, 150 MHz, 300 K, D_2O
180 160 140 120 100 80 60 40 20 0 ppm

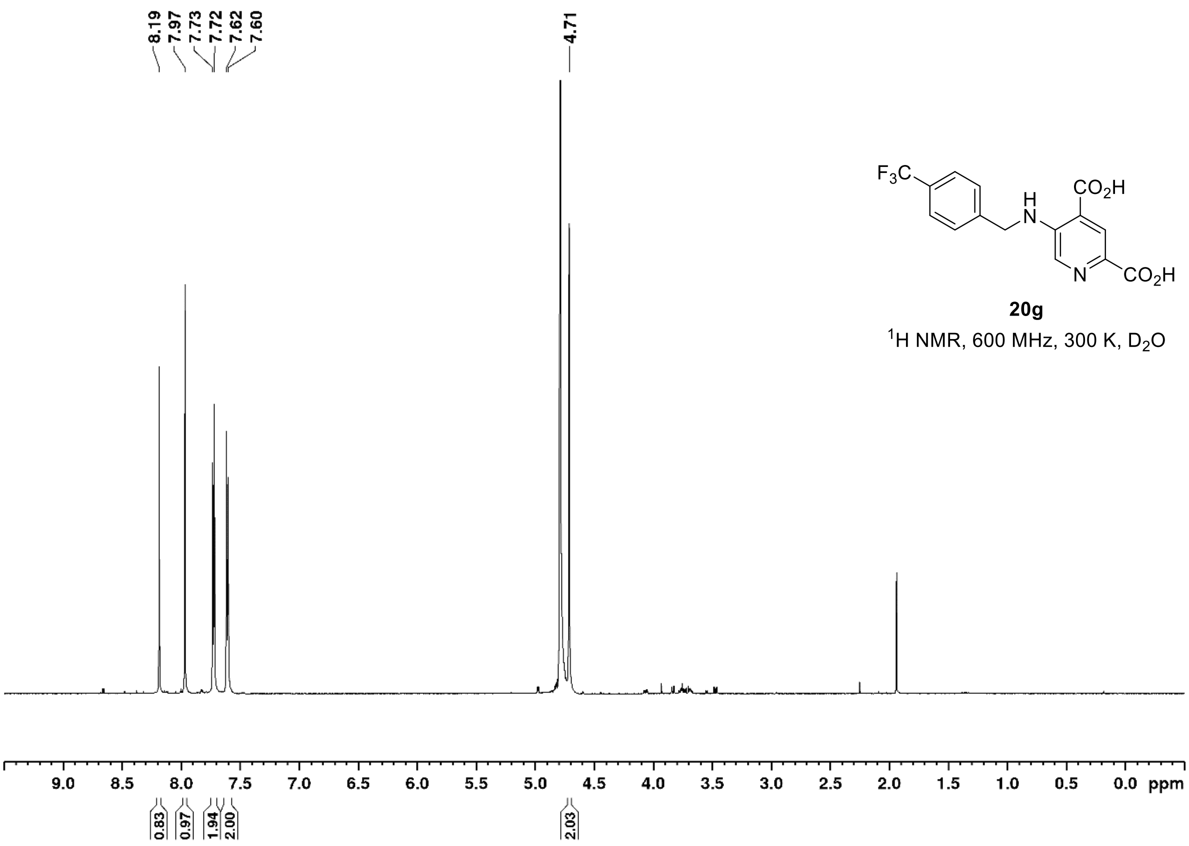
8.19
7.97
7.73
7.72
7.62
7.60
4.71
F_3C
H
N
CO_2H
N
CO_2H
20g
^1H NMR, 600 MHz, 300 K, D_2O
9.0 8.5 8.0 7.5 7.0 6.5 6.0 5.5 5.0 4.5 4.0 3.5 3.0 2.5 2.0 1.5 1.0 0.5 0.0 ppm
0.83
0.97
1.94
2.00
2.03

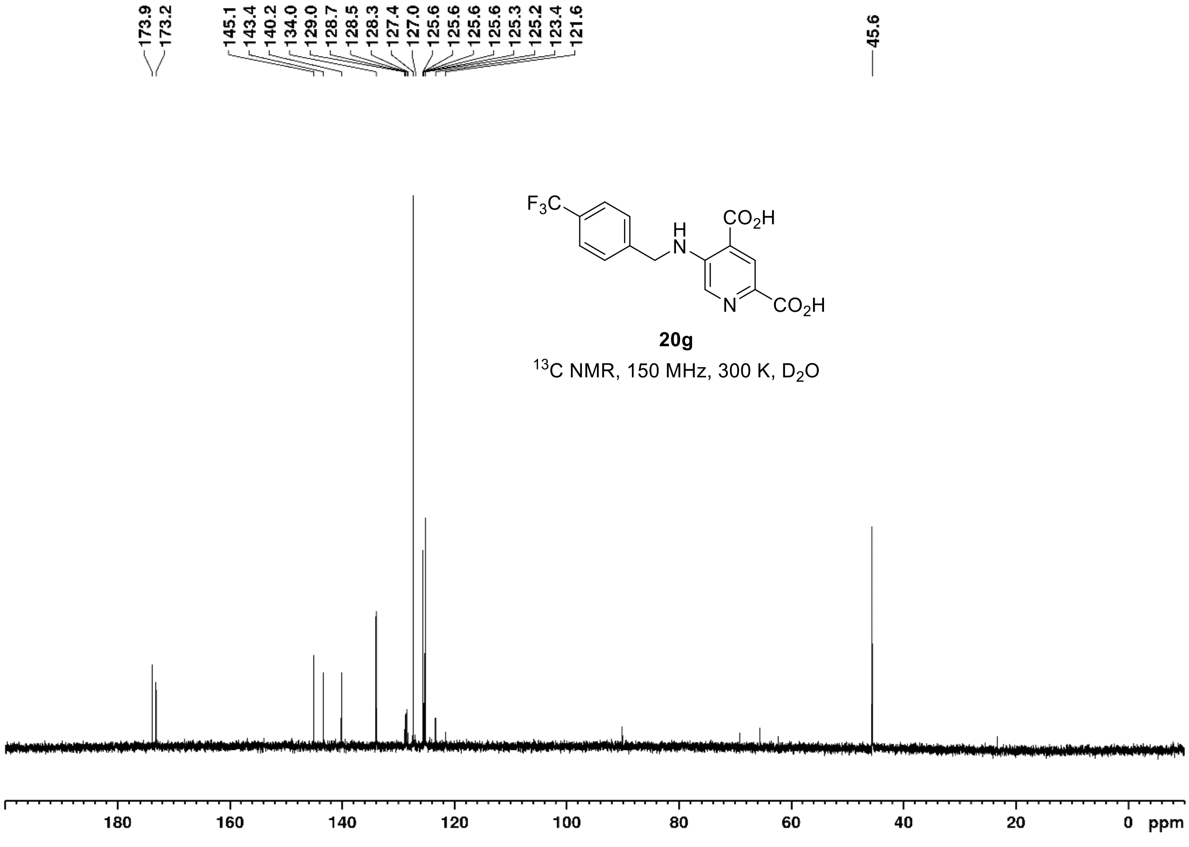
173.9
173.2
145.1
143.4
140.2
134.0
129.0
128.7
128.5
128.3
127.4
127.0
125.6
125.6
125.6
125.6
125.3
125.2
123.4
121.6
45.6
F_3C
H
N
CO_2H
N
CO_2H
20g
^{13}C NMR, 150 MHz, 300 K, D_2O
180 160 140 120 100 80 60 40 20 0 ppm

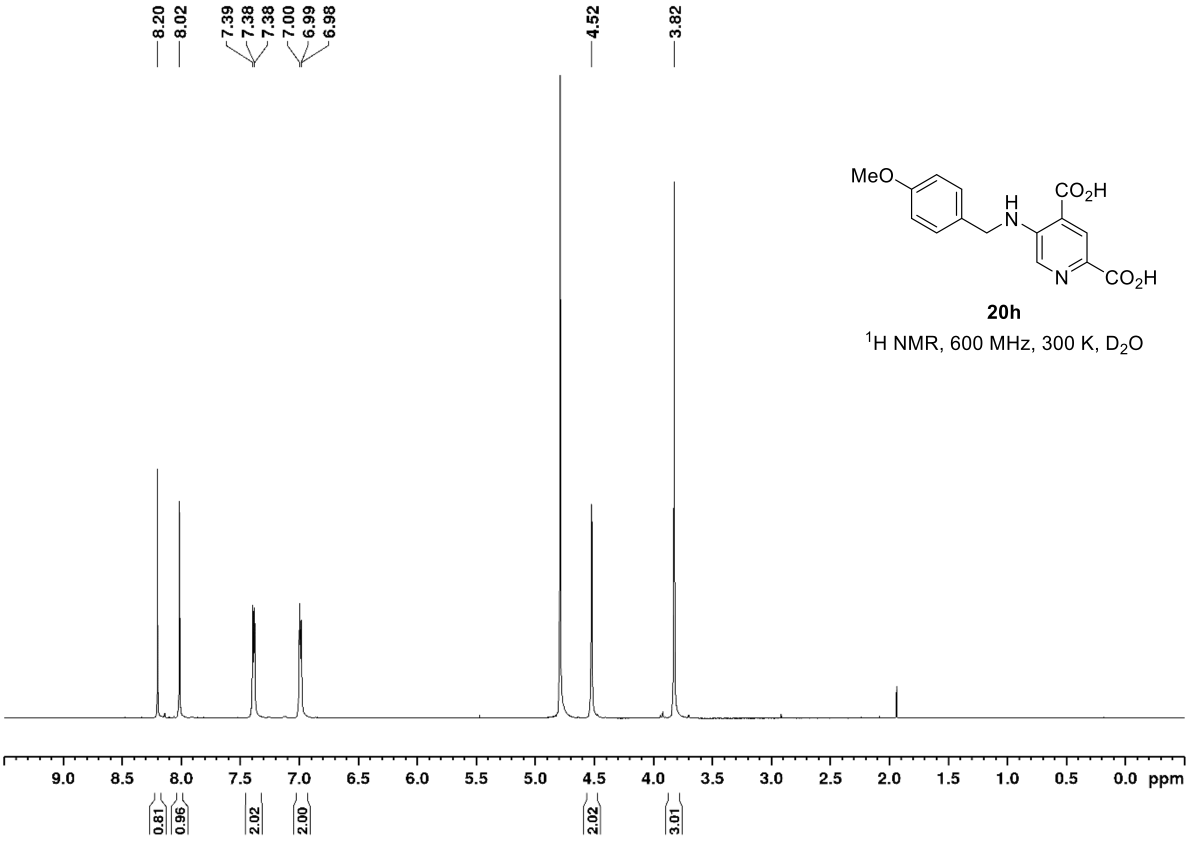
8.20
8.02
7.39
7.38
7.38
7.00
6.99
6.98
4.52
3.82
MeO
H
N
CO_2H
N
CO_2H
20h
^{1}H NMR, 600 MHz, 300 K, D_2O
9.0 8.5 8.0 7.5 7.0 6.5 6.0 5.5 5.0 4.5 4.0 3.5 3.0 2.5 2.0 1.5 1.0 0.5 0.0 ppm
0.81
0.96
2.02
2.00
2.02
3.01

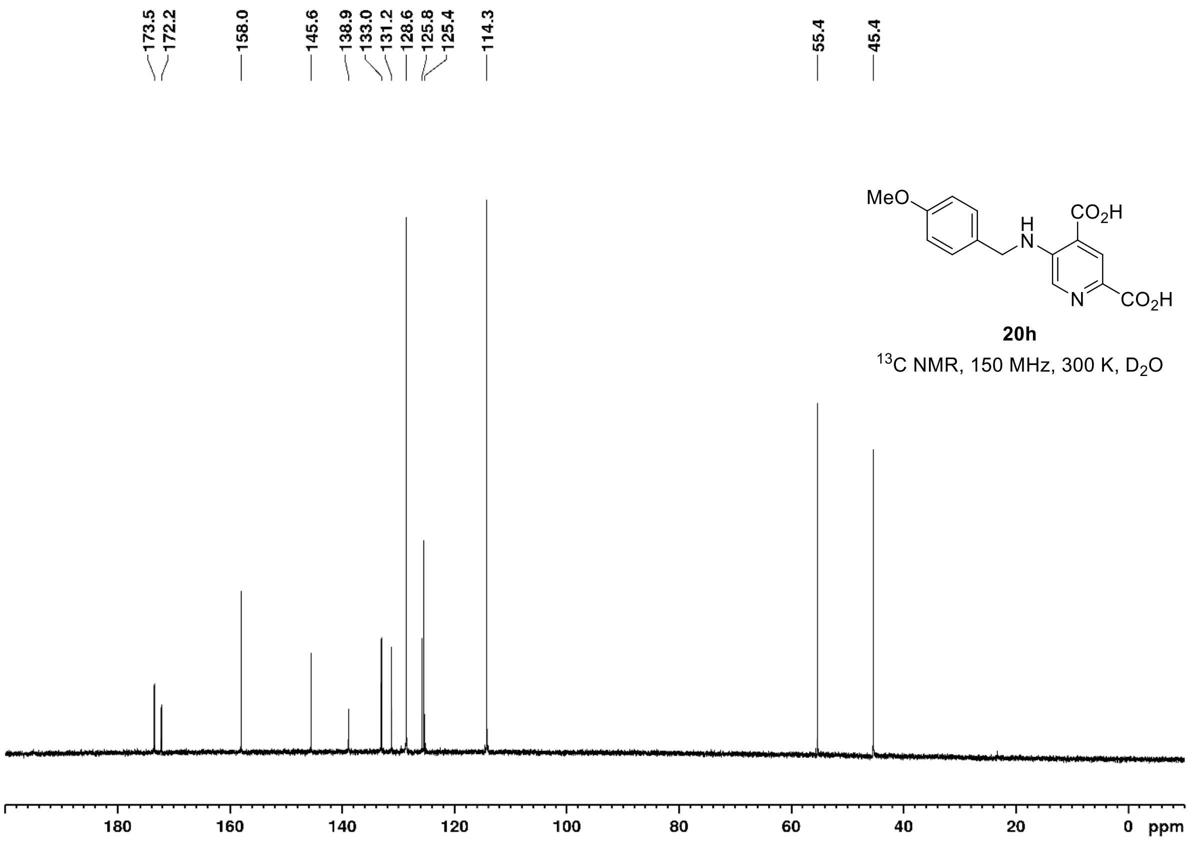
173.5
172.2
158.0
145.6
138.9
133.0
131.2
128.6
125.8
125.4
114.3
55.4
45.4
MeO
H
N
CO_2H
N
CO_2H
20h
^{13}C NMR, 150 MHz, 300 K, D_2O
180 160 140 120 100 80 60 40 20 0 ppm

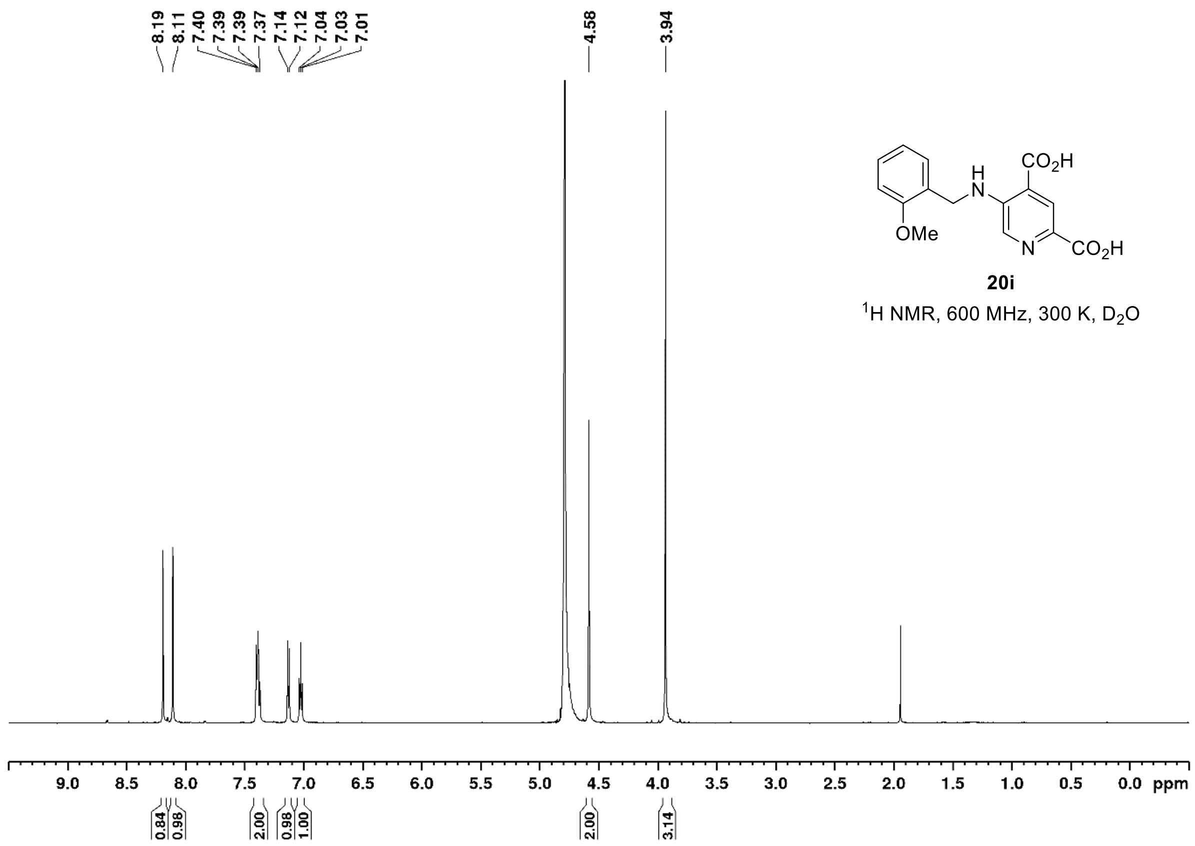
8.19
8.11
7.40
7.39
7.39
7.37
7.14
7.12
7.04
7.03
7.01
4.58
3.94
CO2H
H
N
OMe
N
CO2H
20i
1H NMR, 600 MHz, 300 K, D2O
9.0 8.5 8.0 7.5 7.0 6.5 6.0 5.5 5.0 4.5 4.0 3.5 3.0 2.5 2.0 1.5 1.0 0.5 0.0 ppm
0.84
0.98
2.00
0.98
1.00
2.00
3.14

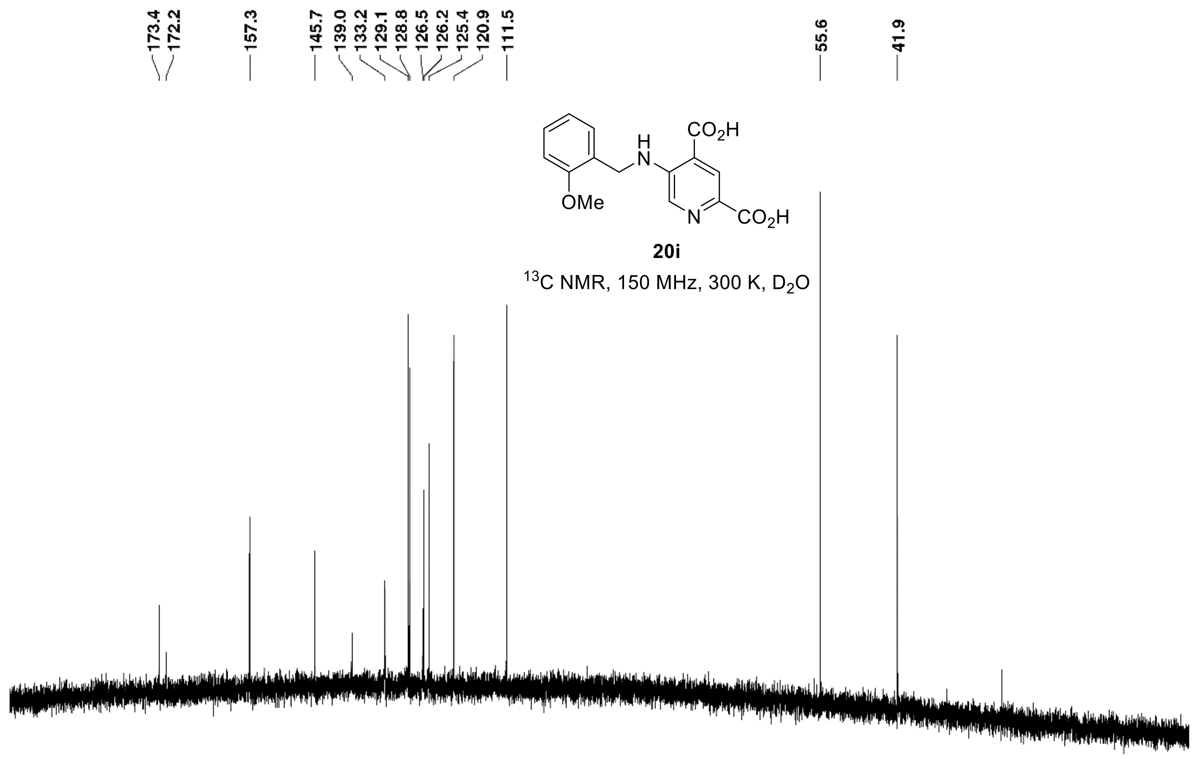
173.4
172.2
157.3
145.7
139.0
133.2
129.1
128.8
126.5
126.2
125.4
120.9
111.5
55.6
41.9
CO2H
H
N
OMe
N
CO2H
20i
13C NMR, 150 MHz, 300 K, D2O
180 160 140 120 100 80 60 40 20 0 ppm

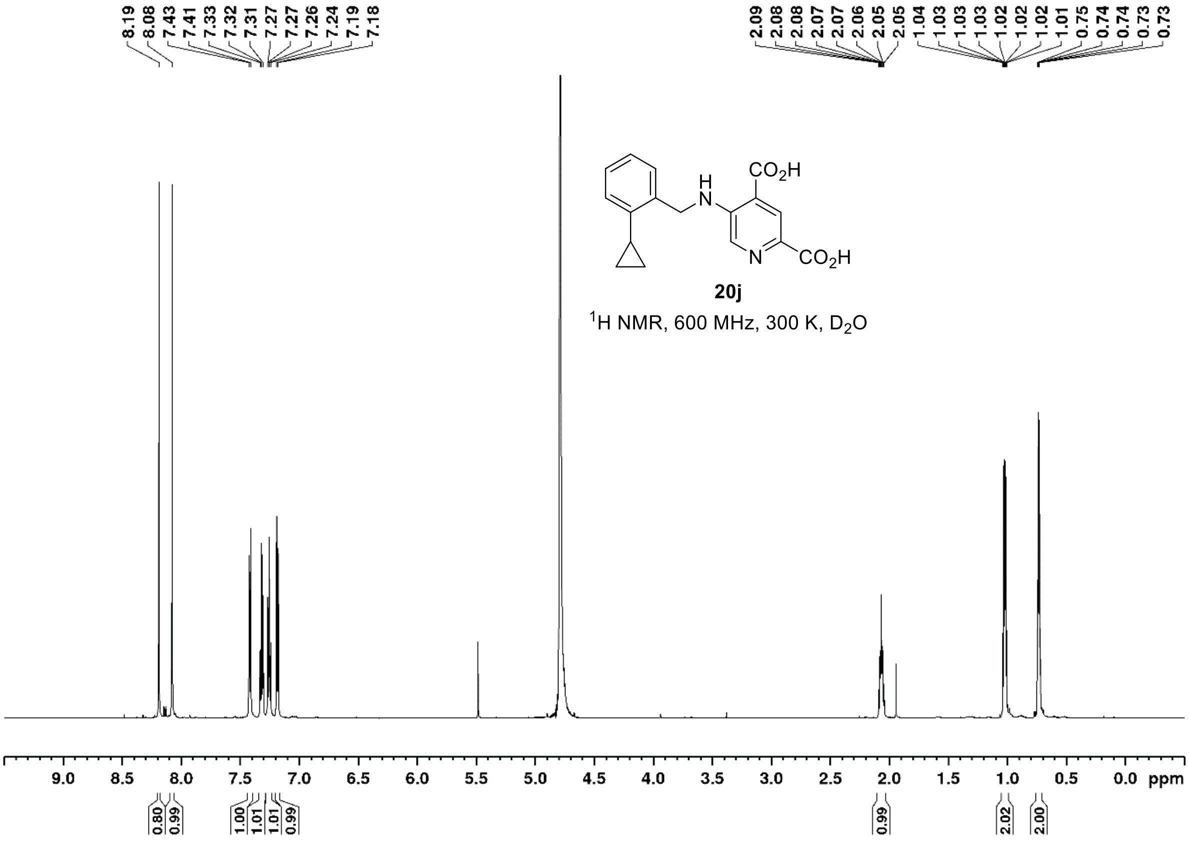
8.19 8.08 7.43 7.41 7.33 7.32 7.31 7.27 7.27 7.26 7.24 7.19 7.18
2.09 2.08 2.08 2.07 2.07 2.06 2.05 2.05 1.04 1.03 1.03 1.03 1.02 1.02 1.02 1.01 0.75 0.74 0.74 0.73 0.73
H
N
CO_2H
N
CO_2H
20j
^1H NMR, 600 MHz, 300 K, D_2O
9.0 8.5 8.0 7.5 7.0 6.5 6.0 5.5 5.0 4.5 4.0 3.5 3.0 2.5 2.0 1.5 1.0 0.5 0.0 ppm
0.80 0.99 1.00 1.01 1.01 0.99 0.99 2.02 2.00

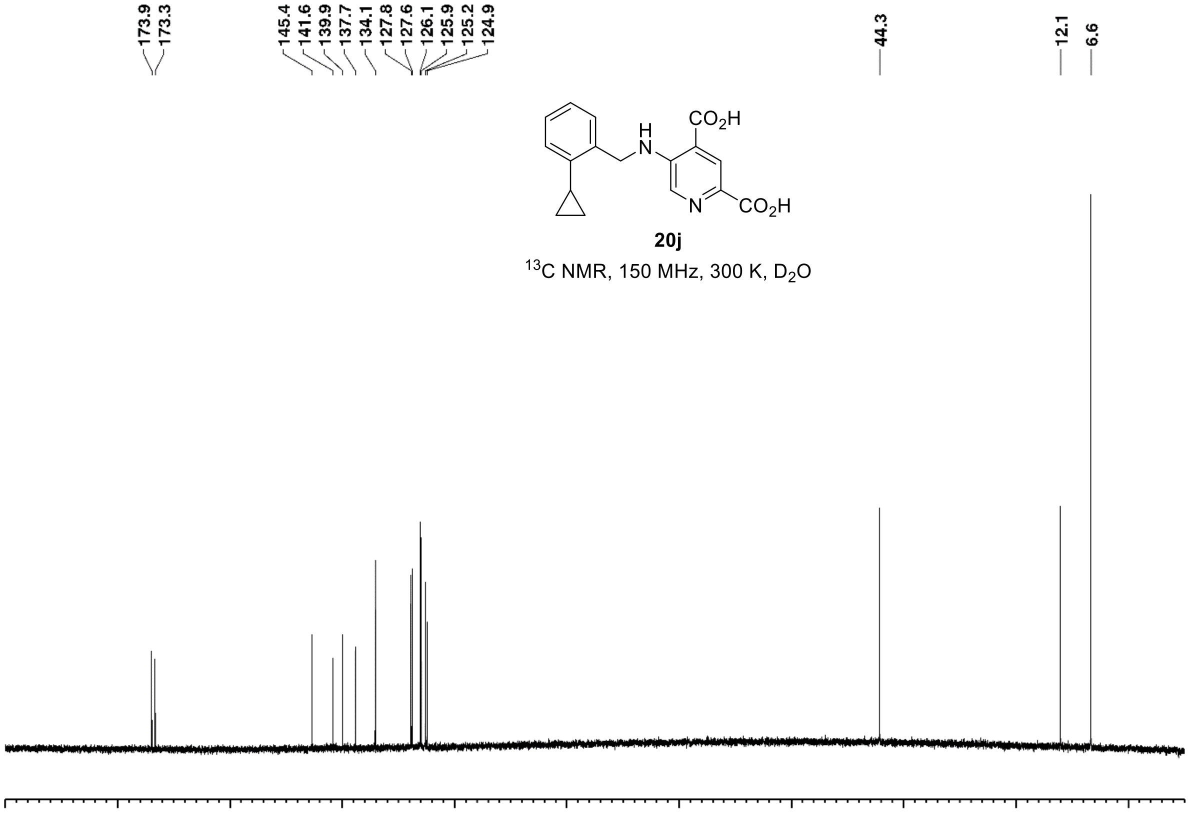
173.9 173.3
145.4 141.6 139.9 137.7 134.1 127.8 127.6 126.1 125.9 125.2 124.9
44.3
12.1
6.6
H
N
CO_2H
N
CO_2H
20j
^{13}C NMR, 150 MHz, 300 K, D_2O
180 160 140 120 100 80 60 40 20 0 ppm

8. HPLC traces of C5 substituted 2,4-PDCA derivatives prepared for this study. HPLC traces were recorded using a semi-preparative HPLC machine (Shimadzu UK Ltd.) equipped with a reverse phase column (ACE 5 C18, dimensions: 100 mm length, 21.2 mm inner diameter, 5.0 µm particle size). A linear gradient (2−98%$_{v/v}$ over 15 min) of acetonitrile in water (each containing 0.1%$_{v/v}$ trifluoroacetic acid) was used as eluent (flow rate: 12 mL/min; wavelength monitored: 220 nm). The area% of the major peak (labelled with the retention time, t_R) is ≥95% with respect to the sum of the area% of all peaks detected (excluding the injection peak at ~2.5-3.0 min).

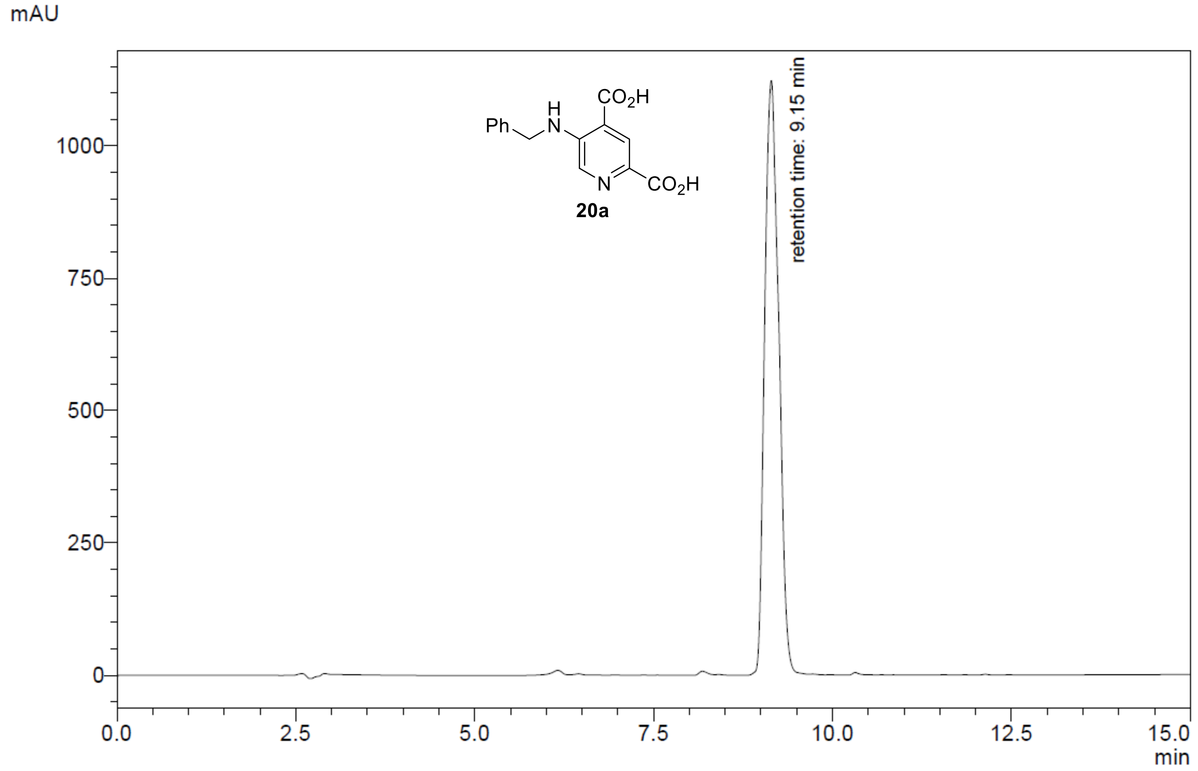

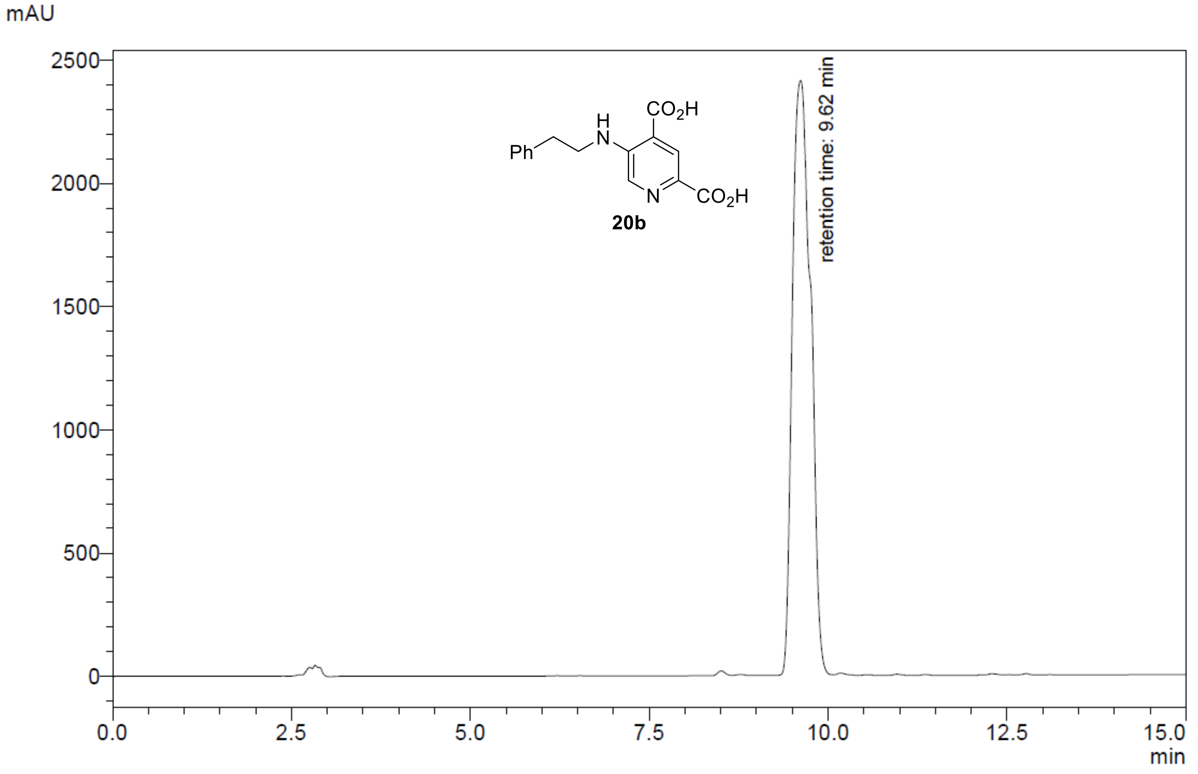

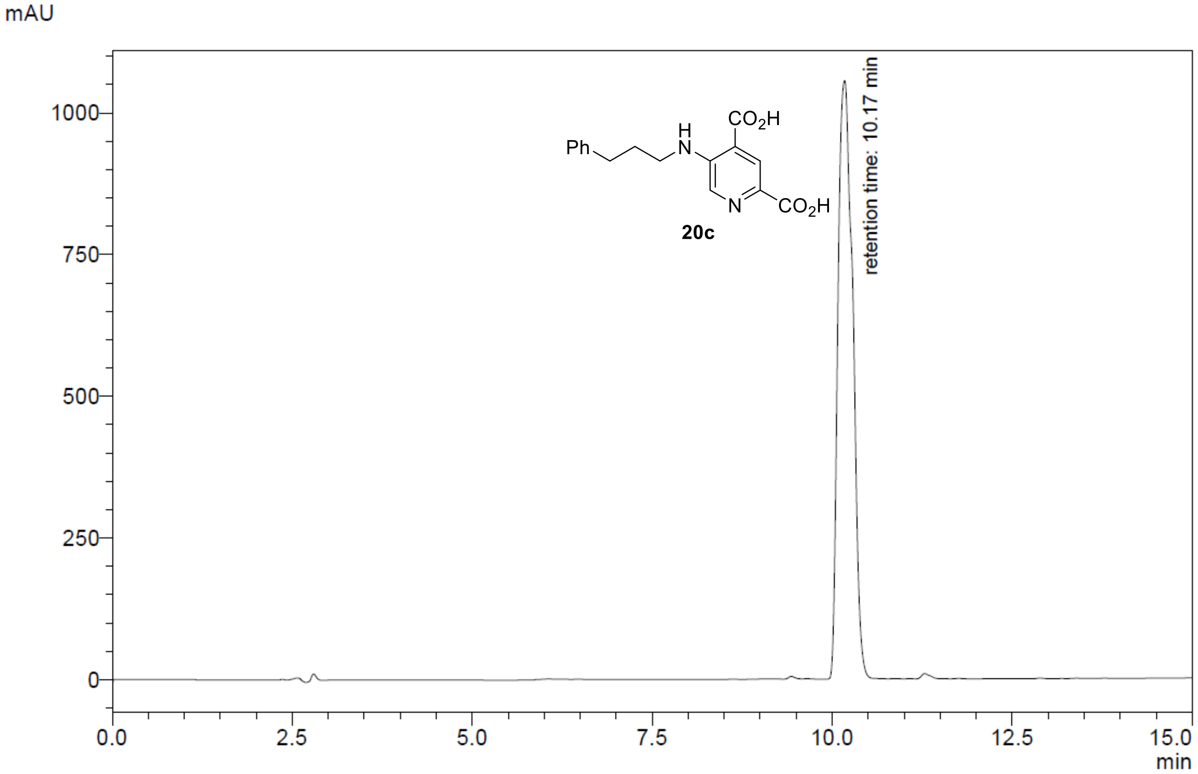
mAU
1000
750
500
250
0
0.0
2.5
5.0
7.5
10.0
12.5
15.0
min
Ph
H
N
CO2H
N
CO2H
20c
retention time: 10.17 min

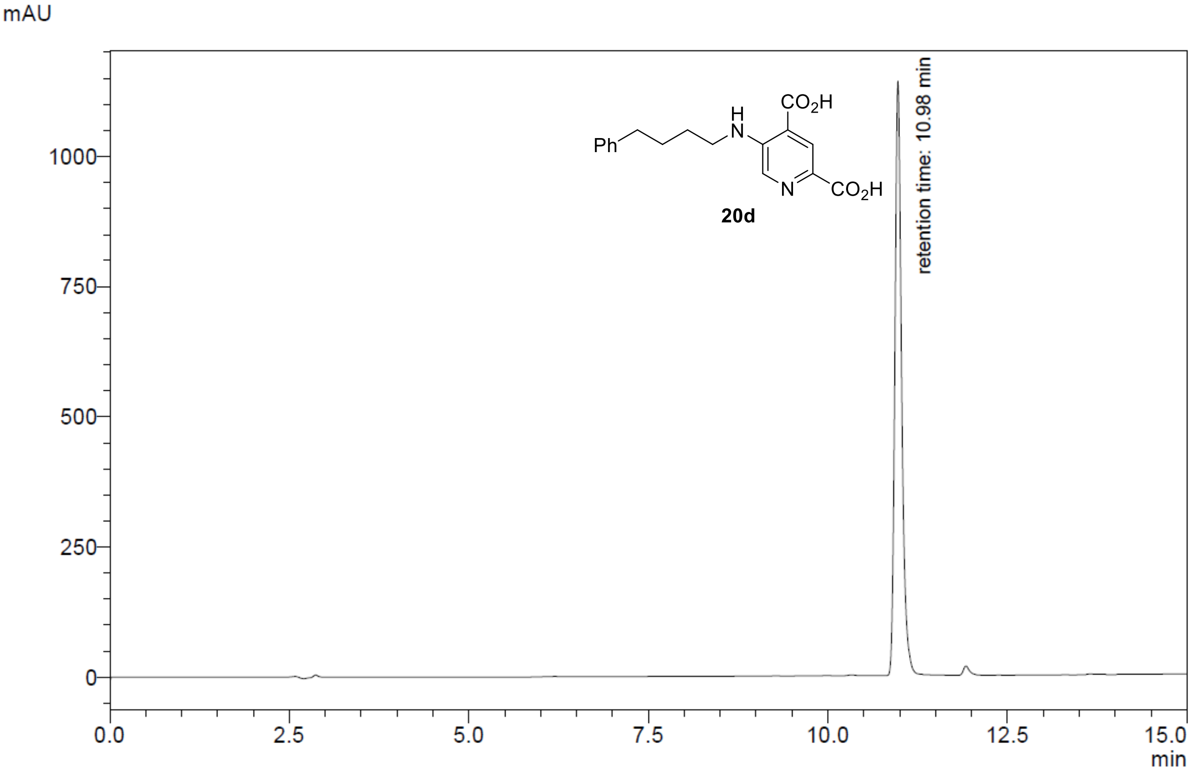
mAU
1000
750
500
250
0
0.0
2.5
5.0
7.5
10.0
12.5
15.0
min
Ph
H
N
CO2H
N
CO2H
20d
retention time: 10.98 min

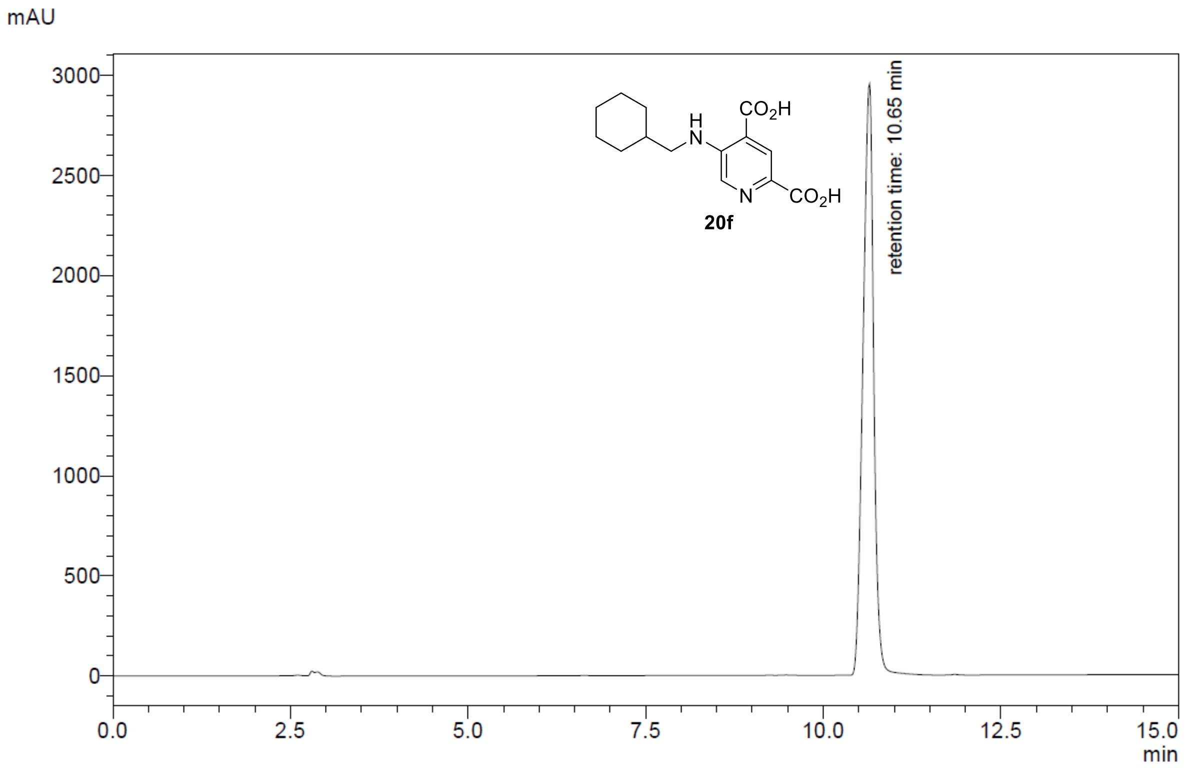
mAU
3000
2500
2000
1500
1000
500
0
0.0
2.5
5.0
7.5
10.0
12.5
15.0
min
retention time: 10.65 min
H
N
CO2H
N
CO2H
20f

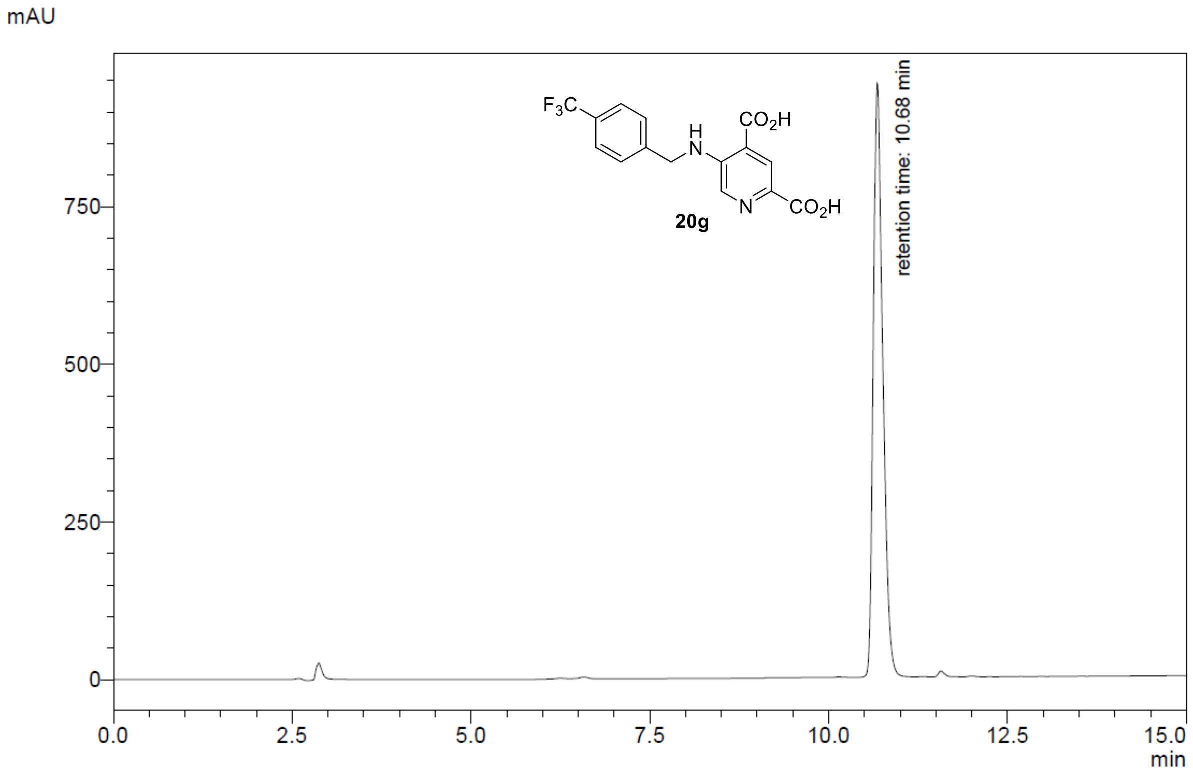
mAU
750
500
250
0
0.0
2.5
5.0
7.5
10.0
12.5
15.0
min
retention time: 10.68 min
F3C
H
N
CO2H
N
CO2H
20g

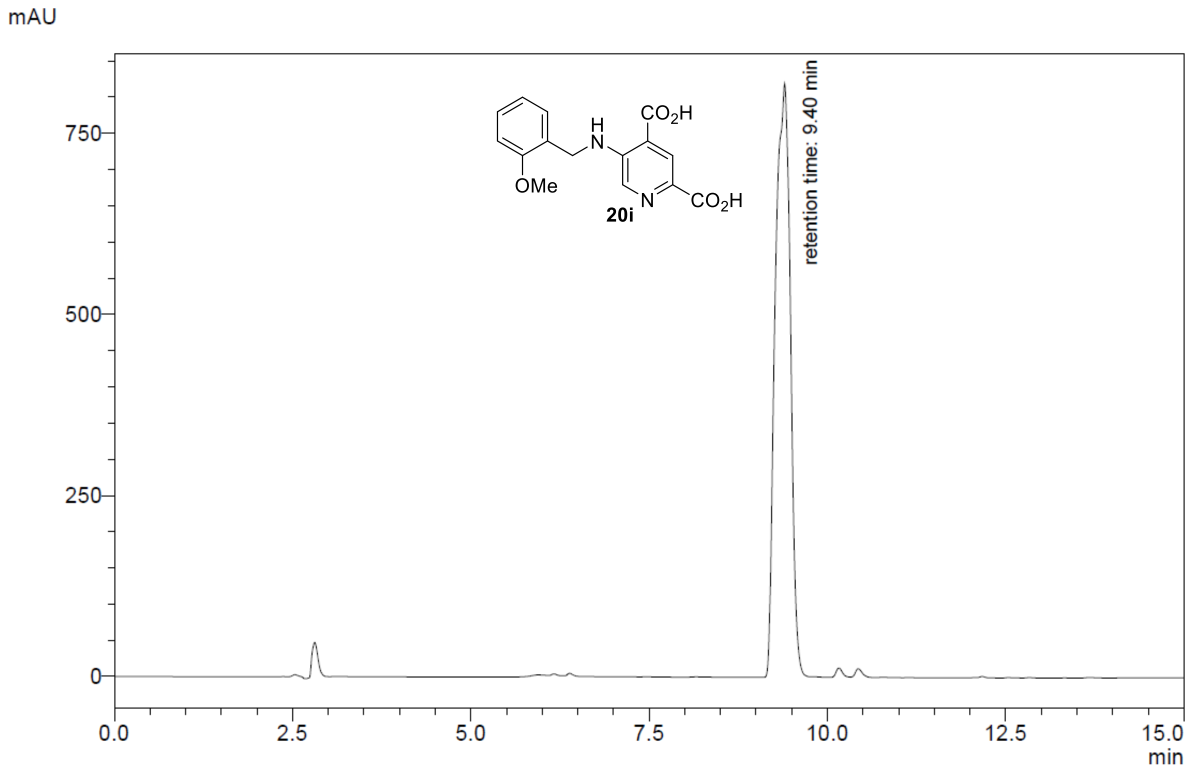
mAU
750
500
250
0
0.0
2.5
5.0
7.5
10.0
12.5
15.0
min
retention time: 9.40 min
CO_2H
H
N
OMe
N
CO_2H
20i

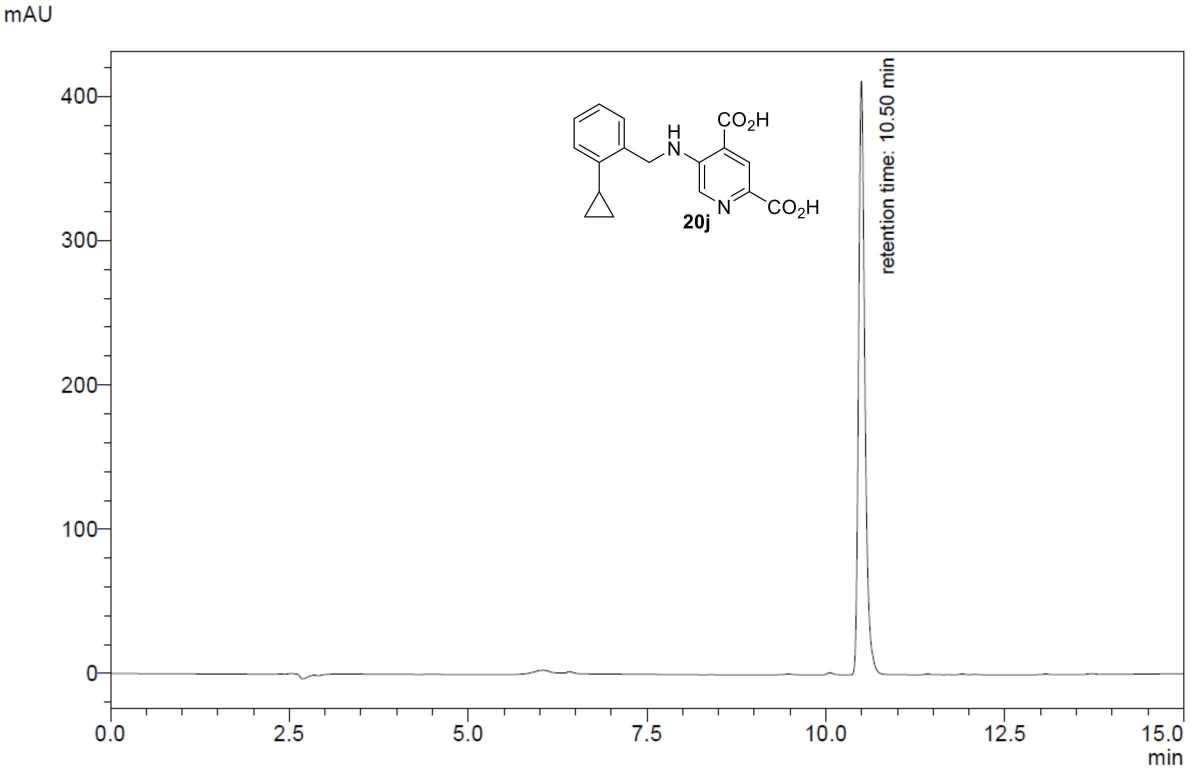
mAU
400
300
200
100
0
0.0
2.5
5.0
7.5
10.0
12.5
15.0
min
retention time: 10.50 min
CO_2H
H
N
N
CO_2H
20j
